# Synthesis of Tertiary and Quaternary Amine Derivatives from Wood Resin as Chiral NMR Solvating Agents

**DOI:** 10.3390/molecules201119732

**Published:** 2015-11-23

**Authors:** Tiina Laaksonen, Sami Heikkinen, Kristiina Wähälä

**Affiliations:** Department of Chemistry, University of Helsinki, A.I. Virtasen aukio 1, P. O. Box 55, FI-00014, Helsinki 00100, Finland; tiina.laaksonen@helsinki.fi (T.L.); sami.heikkinen@helsinki.fi (S.H.)

**Keywords:** chiral resolution, chiral solvating agent, (+)-dehydroabietylamine, NMR spectroscopy

## Abstract

Chiral tertiary and quaternary amine solvating agents for NMR spectroscopy were synthesized from the wood resin derivative (+)-dehydroabietylamine (**2**). The resolution of enantiomers of model compounds [Mosher’s acid (**3**) and its *n*-Bu_4_N salt (**4**)] (guests) by (+)-dehydroabietyl-*N*,*N*-dimethylmethanamine (**5**) and its ten different ammonium salts (hosts) was studied. The best results with **3** were obtained using **5** while with **4** the best enantiomeric resolution was obtained using (+)-dehydroabietyl-*N*,*N*-dimethylmethanaminium bis(trifluoromethane-sulfonimide) (**6**). The compounds **5** and **6** showed a 1:1 complexation behaviour between the host and guest. The capability of **5** and **6** to recognize the enantiomers of various α-substituted carboxylic acids and their *n*-Bu_4_N salts in enantiomeric excess (ee) determinations was demonstrated. A modification of the RES-TOCSY NMR pulse sequence is described, allowing the enhancement of enantiomeric discrimination when the resolution of multiplets is insufficient.

## 1. Introduction

**Figure 1:**
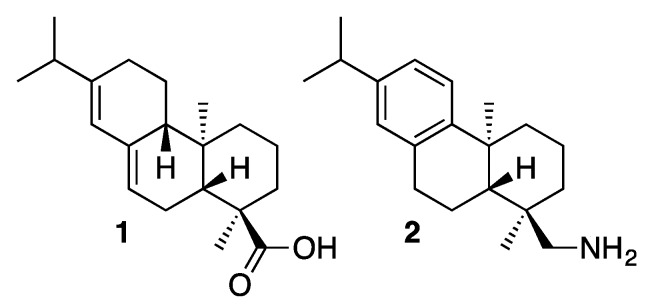
Abietic acid (**1**) and (+)-dehydroabietylamine (**2**).

Wood resin is a natural product that after losing its volatile components hardens and is then called rosin. Resin functions in wood as a protective agent against micro-organisms and as a reserve nutrient supply [1]. Resin, a mixture of compounds (e.g., resin acids, fatty acids, sitosterol) can be collected from the tree itself, but can be also obtained from the pulp industry where it is a by-product of pulp milling [2]. For example, pine resin is a mixture of resin acids (*ca.* 90%) and neutral compounds (*ca.* 10%). The most abundant resin acid in pine rosin is abietic acid (**1**, Figure 1), amounting up to 90%. Rosin can be derivatized to an amine mixture (known as Rosin Amine D) containing 60% of (+)-dehydroabietylamine (**2**, Figure 1) [3,4,5], able to resolve chiral carboxylic acids by crystallization [6,7]. We have previously reported [8] that neutral and ammonium forms of secondary amines derived from dehydroabietylamine may be used in the chiral recognition of enantiomers of certain carboxylic acids. To evaluate the corresponding tertiary amines for chiral recognitions we have examined in the present study the behaviour of dehydroabietylamine (**2**)-derived tertiary amines and their ammonium salts as chiral solvating agents (CSAs) that can be used in the discrimination of enantiomers of both aromatic and aliphatic carboxylic acids by NMR. In particular, it was of interest to develop ionic CSAs to see if the ionic functionality would provide improved enantiomeric resolution.

Especially in medicinal and biological chemistry, where enantiopurity is important, analytical applications such as NMR spectroscopy for determining the enantiomeric purity are highly useful. In NMR, enantiomeric excess (ee) may be determined up to the level of 99% but generally for ee values over 95% more sensitive chromatographic methods (e.g., chiral HPLC and GC) have been used [9,10,11,12]. Compared to the most commonly used method HPLC, the minimal sample preparation, ease of use and fast analysis make CSAs an optimal tool for expedient ee determinations.

**Figure 2:**
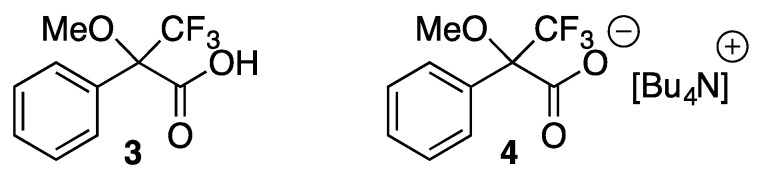
Mosher’s acid (**3**) and its *n*-Bu_4_N salt (**4**).

Enantiomeric resolution by CSAs is based on the formation of a diastereomeric complex (or diastereomeric salt) [13,14]. There are several factors that may have an effect on the complex formation. It is known in the literature that the ability of CSAs (host) to bind a chiral compound (guest) is affected by the deuterated solvent used, the possible anion of the chiral host [15], concentration, host:guest ratio, temperature and the functional groups of the host and guest [13,14]. Since polar solvents can solvate ions and protic solvents can interfere in hydrogen bond formation, it is preferable to use non-polar or weakly polar aprotic NMR solvents such as CDCl_3_ in recognition studies, especially when the analyte and CSA used are ionic (or when the formation of a diastereomeric salt pair is expected) [13]. The most frequently used compound to test the discrimination ability of a CSA, especially an ionic one, is Mosher’s acid (**3**, 3,3,3-trifluoro-2-methoxy-2-phenyl-propanoic acid, Figure 2) and its sodium or potassium salt. In our enantiomeric discrimination tests, also Mosher’s acid *n*-Bu_4_N salt (**4**, Figure 2) was employed.

With **4** being readily soluble in CDCl_3_, unlike its potassium or sodium salt, this modification obviates the use of solubilizing additives such as crown ethers [16]. We also report a modification of the RES-TOCSY 2D-NMR technique to enhance enantiomeric discrimination when the resolution of multiplets is insufficient for ee determination. In the various publications concerning the use of CSAs in NMR spectroscopy, all [17,18,19,20,21,22,23,24,25,26,27,28,29,30] or most [31,32,33,34,35,36] of the racemic carboxylic acids or salts studied contain an aromatic moiety, and acids lacking an aromatic ring are largely ignored or deemed [37] poorly resolvable at best. As described in the following, our new ionic and non-ionic tertiary amine CSA agents enable efficient enantiomeric resolution and the determination of ee values of racemic carboxylic acids or salts, including those that lack an aromatic ring.

## 2. Results and Discussion

**Scheme 1:**
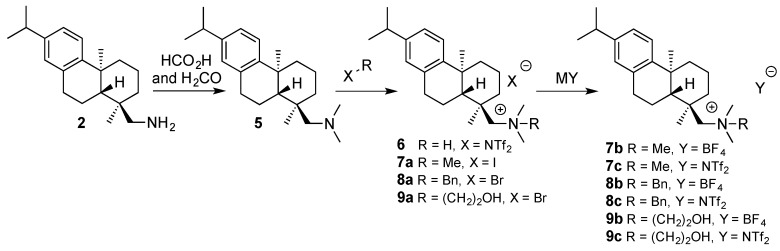
Synthesis of chiral CSAs from **2**. (MY = LiNTf_2_ or NH_4_BF_4_).

Starting material **2** was purchased as 60% grade. It was converted to its acetate salt, which was then purified by crystallisation. Pure (+)-dehydroabietyl acetate was then treated with NaOH solution to release **2** in pure form. The synthesis of CSAs based on **2** consists of two steps (Scheme 1). First, **2** is reacted with formaldehyde and formic acid to form **5** (63.8%). This tertiary amine is then quaternized with an alkyl halide under microwave irradiation. Three different alkyl halides were used: methyl iodide to obtain an uncrowded ammonium terminus (**7a**, 82.9%), 2-hydroxyethyl bromide to give hydrogen bonding capability (**8a**, 59.4%), and benzyl bromide to provide a bulky group at the ammonium terminus (**9a**, 46.5%). It is known that the more delocalised and bulky anions can enhance binding between the cationic CSA and (ionic or non-ionic) chiral substrate due to a weaker binding between the cation and anion of CSA [23]. To tune the binding properties of **7a**, **8a** and **9a**, anion exchange was performed with NH_4_BF_4_ or LiNTf_2_ to obtain **7b**, **8b**, **9b** or **7c**, **8c**, **9c**, respectively, in high yield (89.9%–97.8%). The delocalization and increased size of the anion also affect the physical properties of the ionic CSAs [23]. The melting points of ionic compounds decrease when the anion is bulky and/or weakly coordinating. Molar rotations for compounds were also determined. Alternatively, compound **5** may be protonated with bis(trifluoromethane)-sulfonimide (HNTf_2_) to give **6**. Table 1 presents the collected physical property data of the compounds synthesized.

**Table 1:** Physical properties of compounds prepared from **2**.

CSA	R	X	mp (°C)	${\text{[α]}}_{\text{D}}^{23}$ ^(a)^	${\left[M\right]}_{\text{D}}^{23}$ ^(b)^
**5**	-	-	liquid at rt.	+52.05 ^(c)^	+161.71
**6**	H	NTf_2_	114.2	+7.45	+44.29
**7a**	Me	I	250.3	+20.91	+95.19
**7b**	Me	BF_4_	246.8	+19.93 ^(d)^	+82.77
**7c**	Me	NTf_2_	46.6	+7.79	+47.41
**8a**	Bn	Br	188.6	+7.19	+34.83
**8b**	Bn	BF_4_	179.9	+2.28	+11.18
**8c**	Bn	NTf_2_	48.8	+1.70	+11.67
**9a**	(CH_2_)_2_OH	Br	213.5	+16.45 ^(e)^	+72.12
**9b**	(CH_2_)_2_OH	BF_4_	188.5	+18.73	+83.43
**9c**	(CH_2_)_2_OH	NTf_2_	viscous liquid at rt.	+6.56	+41.90

^(a)^ ${\text{[α]}}_{\text{D}}^{23}$ = deg cm^3^·g^−1^·dm^−1^, c = 1.0 g·cm^−3^, CHCl_3_; ^(b)^ Molar rotation calculated from ${\text{[α]}}_{\text{D}}^{23}$ × *M*/100, where *M* is the molar mass; ^(c)^ Temperature 22 °C; ^(d)^ Solvent CHCl_3_ + 3% MeOH; ^(e)^ Solvent MeOH.

To investigate the ability of the synthesized compounds **5–9c** to resolve ionic and non-ionic racemic carboxylic acids both **3** and **4** were used as model compounds as 22.0 mM solutions in CDCl_3_. Compounds **5–9c** (1.0 eq or 2.0 eq) were dissolved in 0.5 mL (1.0 eq) of the prepared solution and ^1^H- and ^19^F-NMR spectra were recorded (Supplementary Material Figures S1 and S2). Both 1:1 and 2:1 host:guest molar ratios were studied since according to literature, the magnitude of non-equivalence (Δδ) increases when the amount of host is higher than that of the guest [13,14]. Compounds **5** and **6** gave the best results both in 1:1 and 2:1 host:guest ratios (Table 2). The former efficiently resolved the enantiomers of **3** according to ^1^H- and ^19^F-NMR. The magnitude of non-equivalence was 0.036 ppm (18.1 Hz) in ^1^H-NMR and 0.12 ppm (58.5 Hz) in ^19^F-NMR. Only very weak resolution was achieved when compound **5** was used in the enantiomeric resolution of **4**. However, compound **6** resolved **4** highly efficiently, 0.030 ppm (15.0 Hz) in ^1^H-NMR and 0.10 ppm (48.3 Hz) in ^19^F-NMR. No resolution was detected when **6** and **3** were used as host and guest. It is not surprising that the non-ionic host resolves poorly an ionic guest and *vice versa* since no ionic interaction can exist between them. This indicates that the resolution is based on the formation of a diastereomeric salt pair. Among the quaternized amine compounds only **8a**, **8c** and **9c** were able to differentiate the enantiomers of **4** while **7c**, **8b**, **8c** and **9c** differentiated enantiomers of **3**. Compared to **5** and **6** the resolution obtained was not as high (between 8.3 and 0.8 Hz). Interestingly the quaternization of the tertiary amine seems to make the recognition of enantiomers unfeasible. This may be due to a crowding in the active bonding site, *i.e.*, ammonium terminus, caused by the side chain (methyl, benzyl, 2-hydroxyethyl) so that the guest cannot get close enough. In regard to this it is surprising that **7a–c** (max. 1.3 Hz) are less effective than **8a–c** (max. 8.3 Hz) and **9a–c** (max. 8.2 Hz). This may be caused not only by the crowding but also by the failure of the methyl group (**7a–c**) to give specific enough resolution between the enantiomers compared e.g., to benzyl (**8a–c**) and hydroxyethyl (**9a–c**) groups. When the concentration of CSA was increased (host:guest ratio 2:1) no significant increase in the Δδ was observed although in the case of **5** Δδ was increased by 3.5 Hz in ^1^H-NMR and 13.2 Hz in ^19^F-NMR. Interestingly, in the case of **6** Δδ decreased in ^1^H-NMR by 1.9 Hz although it increased in ^19^F-NMR by 5.9 Hz.

**Table 2:** Determination of the magnitude of non-equivalence (Δδ) with **3** and **4** as model compounds in the presence of different **2**-based CSAs, using both ^1^H (500 MHz) and ^19^F-NMR (470 MHz) in CDCl_3_ at 27 °C (-indicates resolution not detected).

CSA	Host:Guest	3 (ppm, (Hz))		4 (ppm, (Hz))	
		^1^H	^19^F	^1^H	^19^F
**5**	1:1	0.036 (18.1)	0.12 (58.5)	0.0018 (0.9)	-
	2:1	0.042 (21.6)	0.15 (71.7)	-	-
**6**	1:1	-	-	0.030 (15.0)	0.10 (48.3)
	2:1	-	-	0.026 (13.1)	0.12 (54.2)
**7a**	1:1	-	-	-	-
	2:1	-	-	-	-
**7b**	1:1	-	-	-	-
	2:1	-	-	-	-
**7c**	1:1	0.027 (1.3)	-	-	-
	2:1	-	-	-	-
**8a**	1:1	-	-	-	-
	2:1	-	-	-	0.015 (6.8)
**8b**	1:1	0.0015 (0.8)	-	-	-
	2:1	-	-	-	-
**8c**	1:1	0.0017 (0.9)	-	0.0037 (1.9)	-
	2:1	-	-	0.010 (5.1)	0.018 (8.3)
**9a**	1:1	-	-	-	-
	2:1	-	-	-	-
**9b**	1:1	-	-	-	-
	2:1	-	-	-	-
**9c**	1:1	0.0017 (0.9)	-	0.0052 (2.6)	-
	2:1	-	-	0.016 (8.2)	-

**Figure 3:**
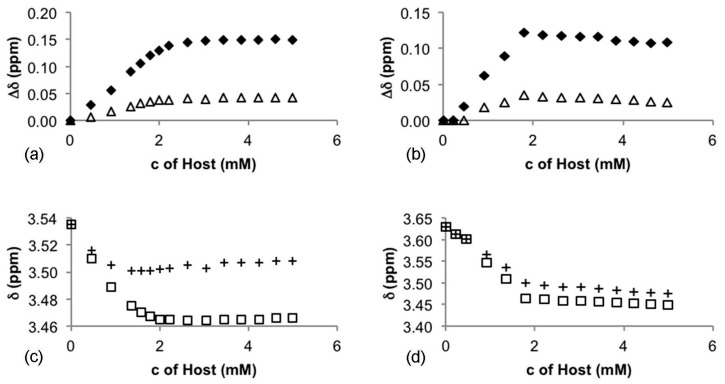
The magnitude of non-equivalence (Δδ): (**a**) of **3** with **5** (white triangle: ^1^H-NMR and black diamond: ^19^F-NMR) and (**b**) of **4** with **6** (white triangle: ^1^H-NMR and black diamond: ^19^F-NMR). The change of chemical shifts of *S* and *R* enantiomers: (**c**) of **3** as a function of the concentration of **5** (white square: *R* enantiomer and plus sign: *S* enantiomer) at ^1^H-NMR; and (**d**) of **4** as a function of the concentration of **6** (white square: *R* enantiomer and plus sign: *S* enantiomer) at ^1^H-NMR.

The discrimination ability of the best performing CSAs **5** and **6** was further investigated by titration to find the optimum conditions. Since **5** gave best resolution for **3**, and **6** for **4**, **3** was titrated by **5** and **4** was titrated by **6**. The guest solution (0.5 mL, 2.0 mM) was measured into an NMR tube and titrated with the host solution (46.6 mM). NMR spectra were recorded and Δδ **3** a **4** (both in ^1^H- and ^19^F-NMR) shown in Figure 3a,b. Results from ^1^H- and ^19^F-NMR spectra suggest that a maximum resolution with **5** and **6** is obtained when the concentrations of host and guest are the same (2.0 mM) indicating a 1:1 complexation of host and guest (Job’s plot in Supplementary material Figures S8 and S12). According to the results both enantiomers of **3** interact with **5** (and both enantiomers of **4** with **6**). This can be detected from the chemical shift changes experienced by both the *S* and *R* enantiomers (Figure 3c,d) during the titration.

**Figure 4:**
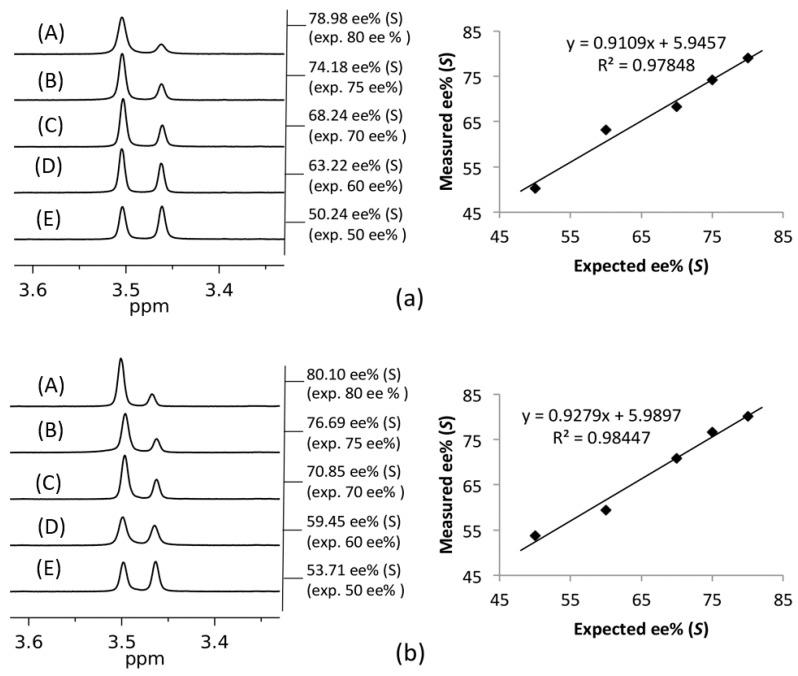
Determination of enantiomeric purities of (**a**) **3** in the presence of **5** and (**b**) **4** in the presence of **6** by ^1^H-NMR (500 MHz) in CDCl_3_ at 27 °C. (Expected ee% (*S*): (A) 80, (B) 75, (C) 70, (D) 60, (E) 50).

The possibility of using compounds **5** and **6** in enantiomeric excess (ee) measurements by NMR spectroscopy was also tested using solutions of racemic **3** and *S*-**3** (as well as **4** and *S*-**4**) (2.0 mM). Mixtures of enantiomerically enriched samples were prepared in an NMR tube (0.5 mL, 1.0 eq) and CSA (46.6 mM, 22.5 μL, 1.0 eq) was added. A clear correlation was detected between the expected and measured ee% values, when just one equivalent of host with respect to the guest in a CDCl_3_ solution was used (Figure 4). Line shape fitting was used for ee estimation to enhance accuracy.

**Figure 5:**
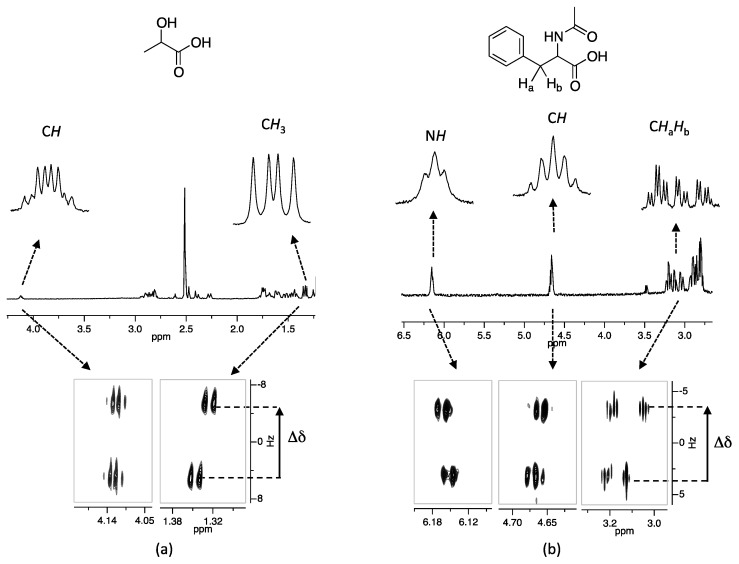
RES-TOCSY experiment performed on (**a**) **13a** (Δδ 11.1 Hz, CH_3_) and (**b**) **14a** (Δδ 6.1 Hz, CH) with CSA **5** showing the resolution of *R* and *S* enantiomer in an indirect dimension.

The resolution of various racemic aromatic or non-aromatic α-substituted carboxylic acids **10a–16a**, or their *n*-Bu_4_N salts **10b–16b**, by **5** and **6** was tested (Table 3). In general, **5** and **6** are able to discriminate the corresponding guest and no major differences between the resolution of aromatic and non-aromatic carboxylic acids were detected. Especially **5** gave excellent resolution for the non-aromatic carboxylic acids **13a**, **15a** and **16a**. According to the results, the best enantiomeric resolutions are obtained with racemic carboxylic acids bearing an electronegative atom (e.g., O, N, Br) at the α site. Therefore, it is interesting that compound **5** cannot discriminate the chiral proton in **12a**, nor does **6** discriminate the chiral proton in **12b**. This indicates that not only the polarity but also the size of the α-group (e.g., **14a,b**) and/or crowding at the α site (e.g., **3** and **4**) may have an effect. Intriguingly both **5** and **6** discriminate the prochiral CH_2_ protons of the *R* and *S* enantiomers of **14a** and **14b**. The resolution of carboxylic acids containing an electropositive (methyl group) at the α-site (**10** and **11**, **a** and **b**) was less efficient. In **14b** the CH_2_ peaks overlapped with those of **6**. This was overcome by using the 2D NMR techniques HSQC and NOESY (Supplementary Material Figures S17 and S18). Also other 2D experiments such as HMBC may be utilized among 1D experiments such as selective TOCSY as well as carbon selective 1D-HSQC when an overlap of CSA and substrate signals occurs. When the peaks are not baseline resolved (e.g., in case of **13a** and **14a**), certain specialised NMR techniques are available. Obviously, traditional line shape fitting of an ordinary 1D ^1^H spectrum can significantly enhance the accuracy of estimated ee values. Other possiblities for ee determination include pure shift experiments [38,39], *J*-resolved techniques [40] and the recently published RES-TOCSY [41]. Naturally, the use of 2D methods for reliable ee determination requires, that relevant, quantification-affecting NMR properties in the presence of chiral auxiliary for both enantiomers remain essentially similar (*cf.* Supplementary Material Figure S6). In the current study, the RES-TOCSY pulse sequence was modified to incorporate only one selective pulse (selective refocusing pulse) and gradients for coherence selection. The resulting 2D spectrum is phase sensitive, which allows the extraction of 1D traces with line shapes as encountered in the normal ^1^H spectrum. A more detailed description of the aforementioned pulse sequence as well as the actual pulse sequence code are given in Supplementary Material (Figures S15–S23). The use of this modified RES-TOCSY to enhance multiplet resolution in an indirectly detected dimension is demonstrated for compounds **13a** and **14a** in Figure 5.

**Table 3:** The magnitude of non-equivalences (Δδ) of five racemic carboxylic acids in the presence of **5** and their *n*-Bu_4_N salts in the presence of **6** (^1^H-NMR (500 MHz) in CDCl_3_ at 27 °C). The experiment was performed by adding a solution containing CSA (46.6 mM, 22.5 μL, 1.0 eq) to a solution of the guest (2.0 mM, 0.5 mL, 1.0 eq) (- indicates resolution was not detected).

Cmpd.	Racemic Carboxylic Acid		Δδ		Cmpd.	*n*-Bu_4_N Salt of Racemic Carboxylic Acid		Δδ	
			ppm	Hz				ppm	Hz
**10a**	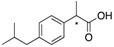	Me	0.0023	1.1	**10b**	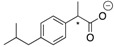	Me	-	-
		CH	-	-			CH	-	-
**11a**	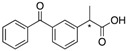	Me	0.0059	3.0	**11b**	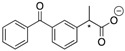	Me	0.0041	2.0
		CH	0.0047	2.4			CH	0.0021	1.0
**12a**	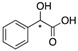	CH	-	-	**12b**	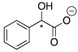	CH	-	-
**13a**	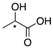	CH	0.0081	4.1	**13b**	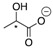	CH	0.0068	3.4
		Me	0.022	11.1			Me	0.017	8.6
**14a**	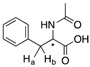	CH	0.012	6.1	**14b**	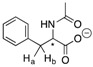	CH	0.012	6.0
		NH	0.013	6.5			NH	0.014	6.8
		Me	0.0028	1.4			Me	0.0039	1.9
		H_a_	0.072	36.2			H_a_^(a)^	0.065	32.5
		H_b_	0.027	13.4			H_b_^(a)^	0.036	17.8
**15a**	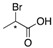	CH	-	-	**15a**	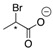	CH	-	-
		Me	0.023	11.5			Me	0.002	0.9
**16a**	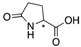	CH	0.016	7.9	**16b**	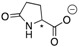	CH	0.003	1.4
		NH	0.020	10.2			NH	0.020	10.0

^(a)^ Due to overlapping with protons of [*n*-Bu_4_N]^+^ values are from HSQC/NOESY experiment.

## 3. Experimental Section

### 3.1. General Methods

All reagents and solvents were obtained from commercial suppliers and were used without further purification unless otherwise stated. (+)-Dehydroabietylamine was purchased (Sigma Aldrich, St. Louis, MO, USA) as 60% grade and purified using a method described in the literature [42] with slight modifications (see below). Flash chromatography was performed using 40–63 mesh silica gel. Microwave oven reactions were performed in closed vessel using the CEM Focused Microwave™ Synthesis System with an external infrared temperature control system (CEM, Matthews, NC, USA). NMR spectra were recorded using UNITY INOVA 500 (500 MHz ^1^H-frequency) and Mercury Plus 300 (300 MHz ^1^H-frequency) instruments (Varian, Palo Alto, CA, USA) at 27 °C. ^1^H-NMR spectra were recorded at 500 MHz, ^13^C-NMR spectra were recorded at 125 or 75 MHz, ^19^F-NMR spectra were recorded at 470 MHz. All 2D NMR experiments (NOESY, HSQC and RES-TOCSY) were recorded at 500 MHz (^1^H) and 125 (^13^C). Melting points were determined in a digital melting point apparatus (B 545, Büchi, Flawil, Switzerland). Optical rotations were determined with a digital polarimeter (DIP-1000, JASCO, Halifax, NS, Canada) at 22–23 °C in CHCl_3_ or MeOH as solvent. The exact mass was obtained using high-resolution mass spectrometry (MicroTOF LC, Bruker, Billerica, MA, USA) with electrospray ionisation (ESI).

### 3.2. Synthesis of Chiral Solvating Agents

#### 3.2.1. Purification of (+)-Dehydroabietylamine (**2**) 

Acetic acid (9.65 g) in toluene (30.0 mL) was slowly added to 60% (+)-dehydroabietylamine (42.0 g) in toluene (70.0 mL). The product (+)-dehydroabietylaminium acetate was left to crystallise in a fridge, filtered, washed with hexane and recrystallised from MeOH. The salt (21.0 g) was dissolved in hot water and 10% NaOH solution (28.0 mL) was added. (+)-Dehydroabietylamine was extracted by Et_2_O and the organic phase was washed with water until neutral. The organic phase was dried over Na_2_SO_4_. The solvent was evaporated and (+)-dehydroabietylamine was dried under vacuum; mp 44.2 °C (lit. 44–45 °C) [43]; HRMS-ESI (*m/z*) calc. for C_20_H_32_N [M + H]^+^ 286.2529 found 286.2540; ${\text{[α]}}_{\text{D}}^{22}$ +44.35 (*c* 1.0, CHCl_3_) (lit. +58.0, *c* 0.2, DMSO, 20 °C) [43]; ^1^H-NMR (500 MHz, CDCl_3_) δ ppm 0.85 (s, 3H, C*H*_3_), 1.22 (s, 3H, C*H*_3_), 1.22 (d, *J* = 6.98 Hz, 6H, 2× C*H*_3_), 1.33 (m, 2H, C*H*_2_), 1.39 (m, 1H, C*H*H), 1.52 (dd, *J* = −11.75, 3.31 Hz, 1H, C*H*), 1.69 (m, 2H, C*H*_2_), 1.74 (m, 2H, C*H*_2_), 2.29 (dt, *J* = −13.14, 1.72 Hz, 1H, CH*H*), 2.40 (d, *J* = −13.46 Hz, 1H, C*H*H), 2.61 (d, *J* = −13.46 Hz, 1H, CH*H*), 2.82 (sep, *J* = 6.98 Hz, 1H, C*H*), 2.88 (m, 2H, C*H*_2_), 6.89 (d, *J* = 1.94 Hz, 1H, C*H*_Ar_), 6.99 (dd, *J* = 8.08, 1.94 Hz, 1H, C*H*_Ar_), 7.18 (d, *J* = 8.08 Hz, 1H, C*H*_Ar_); ^13^C-NMR (125 MHz, CDCl_3_) δ ppm 18.8 (*C*H_2_), 18.9 (*C*H_3_), 18.895 (*C*H*_2_*), 24.1 H_3_), 24.130 (*C*H_3_), 25.4 (*C*H_3_), 30.3 (*C*H_2_), 33.6 (*C*H), 35.355 (*C*H_2_), 37.4 (*C*), 37.527 (*C*), 38.7 (*C*H_2_), 45.0 (*C*H), 53.9 (*C*H_2_), 123.9 (*C*H_Ar_), 124.4 (*C*H_Ar_), 126.9 (*C*H_Ar_), 134.8 (*C*_Ar_), 145.7 (*C*_Ar_), 147.6 (*C*_Ar_).

#### 3.2.2. Synthesis of (+)-Dehydroabietyl-*N,N*-dimethylmethanamine (**5**)

Formic acid (3.96 mL, 0.105 mol, 5.0 eq) was added to **2** (6.0 g, 0.021 mol, 1.0 eq) at 0 °C. Formaldehyde (37% H_2_O solution, 3.81 mL, 0.048 mol, 2.3 eq) was added dropwise and the reaction mixture was refluxed for 4 h. The reaction mixture was cooled to rt. and made basic by adding NaOH solution (2.0 M). The product was extracted with diethyl ether. The organic phase was washed with water until neutral, dried over Na_2_SO_4_, concentrated and dried in vacuum. The product was purified by column chromatography (2:3 DCM:EtOAc). Colorless liquid (4.2 g 63.8%); HRMS-ESI (*m/z*) calc. for C_22_H_36_N [M + H]^+^ 314.2842, found 314.2840; ${\text{[α]}}_{\text{D}}^{22}$ +52.05 (*c* 1.0, CHCl_3_); ^1^H-NMR (500 MHz, CDCl_3_) δ ppm 0.85 (s, 3H, C*H*_3_), 1.22 (s, 3H, C*H*_3_), 1.23 (d, *J* = 6.91 Hz, 6H, 2× C*H*_3_), 1.34 (m, 1H, C*H*H), 1.43 (td, *J* = −13.21, 4.15 Hz, 1H, C*H*H), 1.56 (td, *J* = −13.21, 4.15 Hz, 1H, CH*H*), 1.65 (m, 2H, C*H*_2_), 1.701 (m, 1H, C*H*), 1.704 (m, 1H, C*H*H), 1.79 (m, 1H, CH*H*), 1.95 (d, *J* = −14.25 Hz, 1H, C*H*H), 2.26 (m, 1H, CH*H*), 2.28 (s, 6H, 2× C*H*_3_), 2.29 (d, *J* = −14.25 Hz, 1H, CH*H*), 2.83 (sep, *J*= 6.91 Hz, 1H, C*H*), 2.88 (m, 2H, C*H*_2_), 6.88 (d, *J* = 1.90 Hz, 1H, C*H*_Ar_), 6.98 (dd, *J* = 8.14, 1.90 Hz, 1H, C*H*_Ar_), 7.18 (d, *J* = 8.14 Hz, 1H, C*H*_Ar_); ^13^C-NMR (75 MHz, CDCl_3_) δ ppm 19.0 (*C*H_3_), 19.1 (*C*H_2_), 19.3 (*C*H_2_), 24.2 (2× *C*H_3_), 25.8 (*C*H_3_), 30.3 (*C*H_2_), 33.6 (*C*H), 36.6 (*C*H_2_), 37.6 (*C*), 38.6 (*C*H_2_), 38.9 (*C*), 44.3 (*C*H), 49.3 (2× *C*H_3_), 71.2 (*C*H_2_), 123.8 (*C*H_A*r*_), 124.3 (*C*H_Ar_), 126.9 (*C*H_Ar_), 134.9 (*C*_Ar_), 145.5 (*C*_Ar_), 147.9 (*C*_Ar_).

#### 3.2.3. Synthesis of (+)-Dehydroabietyl-*N*,*N*-dimethylmethanaminium bis(trifluoromethanesulfon-imide) (**6**)

HNTf_2_ (89.7 mg, 1.0 eq) was added at 0 °C to the tertiary amine **5** (0.1 g, 0.319 mmol, 1.0 eq) in DCM (0.5 mL). After stirring for 1 h at rt water was added. The layers were separated and the organic phase washed with water (3 × 2.0 mL). The organic solvent was evaporated and the product dried under vacuum. White solid (0.18 g, 93.3%;); mp 114.2 °C; HRMS-ESI (*m/z*) calc. for C_22_H_36_N [M − NTf_2_]^+^314.2842, found 314.2834; calc. for C_2_F_6_NO_4_S_2_ [NTf_2_] 279.9167, found 279.9170; ${\text{[α]}}_{\text{D}}^{23}$ +7.45 (*c* 1.0, CHCl_3_); ^1^H-NMR (500 MHz, CDCl_3_) δ ppm 1.16 (s, 3H, C*H*_3_), 1.21 (d, *J* = 6.88 Hz, 6H, 2× C*H*_3_), 1.24 (s, 3H, C*H*_3_), 1.33 (m, 1H, C*H*H), 1.37 (dd, *J* = −12.67, 2.07 Hz, 1H, C*H*), 1.43 (m, 1H, C*H*H), 1.65 (dt, *J* = −13.45, 7.69 Hz, 1H, C*H*H), 1.67 (dt, *J* = −13.45, 7.69 Hz, 1H, CH*H*), 1.79 (m, 2H,C*H*_2_), 1.84 (m, 1H, CH*H*), 2.34 (dt, *J* = −13.27, 2.97 Hz, 1H, CH*H*), 2.82 (sep, *J* = 6.88 Hz, 1H, C*H*), 2.85 (m, 1H, C*H*H), 2.94 (d, *J* = −13.22 Hz, 1H, C*H*H), 2.95 (m, 1H, CH*H*), 3.01 (s, 6H, 2× C*H*_3_), 3.28 (d, *J* = −13.22 Hz, 1H,CH*H*), 6.88 (d, *J* = 1.65 Hz, 1H, C*H*_Ar_), 6.99 (dd, *J* = 8.14, 1.65 Hz, 1H, C*H*_Ar_), 7.14 (d, *J* = 8.14 Hz, 1H, C*H*_Ar_); ^13^C-NMR (125 MHz, CDCl_3_) δ ppm 17.6 (*C*H_3_), 18.1 (*C*H_2_), 19.5 (*C*H_2_), 24.1 (2× *C*H_3_), 25.4 (*C*H_3_), 29.7 (*C*H_2_), 33.6 (*C*H), 35.9 (*C*H_2_), 37.7 (*C*), 37.7 (*C*H_2_), 37.7(*C*), 47.4 (*C*H), 48.4 (2× *C*H_3_), 73.3 (*C*H_2_), 119.8 (q, *J* = 319.79, *C*F_3_), 124.1 (*C*H_A*r*_), 124.4 (*C*H_Ar_), 126.9 (*C*H_Ar_), 133.7 (*C*_Ar_), 146.2 (*C*_Ar_), 146.3 (*C*_Ar_).

#### 3.2.4. Synthesis of (+)-Dehydroabietyl-*N*,*N*,*N*-trimethylmethanaminium iodide (**7a**) 

The tertiary amine **5** (0.15 g, 0.479 mmol, 1.0 eq), iodomethane (0.68 g 0.30 mL, 4.79 mmol, 10.0 eq, caution toxic) and CHCl_3_ (1.0 mL) were measured into a microwave tube. The reaction was irradiated in microwave oven at 120 W, 30 min, 47 °C. Et_2_O was added and the product was collected by filtration and washed with Et_2_O. The product was dried in vacuum. White solid (0.18 g, 82.9%); mp 250.3 °C (lit. 249–252 °C) [44]; HRMS-ESI (*m/z*) calc. for C_23_H_38_N [M − I]^+^ 328.2999, found 328.2988; ${\text{[α]}}_{\text{D}}^{23}$ = +20.91 (*c* 1.0, CHCl_3_) (lit. +22.0, *c* 1.20, CHCl_3_, 20 °C) [44]; ^1^H-NMR (500 MHz, CDCl_3_) δ ppm 1.19 (d, *J* = 7.01 Hz, 6H, 2× C*H*_3_), 1.23 (s, 3H, C*H*_3_), 1.33 (s, 3H, C*H*_3_), 1.40 (dd, *J* = −12.32, 1.87 Hz, 1H, C*H*), 1.42 (m, 1H, C*H*H), 1.73 (m, 1H, C*H*H), 1.74 (m, 2H, C*H*_2_), 1.86 (m, 1H, C*H*H), 2.00 (m, 1H, CH*H*), 2.05 (m, 1H, CH*H*), 2.30 (dt, *J* = −13.03 Hz, 1H, CH*H*), 2.80 (sep, *J* = 7.01 Hz, 1H, C*H*), 2.94 (m, 2H, C*H*_2_), 3.39 (d, *J* = −14.04 Hz, 1H, C*H*H), 3.57 (s, 9H, 3× C*H*_3_), 3.94 (d, *J* = −14.04 Hz, 1H, CH*H*), 6.88 (d, *J* = 1.75 Hz, 1H, C*H*_Ar_), 6.96 (dd, *J* = 8.20, 1.75 Hz, 1H, C*H*_Ar_), 7.09 (d, *J* = 8.20 Hz, 1H, C*H*_Ar_); ^13^C-NMR (125 MHz, CDCl_3_) δ ppm 18.6 (*C*H_2_), 20.06 (*C*H_3_), 20.08 (*C*H_2_), 24.18 (*C*H_3_), 24.22 (*C*H_3_), 25.8 (*C*H_3_), 29.9 (*C*H_2_), 33.7 (*C*H), 37.7 (*C*H_2_), 38.2 (*C*), 39.3 (*C*H_2_), 40.9 (*C*), 48.1 (*C*H), 56.9 (3× *C*H_3_), 78.7 (*C*H_2_), 123.8 (*C*H_Ar_), 124.2 (*C*H_Ar_), 127.2 (*C*H_Ar_), 134.4 (*C*_Ar_), 146.3 (*C*_Ar_), 146.8 (*C*_Ar_).

#### 3.2.5. Synthesis of *N*-Benzyl-1-(+)-dehydroabietyl-*N*,*N*-dimethylmethanaminium bromide (**8a**) 

The tertiary amine **1** (0.3 g, 0.956 mmol, 1.0 eq) and benzyl bromide (0.491 g, 0.341 mL, 2.87 mmol, l.3 eq) were measured into a microwave tube and EtOAc (1.0 mL) was added. The reaction was irradiated in the oven at 170 W, 13 min, 80 °C. The crystalline solid formed was filtered and washed with EtOAc. White solid (0.27 g, 59.4%;); mp 188.6 °C (lit. 225–231 °C) [44]; HRMS-ESI (*m/z*) calc. for C_29_H_42_N [M − Br]^+^ 404.3312, found 404.3310; ${\text{[α]}}_{\text{D}}^{23}$ +7.19 (*c* 1.0, CHCl_3_) (lit. +11.0, *c* 1.07, CHCl_3_, 20 °C) [44]; ^1^H-NMR (500 MHz, CDCl_3_) δ ppm 1.20 (d, *J* = 7.00 Hz, 6H, 2× C*H*_3_), 1.21 (s, 3H, C*H*_3_), 1.32 (m, 1H, C*H*), 1.33 (s, 3H, C*H*_3_), 1.34 (m, 1H, C*H*H), 1.59 (m, 1H, C*H*H), 1.70 (m, 2H, C*H*_2_), 1.82 (m, 1H, C*H*H), 1.99 (m, 1H, CH*H*), 1.99 (m, 1H, CH*H*), 2.26 (dt, *J* = −12.88, 3.00 Hz, 1H, CH*H*), 2.80 (sep, *J* = 7.00 Hz, 1H, C*H*), 2.85 (m, 1H, C*H*H), 2.93 (m, 1H, CH*H*), 3.32 (s, 3H, C*H*_3_), 3.33 (d, *J* = −14.11 Hz, 1H, C*H*H), 3.42 (s, 3H, C*H*_3_), 4.08 (d, *J* = −14.11 Hz, 1H, CH*H*), 5.23 (d, *J* = −12.44 Hz, 1H, C*H*H), 5.33 (d, *J* = −12.44 Hz, 1H, CH*H*), 6.86 (d, *J* = 1.59 Hz, 1H, C*H*_Ar_), 6.96 (dd, *J* = 8.23, 1.59 Hz, 1H, C*H*_Ar_), 7.07 (d, *J* = 8.23 Hz, 1H, C*H*_Ar_), 7.37 (m, 1H, C*H*_Ar_), 7.40 (m, 1H, C*H*_Ar_), 7.70 (d, *J* = 7.14 Hz, 1H, C*H*_Ar_); ^13^C-NMR (125 MHz, CDCl_3_) δ ppm 18.5 (*C*H_2_), 19.9 (*C*H_2_), 20.1 (*C*H_3_), 24.0 (*C*H_3_), 24.1 (*C*H_3_), 25.6 (*C*H_3_), 29.8 (*C*H_2_), 33.5 (*C*H), 37.6 (*C*H_2_), 38.1 (*C*), 39.6 (*C*H_2_), 40.6 (*C*), 48.4 (*C*H), 51.4 (*C*H_3_), 51.6 (*C*H_3_), 71.9 (*C*H_2_), 75.9 (*C*H_2_), 123.7 (*C*H_Ar_), 124.1 (*C*H_Ar_), 127.0 (*C*H_Ar_), 127.7 (*C*_Ar_), 129.2 (*C*H_Ar_), 130.8 (*C*H_Ar_), 133.8 (*C*H_Ar_), 134.3 (*C*_Ar_), 146.1 (*C*_Ar_), 146.8 (*C*_Ar_).

#### 3.2.6. Synthesis of 2-Hydroxy-*N*-(+)-dehydroabietyl-*N*,*N*-dimethylethanaminium bromide (**9a**) 

The tertiary amine **5** (0.3 g, 0.956 mmol, 1.0 eq) and benzyl bromide (0.359 g, 0.204 mL, 2.87 mmol, 3.0 eq) were measured into a microwave tube and CHCl_3_ was added. The mixture was irradiated in the oven at 120 W, 2 h, 110 °C. The solvent was evaporated and product washed with Et_2_O (3 mL × 3). The product was purified by column chromatography (1:9 MeOH:DCM). White powder (0.20 g, 46.5%); mp 213.5 °C; HRMS-ESI (*m/z*) calc. for C_24_H_40_NO [M − Br]^+^ 358.3104, found 358.3095; ${\text{[α]}}_{\text{D}}^{23}$ +16.45 (*c* 1.0, MeOH); ^1^H-NMR (500 MHz, CD_3_OD) δ ppm 1.71 (d, *J* = 6.91 Hz, 6H, 2× C*H*_3_), 1.79 (s, 3H, C*H*_3_), 1.88 (s, 3H, C*H*_3_), 1.97 (m, 1H, C*H*H), 1.99 (dd, *J* = −11.85, 2.49 Hz, 1H, C*H*), 2.25 (m, 1H, C*H*H), 2.31 (m, 1H, C*H*H), 2.37 (m, 1H, CH*H*), 2.40 (m, 1H, C*H*H), 2.47 (m, 1H, CH*H*), 2.63 (dt, *J* = −11.93 Hz, 1H, CH*H*), 2.89 (dt, *J* = −12.68 Hz, 1H, CH*H*), 3.31 (sep, *J* = 6.91 Hz, 1H, C*H*), 3.43 (m, 1H, C*H*H), 3.48 (ddd, *J* = −17.37, 7.17, 2.13 Hz, 1H, CH*H*), 3.83 (s, 6H, 2× C*H*_3_), 3.91 (d, *J* = −14.01 Hz, 1H, C*H*H), 4.16 (m, 2H, C*H*_2_), 4.27 (d, *J* = −14.01 Hz, 1H, CH*H*), 4.56 (m, 2H, C*H*_2_), 7.39 (d, *J* = 1.94 Hz, 1H, C*H*_Ar_), 7.48 (dd, *J* = 8.27, 1.94 Hz, 1H, *CH*_Ar_), 7.66 (d, *J* = 8.27 Hz, 1H, *CH*_Ar_); ^13^C-NMR (125 MHz, CD_3_OD) δ ppm 19.5 (*C*H_2_), 19.9 (*C*H_3_), 20.6 (*C*H_2_), 24.5 (2× *C*H_3_), 25.9 (*C*H_3_), 30.8 (*C*H_2_), 34.8 (*C*H), 38.9 (*C*), 39.2 (*C*H_2_), 39.7 (*C*H_2_), 41.6 (*C*), 49.6 (*C*H), 54.3 (*C*H_3_), 54.6 (*C*H_3_), 57.1 (*C*H_2_), 70.3 (*C*H_2_), 79.0 (*C*H_2_), 124.7 (*C*H_Ar_), 124.9 (*C*H_Ar_), 127.7 (*C*H_Ar_), 135.3 (*C*_Ar_), 147.2 (*C*_Ar_), 148.3 (*C*_Ar_).

#### 3.2.7. General Procedure for Anion Exchange 

Anion exchange reactions were performed as described in the literature [32]. LiNTf_2_ or NH_4_BF_4_ solution (1.0 M in H_2_O, 1.0 eq) was added to CSA (1.0 eq, in DCM) at rt. and stirred for 1 h. Phases were separated and the organic phase was washed with water, concentrated and dried in vacuum.

*(+)-Dehydroabietyl-N,N,N-trimethylmethanaminium tetrafluoroborate* (**7b**). White solid (0.085 g, 93.3%); mp 246.8 °C; HRMS-ESI (*m/z*) calc. for C_23_H_38_N [M – BF_4_]^+^ 328.2999, found 328.2990; ${\text{[α]}}_{\text{D}}^{23}$ = +19.93 (*c* 1.0, CHCl_3_ + 3% MeOH); ^1^H-NMR (500 MHz, CDCl_3_ + 3% CD_3_OD) δ ppm 1.21 (d, *J* = 7.02 Hz, 6H, 2× C*H*_3_), 1.25 (s, 3H, C*H*_3_), 1.31 (s, 3H, C*H*_3_), 1.40 (dd, *J* = −11.63, 2.60 Hz, 1H, C*H*), 1.43 (m, 1H, C*H*H), 1.64 (m, 1H, C*H*H), 1.76 (m, 2H, C*H*_2_), 1.89 (m, 2H, C*H*_2_), 2.00 (dt, *J* = −13.10, 2.87 Hz, 1H, CH*H*), 2.32 (dt, *J* = −12.83, 2.60 Hz, 1H, CH*H*), 2.81 (sep, *J* = 7.03 Hz, 1H, C*H*), 2.91 (m, 1H, C*H*H), 2.96 (ddd, *J* = −17.17, 7.06, 1.86 Hz, 1H, CH*H*), 3.22 (d, *J* = −14.33 Hz, 1H, C*H*H), 3.34 (s, 9H, 3× C*H*_3_), 3.67 (d, *J* = −14.33 Hz, 1H, CH*H*), 6.89 (d, *J* = 2.23 Hz, 1H, C*H*_Ar_), 6.98 (dd, *J* = 8.22, 2.23 Hz, 1H, C*H*_Ar_), 7.11 (d, *J* = 8.22 Hz, 1H, C*H*_Ar_); ^13^C-NMR (125 MHz, CDCl_3_, +3% CD_3_OD) δ ppm 18.4 (*C*H_2_), 19.5 (*C*H_3_), 19.6 (*C*H_2_), 23.9 (*C*H_3_), 24.0 (*C*H_3_), 25.6 (*C*H_3_), 29.6 (*C*H_2_), 33.5 (*C*H), 37.6 (*C*H_2_), 38.0 (*C*), 38.8 (*C*H_2_), 40.6 (*C*), 47.9 (*C*H), 56.5 (3× *C*H_3_), 78.8 (*C*H_2_), 123.6 (*C*H_Ar_), 124.1 (*C*H_Ar_), 126.9 (*C*H_Ar_), 134.1 (*C*_Ar_), 146.1 (*C*_Ar_), 146.6 (*C*_Ar_).

*(+)-Dehydroabietyl-N,N,N-trimethylmethanaminium bis(trifluoromethanesulfonimide)* (**7c**). White solid (0.13 g, 97.3%); mp 46.6 °C; HRMS-ESI (*m/z*) calc. for C_23_H_38_N [M − NTf_2_]^+^ 328.2999, found 328.3011; calc. for C_2_F_6_NO_4_S_2_ [NTf_2_] 279.9167, found 279.9169; ${\text{[α]}}_{\text{D}}^{23}$ = +7.79 (*c* 1.0, CHCl_3_); ^1^H-NMR (500 MHz, CDCl_3_) δ ppm 1.21 (d, *J* = 7.04 Hz, 6H, 2× C*H*_3_), 1.26 (s, 3H, C*H*_3_), 1.31 (s, 3H, C*H*_3_), 1.39 (dd, *J* = −12.30, 2.70 Hz, 1H, C*H*), 1.42 (m, 1H, C*H*H), 1.60 (m, 1H, C*H*H), 1.76 (m, 2H, C*H*_2_), 1.83 (m, 2H, C*H*_2_), 1.99 (m, 1H, CH*H*), 2.33 (dt, *J* = −13.31, 3.12 Hz, 1H, CH*H*), 2.82 (sep, *J* = 7.04 Hz, 1H, C*H*), 2.87 (m, 1H, C*H*H), 2.98 (dd, *J* = −17.20, 6.70 Hz, 1H, CH*H*), 3.11 (d, *J* = −14.21 Hz, 1H, C*H*H), 3.29 (s, 9H, 3× C*H*_3_), 3.53 (d, *J* = −14.21 Hz, 1H, CH*H*), 6.89 (d, *J* = 1.88 Hz, 1H, C*H*_Ar_), 6.99 (dd, *J* = 8.21, 1.88 Hz, 1H, C*H*_Ar_), 7.11 (d, *J* = 8.21 Hz, 1H, C*H*_Ar_); ^13^C-NMR (125 MHz, CDCl_3_) δ ppm 18.4 (*C*H_2_), 19.4 (*C*H_3_), 19.6 (*C*H_2_), 24.0 (*C*H_3_), 24.1 (*C*H_3_), 25.6 (*C*H_3_), 29.5 (*C*H_2_), 33.6 (*C*H), 37.6 (*C*H_2_), 38.1 (*C*), 38.9 (*C*H_2_), 40.7 (*C*), 47.9 (*C*H), 56.7 (3× *C*H_3_), 79.3 (*C*H_2_), 119.9 (q, *J* = 320.54, *C*F_3_), 123.7 (*C*H_Ar_), 124.2 (*C*H_Ar_), 127.1 (*C*H_Ar_), 134.0 (*C*_Ar_), 146.3 (*C*_Ar_), 146.5 (*C*_Ar_).

*N-Benzyl-1-(+)-dehydroabietyl-N,N-dimethylmethanaminium tetrafluoroborate* (**8b**). White solid (0.19 g, 92.6%); mp 179.9 °C; HRMS-ESI (*m/z*) calc. for C_29_H_42_N [M − BF_4_]^+^ 404.3312, found 404.3311; ${\text{[α]}}_{\text{D}}^{23}$ = +2.28 (*c* 1.0, CHCl_3_); ^1^H-NMR (500 MHz, CDCl_3_) δ ppm 1.206 (d, *J* = 6.86 Hz, 6H, 2× C*H*_3_), 1.209 (s, 3H, C*H*_3_), 1.29 (s, 3H, C*H*_3_), 1.33 (m, 1H, C*H*), 1.39 (m, 1H, C*H*H), 1.54 (m, 1H, C*H*H), 1.70 (m, 2H, C*H*_2_), 1.82 (m, 2H, C*H*_2_), 1.98 (dt, *J* = −12.28 Hz, 1H, CH*H*), 2.28 (dt, *J* = −12.77 Hz, 1H, CH*H*), 2.81 (sep, *J* = 6.86 Hz, 1H, C*H*), 2.83 (m, 1H, C*H*H), 2.92 (m, 1H, CH*H*), 3.14 (s, 3H, C*H*_3_), 3.17 (d, *J* = −12.75 Hz, 1H, C*H*H), 3.21 (s, 3H, C*H*_3_), 3.71 (d, *J* = −12.75 Hz, 1H, CH*H*), 4.69 (d, *J* = −12.75 Hz, 1H, C*H*H), 4.73 (d, *J* = −12.75 Hz, 1H, CH*H*), 6.86 (d, *J* = 1.87 Hz, 1H, C*H*_Ar_), 6.97 (dd, *J* = 8.17, 1.87 Hz, 1H, C*H*_Ar_), 7.08 (d, *J* = 8.17 Hz, 1H, C*H*_Ar_), 7.39 (m, 1H, C*H*_Ar_), 7.42 (m, 1H, C*H*_Ar_), 7.55 (m, 1H, C*H*_Ar_); ^13^C-NMR (75 MHz, CDCl_3_) δ ppm 18.5 (*C*H_2_), 19.7 (*C*H_2_), 19.8 (*C*H_3_), 24.08 (*C*H_3_), 24.11 (*C*H_3_), 25.6 (*C*H_3_), 29.8 (*C*H_2_), 33.6 (*C*H), 37.6 (*C*H_2_), 38.1 (*C*), 39.2 (*C*H_2_), 40.6 (*C*), 48.4 (*C*H), 51.3 (*C*H_3_), 51.6 (*C*H_3_), 73.1 (*C*H_2_), 76.2 (*C*H_2_), 123.7 (*C*H_Ar_), 124.1 (*C*H_Ar_), 127.0 (*C*H_Ar_), 127.4 (*C*_Ar_), 129.3 (*C*H_Ar_), 130.9 (*C*H_Ar_), 133.6 (*C*H_Ar_), 134.3 (*C*_Ar_), 146.2 (*C*_Ar_), 146.8 (*C*_Ar_).

*N-Benzyl-1-(+)-dehydroabietyl-N,N-dimethylmethanaminium bis(trifluoromethanesulfonimide)* (**8c**). Colorless glass (0.27 g, 89.9%); mp 48.8 °C; HRMS-ESI (*m/z*) calc. for C_29_H_42_N [M − NTf_2_]^+^ 404.3312 found 404.3323 calc. for C_2_F_6_NO_4_S_2_ [NTf_2_] 279.9167, found 279.9166; ${\text{[α]}}_{\text{D}}^{23}$ = +1.70 (*c* 1.0, CHCl_3_); ^1^H-NMR (500 MHz, CDCl_3_) δ ppm 1.22 (d, *J* = 6.93 Hz, 6H, 2× C*H*_3_), 1.24 (s, 3H, C*H*_3_), 1.31 (s, 3H, C*H*_3_), 1.37 (m, 1H, C*H*), 1.41 (m, 1H, C*H*H), 1.53 (m, 1H, C*H*H), 1.74 (m, 2H, C*H*_2_), 1.84 (m, 2H, C*H*_2_), 2.00 (dt, *J* = −12.37, 3.29 Hz, 1H, CH*H*), 2.31 (dt, *J* = −12.83, 2.82 Hz, 1H, CH*H*), 2.82 (m, 1H, C*H*H), 2.83 (sep, *J* = 6.93 Hz, 1H, C*H*), 2.97 (dd, *J* = −17.75, 6.68 Hz, 1H, CH*H*), 3.10 (d, *J* = −14.00 Hz, 1H, C*H*H), 3.11 (s, 3H, C*H*_3_), 3.17 (s, 3H, C*H*_3_), 3.59 (d, *J* = −14.00 Hz, 1H, CH*H*), 4.50 (d, *J* = −12.54 Hz, 1H, C*H*H), 4.56 (d, *J* = −12.54 Hz, 1H, CH*H*), 6.89 (d, *J* = 2.05 Hz, 1H, *C*H_Ar_), 6.99 (dd, *J* = 8.16, 2.05 Hz, 1H, *C*H_Ar_), 7.11 (d, *J* = 8.16 Hz, 1H, *C*H_Ar_), 7.469 (m, 1H, *C*H_Ar_), 7.472 (m, 1H, *C*H_Ar_), 7.51 (m, 1H, *C*H_Ar_); ^13^C-NMR (75 MHz, CDCl_3_) δ ppm 18.4 (*C*H_2_), 19.7 (*C*H_2_), 19.8 (*C*H_3_), 24.06 (*C*H_3_), 24.09 (*C*H_3_), 25.6 (*C*H_3_), 29.6 (*C*H_2_), 33.6 (*C*H), 37.6 (*C*H_2_), 38.2 (*C*), 39.3 (*C*H_2_), 40.8 (*C*), 48.3 (*C*H), 51.6 (*C*H_3_), 51.9 (*C*H_3_), 73.6 (*C*H_2_), 76.8 (*C*H_2_), 119.9 (q, *J* = 320.36, *C*F_3_), 123.7 (*C*H_Ar_), 124.3 (*C*H_Ar_), 126.7 (*C*_Ar_), 127.0 (*C*H_Ar_), 129.6 (*C*H_Ar_), 131.4 (*C*H_Ar_), 133.3 (*C*H_Ar_), 134.0 (*C*_Ar_), 146.3 (*C*_Ar_), 146.6 (*C*_Ar_).

*2-Hydroxy-N-(+)-dehydroabietyl-N,N-dimethylethanaminium tetrafluoroborate* (**9b**). White solid (0.093 g, 91.3%); mp 188.5 °C; HRMS-ESI (*m/z*) calc. for C_24_H_40_NO [M − BF_4_]^+^ 358.3104, found 358.3117; ${\text{[α]}}_{\text{D}}^{23}$ = +18.73 (*c* 1.0, CHCl_3_); ^1^H-NMR (500 MHz, CDCl_3_) δ ppm 1.20 (d, *J* = 6.93 Hz, 6H, 2× C*H*_3_), 1.24 (s, 3H, C*H*_3_), 1.30 (s, 3H, C*H*_3_), 1.38 (m, 1H, C*H*), 1.42 (m, 1H, C*H*H), 1.60 (m, 1H, C*H*H), 1.73 (m, 2H, C*H*_2_), 1.85 (m, 2H, C*H*_2_), 2.01 (dt, *J* = −12.20 Hz, 1H, CH*H*), 2.29 (dt, *J* = −13.03 Hz, 1H, CH*H*), 2.80 (sep, *J* = 6.93 Hz, 1H, C*H*), 2.93 (m, 2H, C*H*_2_), 3.18 (d, *J* = −14.31 Hz, 1H, C*H*H), 3.24 (s, 3H, C*H*_3_), 3.25 (s, 3H, C*H*_3_), 3.60 (br s, 2H, C*H*_2_), 3.64 (d, *J* = −14.31 Hz, 1H, CH*H*), 4.07 (br s, 2H, C*H*_2_), 6.88 (d, *J* = 1.75 Hz, 1H, C*H*_Ar_), 6.97 (dd, *J* = 8.11, 1.75 Hz, 1H, C*H*_Ar_), 7.09 (d, *J* = 8.11 Hz, 1H, C*H*_Ar_); ^13^C-NMR (75 MHz, CDCl_3_) δ ppm 18.5 (*C*H_2_), 19.62 (*C*H_2_), 19.64 (*C*H_3_), 24.08 (*C*H_3_), 24.11 (*C*H_3_), 25.7 (*C*H_3_), 29.7 (*C*H_2_), 33.6 (*C*H), 37.7 (*C*), 38.2 (*C*H_2_), 39.1 (*C*H_2_), 40.7 (*C*), 48.2 (*C*H), 53.7 (*C*H_3_), 53.9 (*C*H_3_), 56.8 (*C*H_2_), 69.3 (*C*H_2_), 78.3 (*C*H_2_), 123.7 (*C*H_Ar_), 124.1 (*C*H_Ar_), 127.0 (*CH*_Ar_), 134.3 (*C*_Ar_), 146.2 (*C*_Ar_), 146.8 (*C*_Ar_).

*2-Hydroxy-N-(+)-dehydroabietyl-N,N-dimethylethanaminium bis(trifluoromethanesulfonimide)* (**9c**). Viscous colorless liquid (0.14 g, 97.8%); HRMS-ESI (*m/z*) Calc. for C_24_H_40_NO [M − NTf_2_]^+^ 358.3104, found 358.3105; calc. for C_2_F_6_NO_4_S_2_ [NTf_2_] 279.9167, found 279.9164; ${\text{[α]}}_{\text{D}}^{23}$ = +6.56 (c 1.0, CHCl_3_); ^1^H-NMR (500 MHz, CDCl_3_) δ ppm 1.21 (d, *J* = 6.80 Hz, 6H, 2× C*H*_3_), 1.26 (s, 3H, C*H*_3_), 1.31 (s, 3H, C*H*_3_), 1.39 (m, 1H, C*H*), 1.43 (m, 1H, C*H*H), 1.59 (m, 1H, C*H*H), 1.82 (m, 2H, C*H*_2_), 2.01 (dt, *J* = −12.52, 2.59 Hz, 1H, CH*H*), 2.33 (dt, *J* = −12.93, 2.99 Hz, 1H, CH*H*), 2.82 (sep, *J* = 6.80 Hz, 1H, C*H*), 2.87 (m, 1H, C*H*H), 2.97 (ddd, *J* = −17.65, 6.80, 1.81 Hz, 1H, CH*H*), 3.18 (d, *J* = −14.05 Hz, 1H, C*H*H), 3.26 (s, 3H, C*H*_3_), 3.27 (s, 3H, C*H*_3_), 3.61 (br s, 2H, C*H*_2_), 3.62 (d, *J* = −14.05 Hz, 1H, CH*H*), 4.09 (br s, 2H, C*H*_2_), 6.89 (d, *J* = 1.87 Hz, 1H, C*H*_Ar_), 6.99 (dd, *J* = 8.15, 1.87 Hz, 1H, C*H*_Ar_), 7.11 (d, *J* = 8.15 Hz, 1H, C*H*_Ar_); ^13^C-NMR (75 MHz, CDCl_3_) δ ppm 18.4 (*C*H_2_), 19.66 (*C*H_2_), 19.70 (*C*H_3_), 24.05 (*C*H_3_), 24.11 (*C*H_3_), 25.6 (*C*H_3_), 29.5 (*C*H_2_), 33.6 (*C*H), 37.6 (*C*H_2_), 38.2 (*C*), 39.3 (*C*H_2_), 40.8 (*C*), 48.2 (*C*H), 54.0 (*C*H_3_), 54.2 (*C*H_3_), 56.8 (*C*H_2_), 69.6 (*C*H_2_), 78.7 (*C*H_2_), 119.9 (q, *J* = 320.27, *C*F_3_), 123.7 (*C*H_Ar_), 124.3 (*C*H_Ar_), 127.0 (*C*H_Ar_), 134.0 (*C*_Ar_), 146.3 (*C*_Ar_), 146.6 (*C*_Ar_).

#### 3.2.8. Synthesis of Guests

*N*-Acetylation of phenylalanine was performed according to the literature [45]. *n*-Bu_4_N salts were prepared by adding tetrabutylammonium hydroxide (1.0 M in MeOH, 1.0 eq) to the racemic acid (1.0 eq) in MeOH. After stirring for 1–3 h, the solvent was evaporated and the product was dried under vacuum.

## 4. Conclusions

A number of new chiral tertiary and quaternary amine solvating agents (CSAs) based on (+)-dehydroabietylamine were synthesized and found to resolve racemic Mosher’s acid and its *n*-Bu_4_N salt by ^1^H-NMR and ^19^F-NMR spectrocopy. Optimum conditions for the enantiomeric resolution with the CSAs were determined by titration. The CSAs are also useful in the determination of ee values of enantiomerically enriched mixtures. Both non-ionic and ionic CSAs resolve racemic carboxylic acids equally well. Best results were obtained with racemic carboxylic acids containing a large electronegative group at the α-position. A modification of the RES-TOCSY NMR pulse sequence was used, allowing the enhancement of enantiomeric discrimination when the resolution of multiplets is insufficient. In future work, the development of different **2**-based CSAs will be continued.

## Supplementary Materials

Supplementary materials can be accessed at: http://www.mdpi.com/1420-3049/20/11/19732/s1.
