# Supplementary material for: Synthesis of Tertiary and Quaternary Amine Derivatives from Wood Resin as Chiral NMR Solvating Agents
Source: Molecules. 2015 Nov 23;20(11):20873–86. doi: 10.3390/molecules201119732 (PMC6332576; doi:10.3390/molecules201119732)
Supplement: Supplementary file 1 [file molecules-20-19732-s001.pdf]

Supplementary Materials: Synthesis of Tertiary and Quaternary Amine Derivatives from Wood Resin as Chiral NMR Solvating Agents

Tiina Laaksonen, Sami Heikkinen and Kristiina Wähälä

1. NMR Measurement and Processing Parameters

1.1. Compound Characterisation

All NMR experiments were performed using Varian UNITY INOVA 500 and Varian Mercury Plus 300 instruments at 27 °C. ^{1}H-NMR spectra were recorded with 4–16 transients, 8000–3565 Hz spectral width, and 1.9 s acquisition time at 500 MHz. ^{13}C-NMR spectra were recorded with 1828–816 transients, 31,446–20,000 Hz spectral width and 1–1.9 s acquisition time at 125 or 75 MHz. All 2D HSQC spectra were recorded using Varian UNITY INOVA 500 with 4 transients, 250–128 increments, 8000–4278 Hz spectral width in ^{1}H-dimension, 25133–241546 Hz spectral width in ^{13}C-dimension, 1.0–2.0 s relaxation delay, and 1.3 s acquisition time.

1.2. Resolution Experiments

All NMR measurements were recorded using Varian UNITY INOVA 500 at 27 °C. ^{1}H-NMR spectra were recorded with 16 transients, 8000 Hz spectral width, 5.0 s relaxation delay and 1.9 s acquisition time at 500 MHz. ^{19}F-NMR spectra were recorded with 16–32 transients, 19047 Hz spectral width, 5.0 s relaxation delay and 1.0 s acquisition time at 470 MHz. HSQC spectra were recorded using Varian UNITY INOVA 500 with 32 transients, 300 increments, 2.0 s relaxation delay and 0.128 s acquisition time. The spectral widths were 4157 Hz and 212653 Hz in ^{1}H- and ^{13}C-dimensions, respectively. NOESY experiments with 0.4 ms mixing time were recorded with 16 transients, 300 increments, 2.0 relaxation delay and 0.24 s acquisition time at 500 MHz. Spectral width in both dimensions was 4157 Hz.

2. Chiral Recognition Studies

Solutions of **3** and **4** (22.0 mM in $CDCl_3$) were prepared. Compound **2–9c** (1.0 eq or 2.0 eq) was dissolved in 0.5 mL (1.0 eq) of prepared solution and both ^{1}H- and ^{19}F-NMR spectra were recorded.

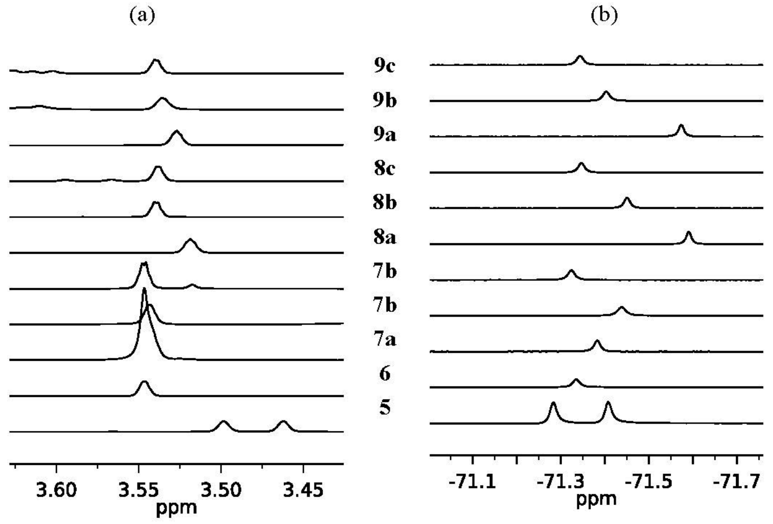

Figure S1. Host:guest ratio 1:1 both (**a**) ^{1}H- (–OC*H*$_3$) and (**b**) ^{19}F-NMR (–C*F*$_3$) spectra of **3**.

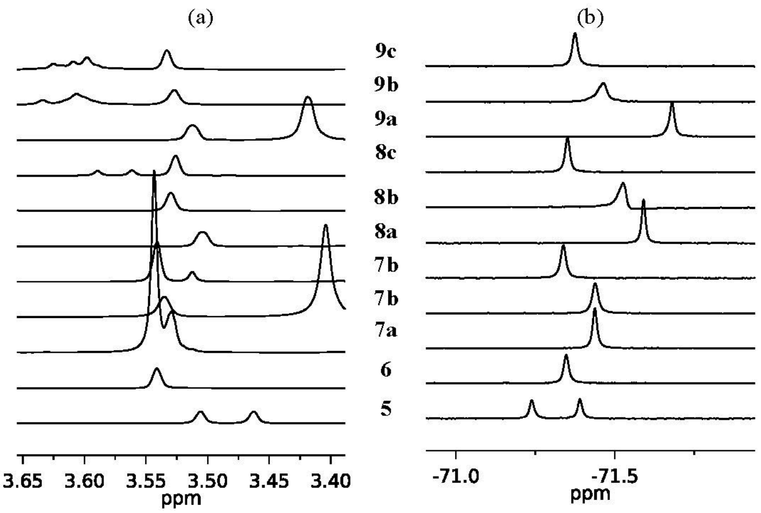

Figure S2. Host:guest ratio 2:1 both (**a**) ^{1}H- (–OCH_3) and (**b**) ^{19}F-NMR (–CF_3) spectra of **3**.

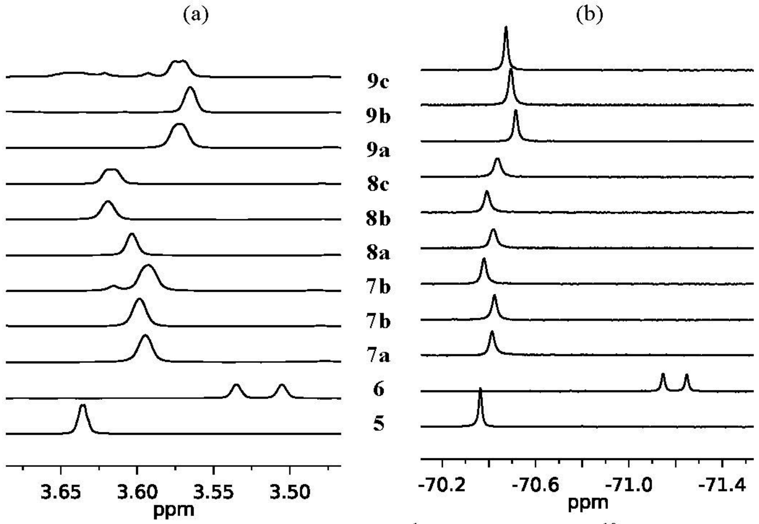

Figure S3. Host:guest ratio 1:1 both (**a**) ^{1}H- (–OCH_3) and (**b**) ^{19}F-NMR (–CF_3) spectra of **4**.

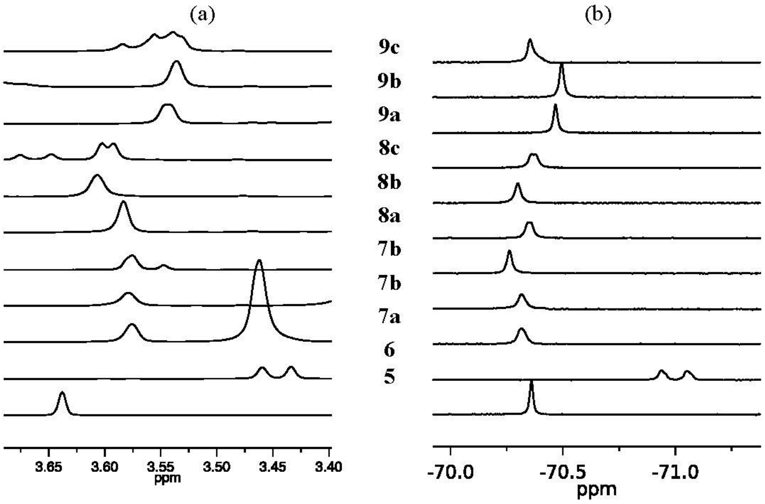

Figure S4. Host:guest ratio 2:1 both (**a**) ^{1}H- (–OCH_3) and (**b**) ^{19}F-NMR (–CF_3) spectra of **4**.

3. Titration

For titration, solutions containing the guest (2.0 mM) and host (46.6 mM) were prepared and 0.5 mL of guest solution was measured into an NMR tube. Guest was titrated by 2.5 or 5.0 μ doses of host solution. Both ^{1}H- and ^{19}F-NMR spectra were recorded.

3.1. Titration of **3** *with* **5**

Concentration of guest (**3**) is presumed to remain constant during titration.

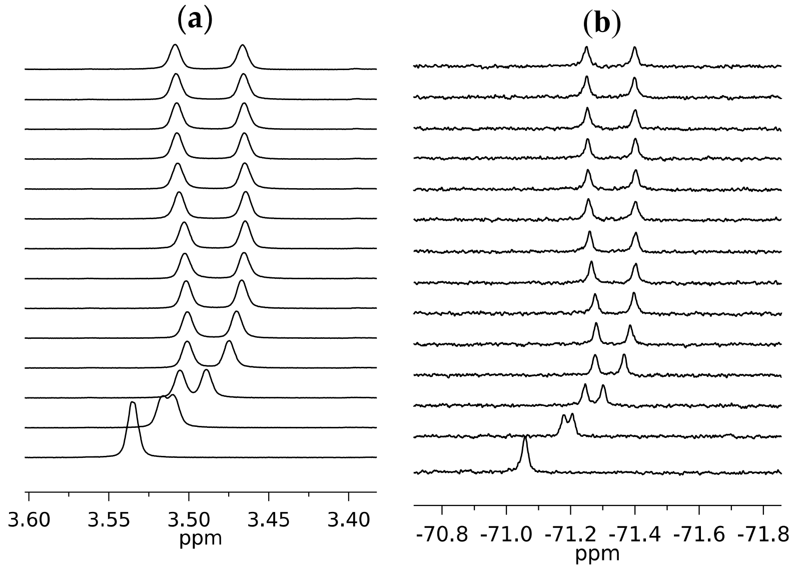

Figure S5. (**a**) Δδ on ^{1}H-NMR (**b**) Δδ on ^{19}F-NMR.

Table S1. Experimental data from titration of **3** with **5**.

c of 1 (μM)	V of 1 (mL)	δ of *S* (ppm)	δ of *R* (ppm)	Δδ of *S* (ppm)	Δδ of *R* (ppm)	δ of *S* (ppm)	δ of *R* (ppm)	Δδ of *S* (ppm)	Δδ of *R* (ppm)
0	0	3.535	3.535	0	0	−71.082	−71.082	0	0
0.461386139	0.005	3.516	3.51	0.019	0.025	−71.172	−71.201	0.09	0.119
0.91372549	0.01	3.505	3.489	0.03	0.046	−71.24	−71.296	0.158	0.214
1.357281553	0.015	3.501	3.475	0.034	0.06	−71.271	−71.362	0.189	0.28
1.575845411	0.0175	3.501	3.47	0.034	0.065	−71.274	−71.38	0.192	0.298
1.792307692	0.02	3.501	3.467	0.034	0.068	−71.271	−71.392	0.189	0.31
2.006698565	0.0225	3.502	3.465	0.033	0.07	−71.266	−71.396	0.184	0.314
2.219047619	0.025	3.503	3.465	0.032	0.07	−71.26	−71.399	0.178	0.317
2.637735849	0.03	3.505	3.464	0.03	0.071	−71.254	−71.399	0.172	0.317
3.048598131	0.035	3.503	3.464	0.032	0.071	−71.25	−71.397	0.168	0.315
3.451851852	0.04	3.507	3.465	0.028	0.07	−71.248	−71.397	0.166	0.315
3.847706422	0.045	3.507	3.465	0.028	0.07	−71.247	−71.396	0.165	0.314
4.236363636	0.05	3.507	3.465	0.028	0.07	−71.247	−71.396	0.165	0.314
4.618018018	0.055	3.508	3.466	0.027	0.069	−71.244	−71.394	0.162	0.312
4.992857143	0.06	3.508	3.466	0.027	0.069	−71.245	−71.394	0.163	0.312

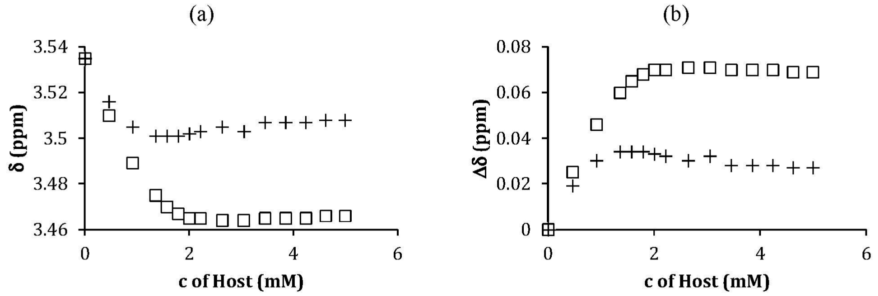

Figure S6. (**a**) The change of chemical shifts of *S* and *R* enantiomers of **3** and (**b**) the magnitude of non-equivalence (Δδ) of **3**, as a function of the concentration of **5** (□ *R* enantiomer and + *S* enantiomer) at ^{1}H-NMR.

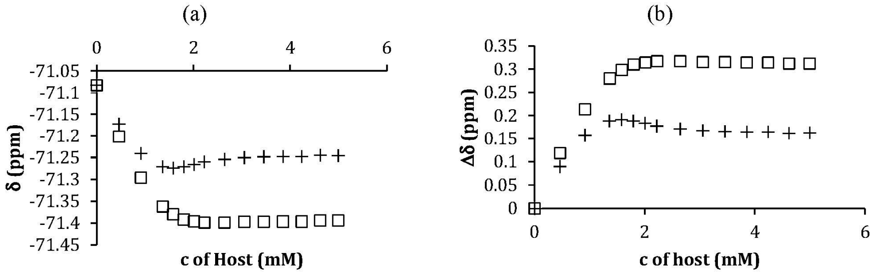

Figure S7. (**a**) The change of chemical shifts of *S* and *R* enantiomers of **3** and (**b**) the magnitude of non-equivalence (Δδ) of **3**, as a function of the concentration of **5** (□ *R* enantiomer and + *S* enantiomer) at ^{19}F-NMR.

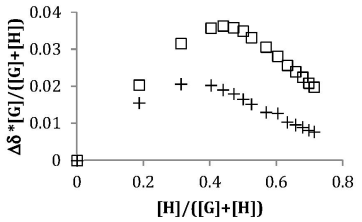

Figure S8. Job's plot.

3.2. *Titration of* **4** *with* **6**

Concentration of guest (**4**) is presumed to remain constant during titration.

Table S2. Experimental data from titration of [Bu_4N][MTPA] with **6**.

c of 1 (μM)	V of 1 (mL)	δ of *S* (ppm)	δ of *R* (ppm)	Δδ of *S* (ppm)	Δδ of *R* (ppm)	δ of *S* (ppm)	δ of *R* (ppm)	Δδ of *S* (ppm)	Δδ of *R* (ppm)
0	0	3.63	3.63	0	0	−70.611	−70.611	0	0
0.230693069	0.0025	3.612	3.612	0.018	0.018	−70.814	−70.814	0.203	0.203
0.461386139	0.005	3.601	3.601	0.029	0.029	−70.907	−70.926	0.296	0.315
0.91372549	0.01	3.565	3.547	0.065	0.083	−71.158	−71.22	0.547	0.609
1.357281553	0.015	3.535	3.51	0.095	0.12	−71.255	−71.344	0.644	0.733
1.792307692	0.02	3.499	3.464	0.131	0.166	−71.261	−71.383	0.65	0.772
2.219047619	0.025	3.495	3.462	0.135	0.168	−71.236	−71.354	0.625	0.743
2.637735849	0.03	3.491	3.459	0.139	0.171	−71.205	−71.322	0.594	0.711
3.048598131	0.035	3.49	3.458	0.14	0.172	−71.181	−71.297	0.57	0.686
3.451851852	0.04	3.487	3.456	0.143	0.174	−71.155	−71.271	0.544	0.66
3.847706422	0.045	3.483	3.454	0.147	0.176	−71.127	−71.238	0.516	0.627
4.236363636	0.05	3.48	3.452	0.15	0.178	−71.101	−71.21	0.49	0.599
4.618018018	0.055	3.477	3.451	0.153	0.179	−71.075	−71.182	0.464	0.571
4.992857143	0.06	3.475	3.45	0.155	0.18	−71.062	−71.17	0.451	0.559

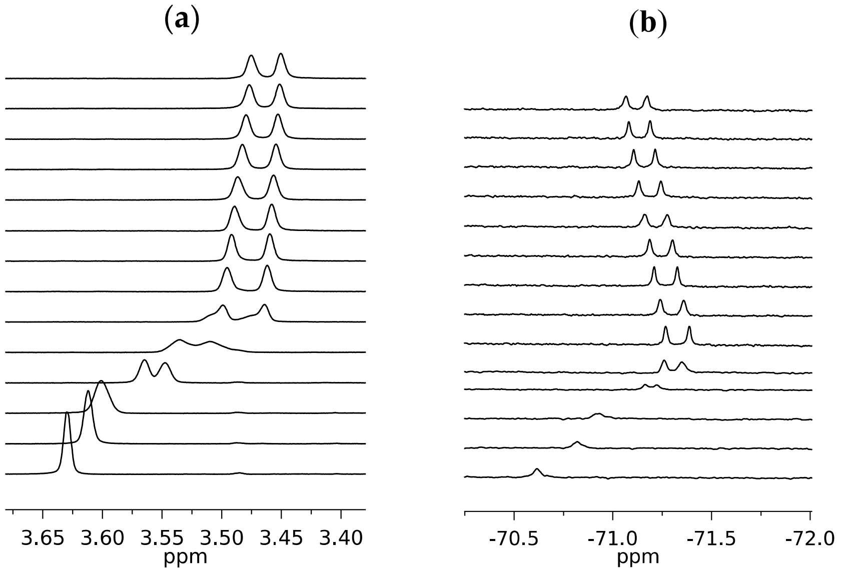

Figure S9. (**a**) Δδ on ^{1}H-NMR (**b**) Δδ on ^{19}F-NMR.

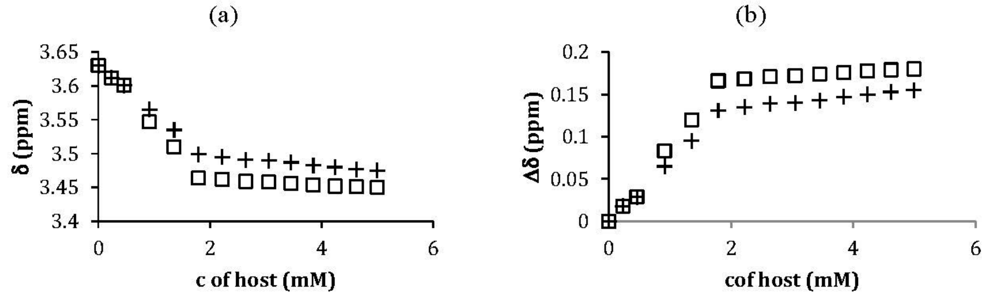

Figure S10. (**a**) The change of chemical shifts of *S* and *R* enantiomers of **4** and (**b**) the magnitude of non-equivalence (Δδ) of **4**, as a function of the concentration of **6** (□ *R* enantiomer and + *S* enantiomer) at ^{1}H-NMR.

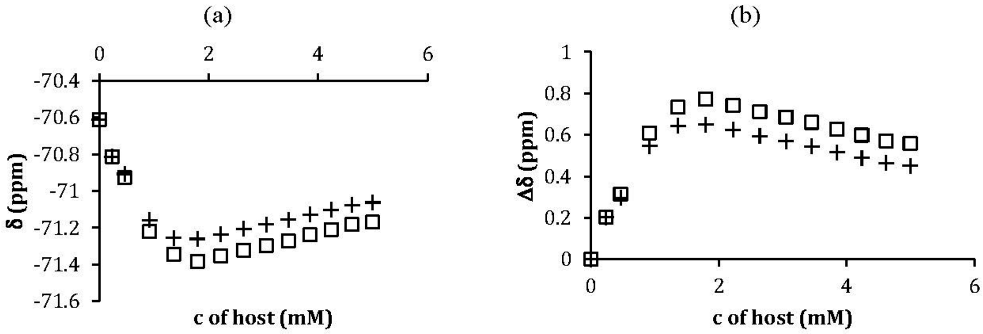

Figure S11. (**a**) The change of chemical shifts of *S* and *R* enantiomers of **4** and (**b**) the magnitude of non-equivalence (Δδ) of **4**, as a function of the concentration of **6** (□ *R* enantiomer and + *S* enantiomer) at ^{19}F-NMR.

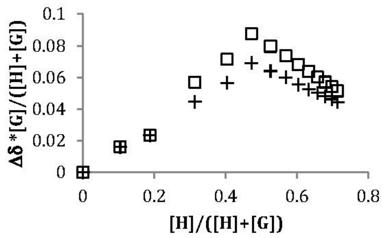

Figure S12. Job's plot.

4. Enantiomeric Excess Measurements

For ee determination studies, solutions of racemic **3** and *S*-**3** (as well as **4** and *S*-**4**) were made (2.0 mM). Mixtures of enantiomerically enriched samples were prepared in an NMR tube (0.5 mL, 1.0 eq), CSA added (22.5 µ, 46.6 mM, 1.0 eq) and spectra recorded.

4.1. Enantiomeric Excess Measurements of **3** *with* **5**

Table S3. ee% determined from ^{1}H-NMR spectra.

S (mL)	R/S (mL)	Expected S/R	Measured S/R	Area S	Area R	Expected ee% (*S*)	Measured ee% (*S*)
0	0.5	1	1.009820258	5758.292	5702.294	50	50.24430688
0.1	0.4	1.5	1.718569863	6547.05	3809.592	60	63.21595359
0.2	0.3	2.333	2.148910936	7841.881	3649.235	70	68.24298876
0.25	0.25	3	2.872851376	8265.406	2877.074	75	74.17923119
0.3	0.2	4	3.758461241	9028.0231	2402.053	80	78.98480308

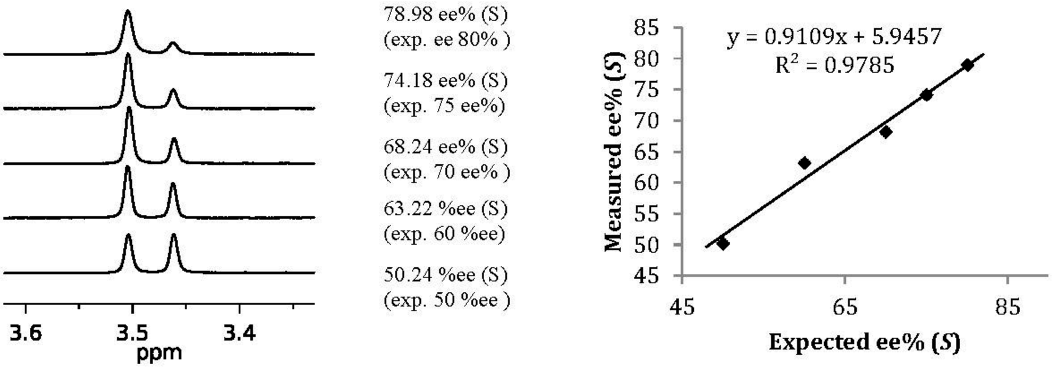

Figure S13. Results from ee% determination (^{1}H-NMR).

Table S4. ee% determined from ^{19}F-NMR spectra.

S (mL)	R/S (mL)	Expected S/R	Measured S/R	Area S	Area R	Expected ee% (*S*)	Measured ee% (*S*)
0	0.5	1	0.888463708	1115.388	1255.412	50	47.046904
0.1	0.4	1.5	1.299177815	1089.041	838.254	60	56.50619132
0.2	0.3	2.333	1.767438588	1105.22	625.323	70	63.86550349
0.25	0.25	3	3.516458349	1842.48	523.959	75	77.8587574
0.3	0.2	4	4.737135083	1444.992	305.035	80	82.5696975

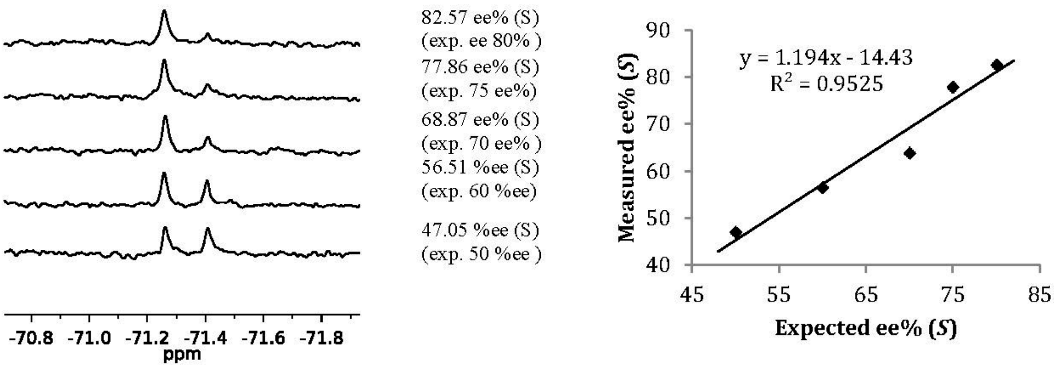

Figure S14. Results from ee% determination (^{19}F-NMR).

4.2. Enantiomeric Excess Measurements of **4** *with* **6**

Table S5. ee% determined from ^{1}H-NMR spectra.

S (mL)	R/S (mL)	Expected S/R	Measured S/R	Area S	Area R	Expected ee% (*S*)	Measured ee% (*S*)
0	0.5	1	1.160136853	6054.441	5218.73	50	53.70663676
0.1	0.4	1.5	1.466312442	7015.893	4784.719	60	59.45363681
0.2	0.3	2.333	2.430381361	9471.551	3897.146	70	70.84872221
0.25	0.25	3	3.289686745	8685.477	2640.214	75	76.68827447
0.3	0.2	4	4.025612356	8939.778	2220.725	80	80.10192731

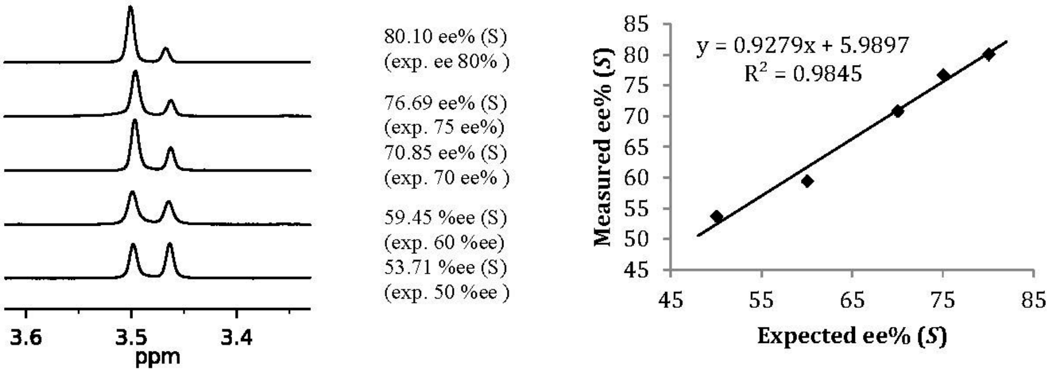

Figure S15. Results from ee% determination (^{1}H-NMR).

Table S6. ee% determined from ^{19}F-NMR spectra.

S (mL)	R/S (mL)	Expected S/R	Measured S/R	Area S	Area R	Expected ee% (*S*)	Measured ee% (*S*)
0	0.5	1	0.980533751	5213.697	5317.203	50	49.50856052
0.1	0.4	1.5	1.438918884	5676.158	3944.738	60	58.99822636
0.2	0.3	2.333	2.122697901	6909.076	3254.856	70	67.97640913
0.25	0.25	3	3.414083583	7432.518	2177.017	75	77.34524095
0.3	0.2	4	3.549660407	6804.699	1917.00	80	78.02033755

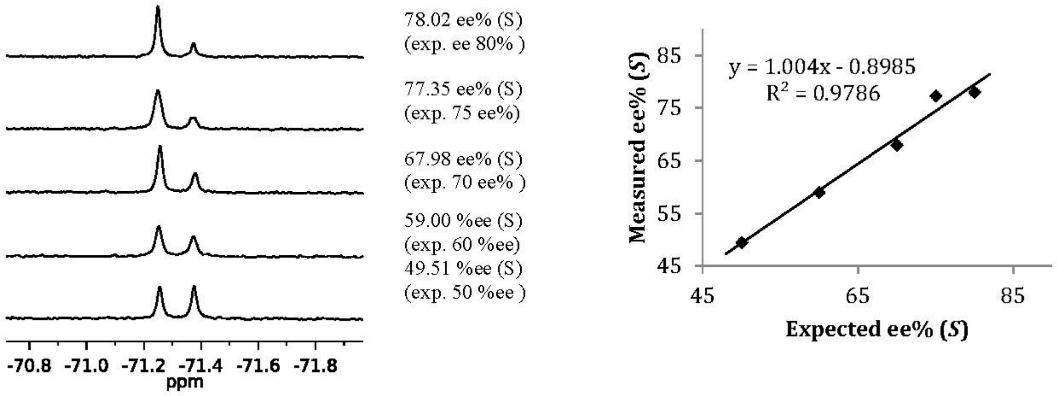

Figure S16. Results from ee% determination (^{19}F-NMR).

5. Resolution of racemic carboxylic acids

For chiral recognition ability determination study of racemic carboxylic acids, solutions of different racemic carboxylic acids (n-Bu_4N salts of carboxylic acids in the case of **6**) were prepared (2.0 mM). Experiment was performed by adding 22.5 μ of solution containing host (46.6 mM, 1.0 eq) to NMR tube containing 0.5 mL of guest (2.0 mM, 1.0 eq) and recording the spectra.

2.2. *Separation of Carboxylic Acids with* **5**

Table S7. Determination of magnitude of non-equivalences (Δδ) of five racemic carboxylic acids in the presence of **5**, using ¹H-NMR (500 MHz) in $CDCl_3$ at 27 °C.

No.	Racemic Carboxylic Acid	Actual Multiplet		Spectra (If Split Is Detected)	Δδ
**10a**	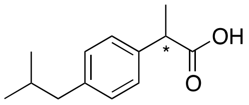	Me	d	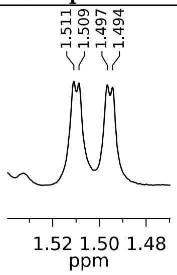	0.0023
		H	q	-	-
**11a**	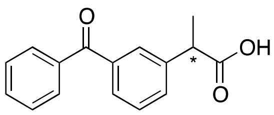	Me	d	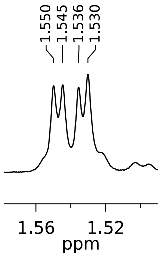	0.0059
		H	q	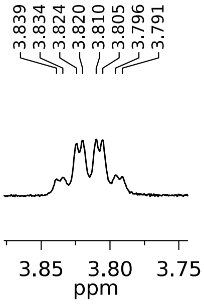	0.0047
**12a**	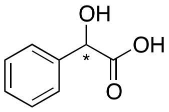	H	q	-	-
**13a**	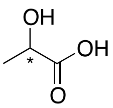	H	q	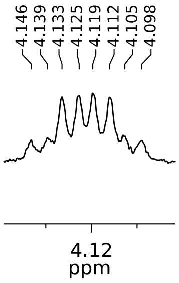	0.0081
		Me	d	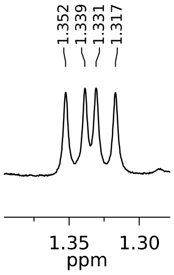	0.013

Table S7. *Cont.*

No.	Racemic Carboxylic Acid		Actual Multiplet	Spectra (If Split Is Detected)	Δδ
**14a**	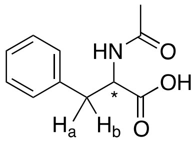	H	q	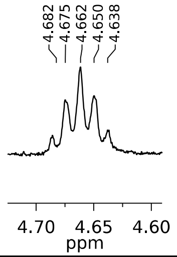	0.012
		NH	d	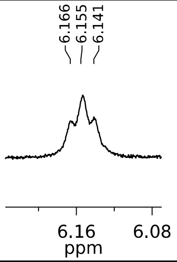	0.013
		Me	s	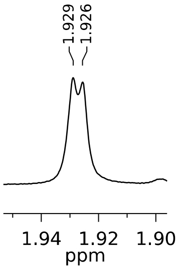	0.0028
		H_a<br>H_b	Doublet of AB quartet	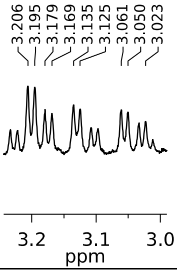	0.072<br>0.027
**15a**	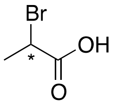	H	q	-	-
		Me	d	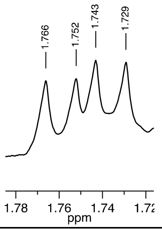	0.023
**16a**	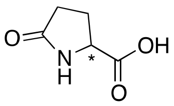	H	t	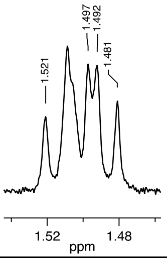	0.016
		NH	br.s	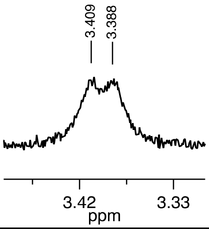	0.020

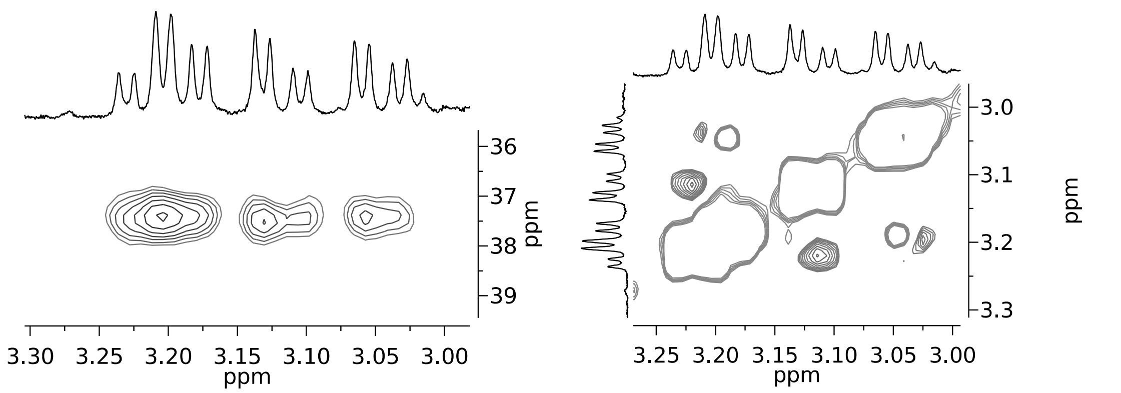

(**a**) (**b**)

Figure S17. (**a**) HSQC and (**b**) NOESY spectra of **14a** in the presence of **5**.

5.2. Separation of Carboxylic Acid n-Bu₄N Salts with **6**

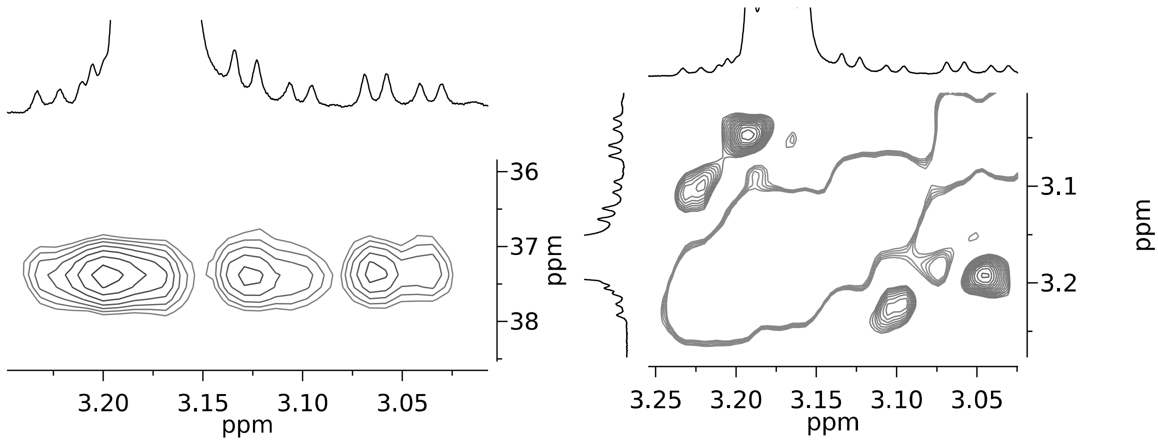

(**a**) (**b**)

Figure S18. (**a**) HSQC and (**b**) NOESY of **14b** in the presence of **6.**

Table S8. Determination of magnitude of non-equivalences (Δδ) of five racemic carboxylic acid *n*-Bu_4N salts in the presence of **6**, using ^{1}H-NMR (500 MHz) in $CDCl_3$ at 27 °C.

No.	***n*-Bu₄N Salt of Racemic Carboxylic Acid**	**Actual Multiplet**		**Spectra**	**Δδ**
**10b**	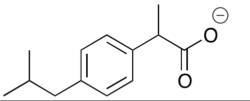	Me	d	-	-
		H	q	-	-
**11b**	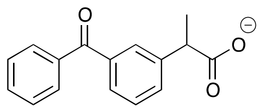	Me	d	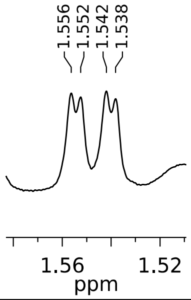	0.0041

Table S8. *Cont.*

No.	*n*-Bu4N Salt of Racemic Carboxylic Acid	Actual Multiplet		Spectra	Δδ
**11b**	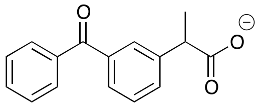	H	q	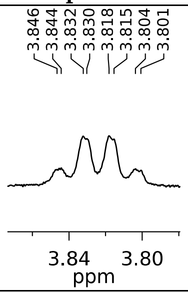	0.0021
**12b**	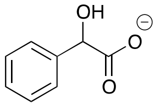	H	q	-	-
**13b**	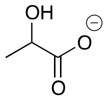	H	q	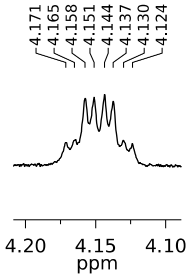	0.0068
		Me	d	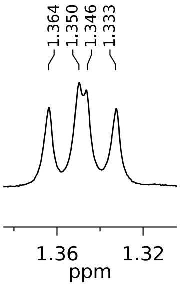	0.017
**14b**	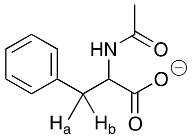	H	q	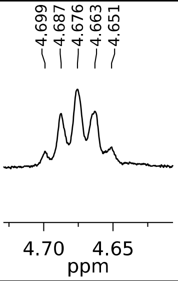	0.012
		NH	d	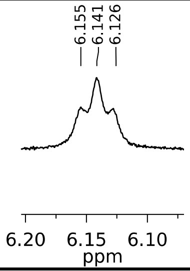	0.014

Table S8. *Cont.*

<table>
<tr><th>No.</th><th>n-Bu₄N Salt of Racemic Carboxylic Acid</th><th colspan="2">Actual Multiplet</th><th>Spectra</th><th>Δδ</th></tr>
<tr><td rowspan="2">14b</td><td rowspan="2">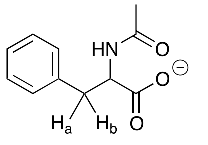
</td><td>Me</td><td>s</td><td>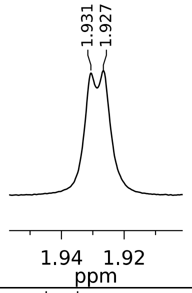
</td><td>0.0039</td></tr>
<tr><td>Ha
Hb</td><td>Doublet of AB
quartet</td><td>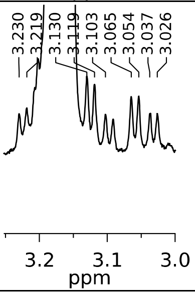
</td><td>0.065
0.036</td></tr>
<tr><td rowspan="2">15a</td><td rowspan="2">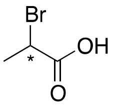
</td><td>H</td><td>q</td><td>-</td><td>-</td></tr>
<tr><td>Me</td><td>d</td><td>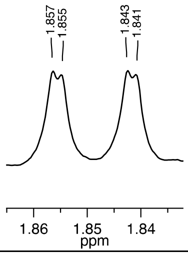
</td><td>0.002</td></tr>
<tr><td rowspan="2">16a</td><td rowspan="2">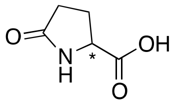
</td><td>H</td><td>t</td><td>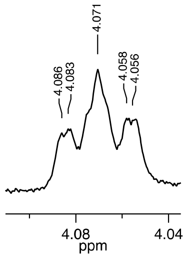
</td><td>0.003</td></tr>
<tr><td>NH</td><td>br.s</td><td>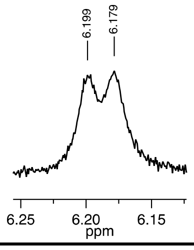
</td><td>0.020</td></tr>
</table>

6. Modified RES-TOCSY, A Phase Sensitive Version

Recently, Lokesh *et al.*, reported new homonuclear 2D technique, RES-TOCSY [1], for obtaining improved separation of enantiomer spectra when chiral resolving agents are utilized. RES-TOCSY is based on homonuclear decoupling in f_1-dimension for selectively excited protons and for the whole coupled homonuclear spin system. Homonuclear decoupling is achieved by applying selective refocusing pulse followed by non-selective 180° pulse in between t_1-period. The net effect is thus 0° pulse for selected protons and 180° pulse for other protons leading to homonuclear decoupling for the selected protons (and thus collapse of the multiplets into a single lines in f_1-dimension) while retaining chemical shift evolution of the selected protons. Subsequent TOCSY-period transfers the magnetization through entire coupled homonuclear spin system. The original RES-TOCSY is a

magnitude mode experiment with two selective pulses (90° and 180°) and the coherence selection is performed by phase cycling. The modified RES-TOCSY pulse sequence utilized in this work is presented in Figure S19 and pulse sequence code in Varian syntax is shown in the end of this section. The presented pulse sequence is actually a simplified BASHD-TOCSY [2,3] where a single selective 180° pulse flanked by pulsed field gradient of opposite amplitudes is applied, followed by a non-selective 180° pulse in the middle of t_1-period. Gradients with opposite polarities (g_1) label the desired magnetization and, as with original RES-TOCSY, the net effect of the two pulses allows selective homonuclear decoupling in f_1-dimesion. After t_1-period TOCSY transfers the magnetization throughout the spin system and finally magnetization with gradient encoding is refocused during spin-echo period prior to the acquisition (AQ). Phase sensitive data is obtained with echo-anti echo method, where N- and P-type coherences are recorded separately by inverting the polarity of the refocusing gradient g_2. Since all signals in the f_1-dimension of the resulting spectrum are originating from the excitation bandwidth of the selective pulse, it is possible to utilize small spectral width in the indirectly detected dimension.

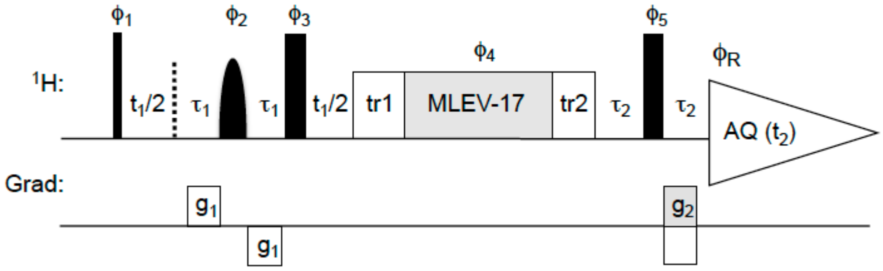

Figure S19. Pulse sequence for phase sensitive BASHD-TOCSY-based [2,3] RES-TOCSY [1] utilizing single selective 180° pulse and gradient selection. Narrow and wide black bars represent hard 90° and 180° rectangular pulses, respectively, while black half ellipse indicates multiplet selective 180° pulse. Low-power TOCSY spinlock sequence is indicated by grey rectangle denoted MLEV-17 [4] flanked by two trim pulses (tr1 and tr2). Pulsed field gradients are represented by rectangles marked with g_1 and g_2. Delay t_1 represents incremented delay while delays τ_1 and τ_2 include gradient pulse duration and following eddy current recovery delay. Phase cycle: ϕ_1 = y, −y; ϕ_2 = x, x, y, y; ϕ_3 = x, x, x, x, y, y, y, y; ϕ_4 = x, −x; ϕ_5 = x; ϕ_R = x, −x, −x, x, −x, x, x, −x. The N-and P-type coherences are separately recorded by inverting the sign of the gradient g_2. Axial peak displacement is obtained using States-TPPI method [5] by inverting the phases ϕ_1 and ϕ_R on every second increment.

Figure S18 show expansion of 2D spectrum recorded with pulse sequence presented in Figure S19 from 2.0 mM racemic *N*-acetyl-phenylalanine sample doped with CSA **5** with 1:1 host:guest ratio in $CDCl_3$. The selective 180° pulse was applied at enantiomeric resonances at 4.67 ppm. The separation between the enantiomers $\Delta\delta$ = 6.1 Hz for the selectively inverted proton is present in each expansion (TOCSY transfer). Due to homonuclear decoupling, multiplets collapse into single lines in f_1-dimension and thus allow good separation of the enantiomeric signals. Obviously, if the CSA induced $\Delta\delta$ between the enantiomer signals is of same magnitude as natural line width, baseline separation is naturally not possible even with homonuclear decoupling. Figure S21 shows expansion of the modified RES-TOCSY spectrum recorded from 2.0 mM ketoprofen sample doped with the same CSA as well as f_2-traces extracted from locations marked by arrows. Since $\Delta\delta$ and linewidth in f_1-dimension are of similar magnitude (~2.0 Hz), some cross talk between the traces is unavoidable. The data in Figure S19 was acquired by selectively inverting multiplet region at 1.55 ppm. Other acquisition and processing parameters were identical to data presented in Figure S20.

The results by Lokesh *et al.* [1], suggested the possibility to utilize 2D RES-TOCSY to actually estimate ee% values with good accuracy. Obviously, in order to obtain reliable results, there should not be significant differences in host-guest association constants between the enantiomers. Such big differences could easily result in a significant difference also in transverse relaxation times, T_2 (*i.e.*, one enantiomer has significantly shorter T_2 than the other due to stronger interaction with the host), and thus the corresponding magnetization experiences more attenuation during the pulse sequence

(relatively long selective pulses as well as TOCSY mixing emphasize this). This would lead to an underestimation of the corresponding enantiomer signal and thus erroneous ee%. The aforementioned result could be the most pronounced when the chiral auxiliary is a very large molecule or polymer. Then, depending on the differences in association constant, the molecular mobilities can be very different, leading to differences in T_2. For small auxiliaries and relatively weak association, this is likely not a major problem. One could speculate that, if a significant difference appears in line the widths of relevant signals, RES-TOCSY-based method cannot be reliably utilized for ee% determination. In general, one may assume only small differences in quantitation affecting NMR properties (relaxation times, coupling constants) of enantiomer signals when chiral auxiliaries are used. Provided coupling constants (heteronuclear, homonuclear), relaxation times (T_1, T_2) or internuclear distances remain essentially the same in both resolved enantiomers, rather many classic 2D NMR methods can be used for ee% determination, at least at a semi quantitative level.

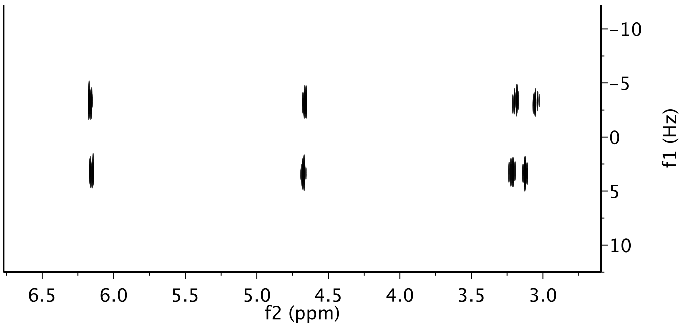

Figure S20. Expansion of modified RES-TOCSY spectrum of 2.0 mM racemic N-acetylated phenylalanine sample with equimolar amount of CSA **5** in $CDCl_3$. The 2D experiment was performed at 27 °C using a Varian Unity INOVA 500 spectrometer equipped with PFG inverse-detection probehead incorporating z-gradient coil capable of delivering gradient amplitudes up to up to 20 G/cm. Spectral widths were 4550 Hz (f_2) and 25 Hz in the ^{1}H-decoupled dimension (f_1). The 2D data was acquired using 16 steady state scans, 8 transients and 25 increments. The number of acquired complex point was 4550 corresponding acquisition time of 1.0 s. The relaxation delay was 0.5 s. The duration for hard rectangular ^{1}H 90° pulse was 7.1 μs. The pulses in MLEV-17 TOCSY-period were applied at RF-power of 7.7 kHz. Durations for trim pulses were 1.0 ms (tr1) and 1.6 ms (tr2). TOCSY mixing time was 60 ms. RE-BURP shape[6] was used for multiplet selective refocusing pulse of duration 97.5 ms, corresponding excitation bandwidth of 50 Hz. Selective pulse was applied at protons resonating at 4.67 ppm. Gradient strengths (durations) were: g1 = 5.0 G/cm (1.0 ms), and g2 −5.0 G/cm (2.0 ms). All the gradients were block shaped. The data was apodized using gaussian functions in both dimensions (1.5 Hz gaussian broadening) and zero-filled up to 8192 × 256 complex points prior to Fourier transformation (MestReNova 10.0.1, Mestrelab Research S.L.)

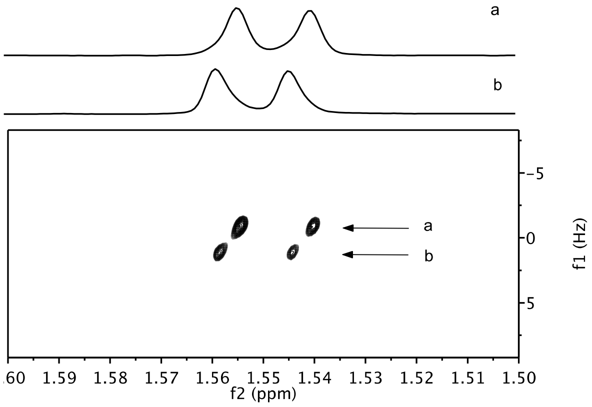

Figure S21. Expansion of the selectively inverted region of the modified RES-TOCSY spectrum from a 2.0 mM racemic ketoprofen sample with an equimolar amount of CSA **5** in $CDCl_3$. The traces (**a**) and (**b**) represent doublets of each enantiomeric signal.

References

1. Lokesh,N.; Chaudhari, S.R.; Nagarajarao, S. RES-TOCSY: A simple approach to resolve overlapped ^{1}H-NMR spectra of enantiomers. *Org. Biomol. Chem.* **2014**, *12*, 993–997.
2. Krishnamurthy, V.V. Application of Semi-Selective Excitation Sculpting for Homonuclear Decoupling During Evolution in Multi-Dimensional NMR. *Magn. Reson. Chem.* **1997**, *35*, 9–12.
3. Roumestand, C.; Mutzenhardt, P.; Delay, C.; Canet, D. Gradient-Enhanced Band-Filtering Experiments. *Magn. Reson. Chem.* **1996**, *34*, 807–814.
4. Bax, A.; Davis, D.G. Mlev-17-Based Two-Dimensional Homonuclear Magnetization Transfer Spectroscopy. *J. Magn. Reson.* **1985**, *65*, 355–360.
5. Marion, D.; Ikura, M.; Tschudin, R.; Bax, A. Rapid recording of 2D NMR spectra without phase cycling. Application to the study of hydrogen exchange in proteins. *J. Magn. Reson.* **1989**, *85*, 393–399.
6. Helen Geen, Ray Freeman. Band-selective radiofrequency pulses. *J. Magn. Reson.* **1969**, *93*, 93–141.

6.1. Pulse Sequence

```
/* Modified RES-TOCSY –with MLEV17 spinlock and gradient coherence
 selection
 Selective 180 pulse applied at selfrq-frequency. During t1, transmitter is at
 selfrq, while during detection transmitter is at tof.
 Use normal sw and small sw1

 Features included:
 Axial peak displacement in F1
 Randomization of Magnetization prior to relaxation delay G-90-G - with PFG
 Solvent suppression during relaxation delay

 For phase sensitive data use f1coef = '1 0 1 0 0 1 0 -1'

 Paramters
 sspul    : y - selects magnetization randomization option
 hsglvl  : Homospoil gradient level (DAC units)
 hsgt   : Homospoil gradient time
 satmode : y - selects presaturation during relaxation delay
 satfrq: presaturation frequency
 satdly  : presaturation delay
 satpwr : presaturation power
 selfrq   : Selective frequency (for selective 90 and 180)
 selpwr180     : Power for selective 180 deg pulse
 selpw180  : Selective 180 deg pulse width
 selshape180 : Selective 180 deg pulse shape
 gzlvl1  : Coherence selection gradient level
 gt1     : Coherence selection gradient duration
 gzlvl2  : Coherence selection gradient level
 gt2     : Coherence selection gradient duration
 gstab    : Eddy current recovery delay
```

```
 mix   : TOCSY (MLEV17) spinlock mixing time
 trim1   : trim pulse preceeding spinlock
 trim2   : trim pulse after spinlock
 slpwr   : spin-lock power level
 slpw    : 90 deg pulse width for spinlock
 d1   : Relaxation delay
 d2   : Evolution delay

S.H. 17.4.2015

*/


#include <standard.h>

static double d2_init = 0.0;

static int ph11[2] = {1,3},
 ph12[4] = {0,0,1,1},
 ph13[8] = {0,0,0,0,1,1,1,1},
 ph2[2] = {0,2},
 ph3[2] = {3,1},
 ph4[2] = {0,2},
 ph5[2] = {1,3},
 ph6[2] = {2,0},
 ph7[2] = {0,2},
 ph8[2] = {0,2},
 ph31[8] = {0,2,2,0,2,0,0,2};

pulsesequence()
{
 char sspul[MAXSTR],
 satmode[MAXSTR],
 selshape180[MAXSTR];

 int t1_counter,
 icosel;

 double selfrq = getval("selfrq"),
 selpwr180 = getval("selpwr180"),
 selpw180 = getval("selpw180"),
 slpwr = getval("slpwr"),
```

```
slpw = getval("slpw"),
trim1 = getval("trim1"),
trim2 = getval("trim2"),
mix = getval("mix"),
hsglvl = getval("hsglvl"),
hsgt = getval("hsgt"),
gzlvl1 = getval("gzlvl1"),
gt1 = getval("gt1"),
gzlvl2 = getval("gzlvl2"),
gt2 = getval("gt2"),
gstab = getval("gstab"),
satfrq = getval("satfrq"),
satpwr = getval("satpwr"),
satdly = getval("satdly"),
phase = getval("phase"),
cycles;

/* LOAD VARIABLES */

getstr("satmode",satmode);
getstr("sspul", sspul);

getstr("selshape180",selshape180);

cycles = (mix - (trim1+trim2) ) / ( (64.66*slpw)+ 98.0e-6 );
cycles = 2.0*(double) (int) (cycles/2.0);
initval(cycles, v9); /* V9 is the MIX loop count */

settable(t11,2,ph11);
settable(t12,4,ph12);
settable(t13,8,ph13);
settable(t2,2,ph2);
settable(t3,2,ph3);
settable(t4,2,ph4);
settable(t5,2,ph5);
settable(t6,2,ph6);
settable(t7,2,ph7);
settable(t8,2,ph8);
settable(t31,8,ph31);

if (phase == 1)
icosel = +1;
```

```
if (phase == 2)
icosel = -1;

/* Calculate modifications to phases based on current t1/t2 values
 for axial displacement */

if(ix==1)
d2_init = d2;

t1_counter = (int) ( (d2-d2_init)*sw1 + 0.5);
if(t1_counter % 2)
{
tsadd(t11,2,4);
tsadd(t31,2,4);
}

if(ix == 1)
{
printf("The precise mixing time is %f\n",(cycles*64.66*slpw + 98.0e-6 + trim1 + trim2));
}

/* CHECK VALIDITY OF PARAMETERS */

if( satpwr > 8 )
{
printf("Presaturation power satpwr too large !!! ");
abort(1);
}

if(mix > 0.250)
{
printf("The time mix is too long");
abort(1);
}

if(slpwr > 44)
{
printf("Tocsy spinlock power slpwr is too high; must be < 45\n");
abort(1);
}
```

```
/* BEGIN ACTUAL PULSE SEQUENCE CODE */
 status(A);

 obspower(tpwr);

 obsoffset(tof);

 if (sspul[0] == 'y')
 {
 zgradpulse(hsglvl,hsgt);
 rgpulse(pw,zero,rof1,rof1);
 zgradpulse(hsglvl,hsgt);
 }

 delay(d1);

 if (satmode[0] == 'y')
 {
 if (satfrq != tof)
 obsoffset(satfrq);
 obspower(satpwr);
 rgpulse(satdly,zero,rof1,rof1);
 obspower(tpwr);
 if (satfrq != tof)
 obsoffset(tof);
 }

 obsoffset(selfrq);

 status(B);

 rgpulse(pw,t11,2.0e-6,0.0);

 delay(0.5*d2);

 txphase(t12);

 obspower(selpwr180);

 zgradpulse(1.0*gzlvl1,gt1);
 delay(gstab);
```

```
shaped_pulse(selshape180,selpw180,t12,20.0e-6,20.0e-6);

txphase(t13);
obspower(tpwr);

zgradpulse(-1.0*gzlvl1,gt1);
delay(gstab);

rgpulse(2.0*pw,t13,2.0e-6,2.0e-6);

delay(0.5*d2);

obspower(slpwr);

txphase(t2);

obsoffset(tof);

if (cycles > 1.0)
{
rcvroff();
txphase(t2);
rgpulse(trim1, t2, 1.0e-6, 1.0e-6);

starthardloop(v9);
/* A */
txphase(t3);
rgpulse(slpw, t3, 1.0e-6, 1.0e-6);
txphase(t4);
rgpulse(2.0*slpw, t4, 1.0e-6, 1.0e-6);
txphase(t3);
rgpulse(slpw, t3, 1.0e-6, 1.0e-6);
/* A */
txphase(t3);
rgpulse(slpw, t3, 1.0e-6, 1.0e-6);
txphase(t4);
rgpulse(2.0*slpw, t4, 1.0e-6, 1.0e-6);
txphase(t3);
rgpulse(slpw, t3, 1.0e-6, 1.0e-6);
/* B */
txphase(t5);
rgpulse(slpw, t5, 1.0e-6, 1.0e-6);
```

```
txphase(t6);
rgpulse(2.0*slpw, t6, 1.0e-6, 1.0e-6);
txphase(t5);
rgpulse(slpw, t5, 1.0e-6, 1.0e-6);
/* B */
txphase(t5);
rgpulse(slpw, t5, 1.0e-6, 1.0e-6);
txphase(t6);
rgpulse(2.0*slpw, t6, 1.0e-6, 1.0e-6);
txphase(t5);
rgpulse(slpw, t5, 1.0e-6, 1.0e-6);

/* B */
txphase(t5);
rgpulse(slpw, t5, 1.0e-6, 1.0e-6);
txphase(t6);
rgpulse(2.0*slpw, t6, 1.0e-6, 1.0e-6);
txphase(t5);
rgpulse(slpw, t5, 1.0e-6, 1.0e-6);
/* A */
txphase(t3);
rgpulse(slpw, t3, 1.0e-6, 1.0e-6);
txphase(t4);
rgpulse(2.0*slpw, t4, 1.0e-6, 1.0e-6);
txphase(t3);
rgpulse(slpw, t3, 1.0e-6, 1.0e-6);
/* A */
txphase(t3);
rgpulse(slpw, t3, 1.0e-6, 1.0e-6);
txphase(t4);
rgpulse(2.0*slpw, t4, 1.0e-6, 1.0e-6);
txphase(t3);
rgpulse(slpw, t3, 1.0e-6, 1.0e-6);
/* B */
txphase(t5);
rgpulse(slpw, t5, 1.0e-6, 1.0e-6);
txphase(t6);
rgpulse(2.0*slpw, t6, 1.0e-6, 1.0e-6);
txphase(t5);
rgpulse(slpw, t5, 1.0e-6, 1.0e-6);

/* B */
```

```
txphase(t5);
rgpulse(slpw, t5, 1.0e-6, 1.0e-6);
txphase(t6);
rgpulse(2.0*slpw, t6, 1.0e-6, 1.0e-6);
txphase(t5);
rgpulse(slpw, t5, 1.0e-6, 1.0e-6);
/* B */
txphase(t5);
rgpulse(slpw, t5, 1.0e-6, 1.0e-6);
txphase(t6);
rgpulse(2.0*slpw, t6, 1.0e-6, 1.0e-6);
txphase(t5);
rgpulse(slpw, t5, 1.0e-6, 1.0e-6);
/* A */
txphase(t3);
rgpulse(slpw, t3, 1.0e-6, 1.0e-6);
txphase(t4);
rgpulse(2.0*slpw, t4, 1.0e-6, 1.0e-6);
txphase(t3);
rgpulse(slpw, t3, 1.0e-6, 1.0e-6);
/* A */
txphase(t3);
rgpulse(slpw, t3, 1.0e-6, 1.0e-6);
txphase(t4);
rgpulse(2.0*slpw, t4, 1.0e-6, 1.0e-6);
txphase(t3);
rgpulse(slpw, t3, 1.0e-6, 1.0e-6);

/* A */
txphase(t3);
rgpulse(slpw, t3, 1.0e-6, 1.0e-6);
txphase(t4);
rgpulse(2.0*slpw, t4, 1.0e-6, 1.0e-6);
txphase(t3);
rgpulse(slpw, t3, 1.0e-6, 1.0e-6);
/* B */
txphase(t5);
rgpulse(slpw, t5, 1.0e-6, 1.0e-6);
txphase(t6);
rgpulse(2.0*slpw, t6, 1.0e-6, 1.0e-6);
txphase(t5);
rgpulse(slpw, t5, 1.0e-6, 1.0e-6);
```

```
 /* B */
 txphase(t5);
 rgpulse(slpw, t5, 1.0e-6, 1.0e-6);
 txphase(t6);
 rgpulse(2.0*slpw, t6, 1.0e-6, 1.0e-6);
 txphase(t5);
 rgpulse(slpw, t5, 1.0e-6, 1.0e-6);
 /* A */
 txphase(t3);
 rgpulse(slpw, t3, 1.0e-6, 1.0e-6);
 txphase(t4);
 rgpulse(2.0*slpw, t4, 1.0e-6, 1.0e-6);
 txphase(t3);
 rgpulse(slpw, t3, 1.0e-6, 1.0e-6);

 txphase(t7);
 rgpulse(0.66*slpw, t7, 1.0e-6, 1.0e-6);
 endhardloop();

 txphase(t8);
 rgpulse(trim2, t8, 1.0e-6, 1.0e-6);

 rcvron();
 }

 delay(gt2 + gstab + 2.0*GRADIENT_DELAY);

 obspower(tpwr);

 rgpulse(2.0*pw,zero,2.0e-6,2.0e-6);

 zgradpulse(icosel*-1.0*gzlvl2,gt2);
 delay(gstab + POWER_DELAY);

 status(C);

 setreceiver(t31);
}
```

7. NMR Spectra of Synthesised CSAs

7.1. ¹H, ¹³C and HSQC Spectra of Compound **5**

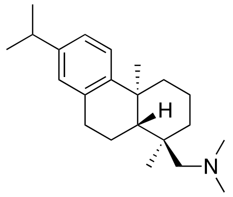

Parameter	Value
Origin	Varian
Spectrometer	inova
Solvent	CDCL3
Temperature	27.0
Pulse Sequence	s2pul
Experiment	1D
Probe	unibody
Number of Scans	8
Receiver Gain	16
Relaxation Delay	5.0000
Pulse Width	0.0000
Acquisition Time	1.8970
Spectrometer Frequency	499.82
Spectral Width	6712.5
Lowest Frequency	-711.3
Nucleus	1H
Acquired Size	12734
Spectral Size	65536

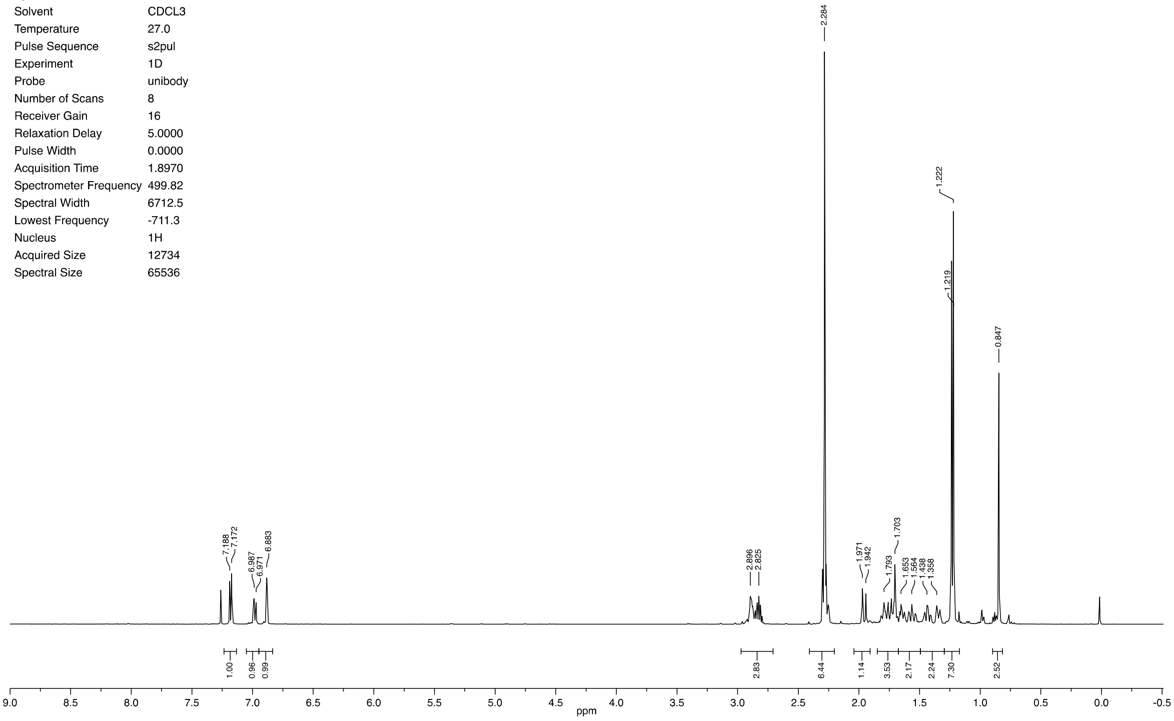

Figure S22. ¹H Spectra of Compound **5**.

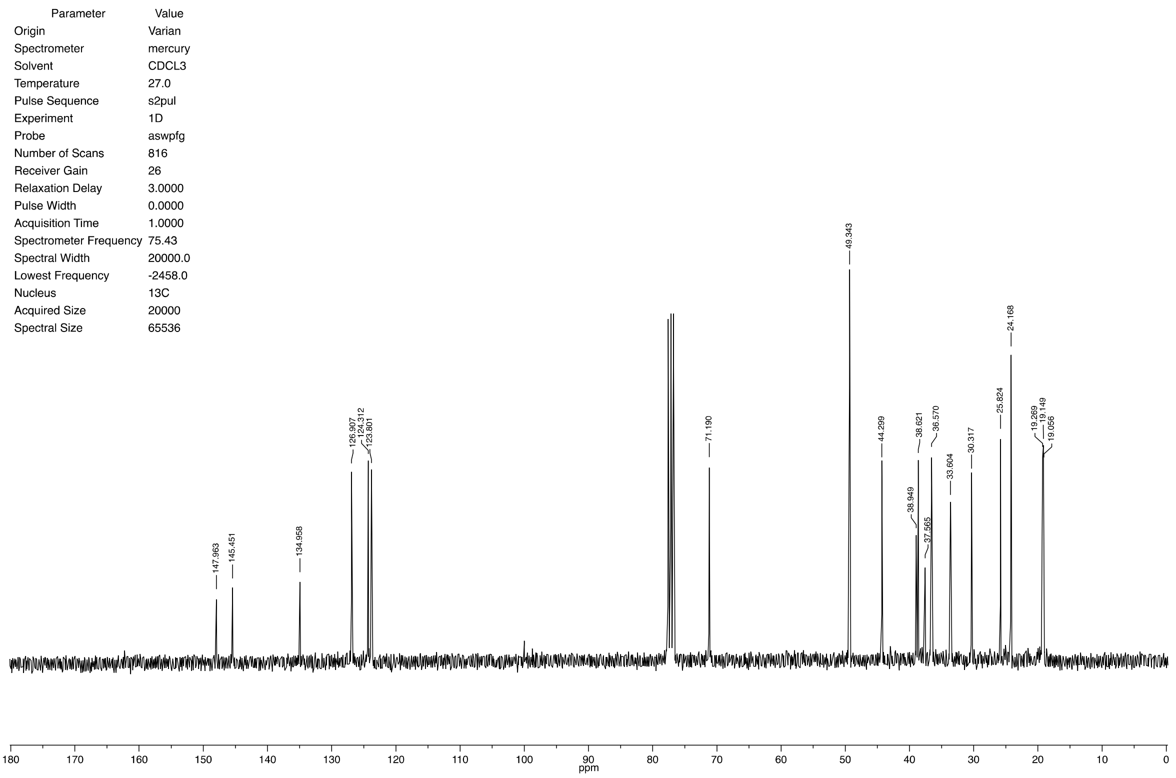

Figure S23. ^{13}C Spectra of Compound **5**.

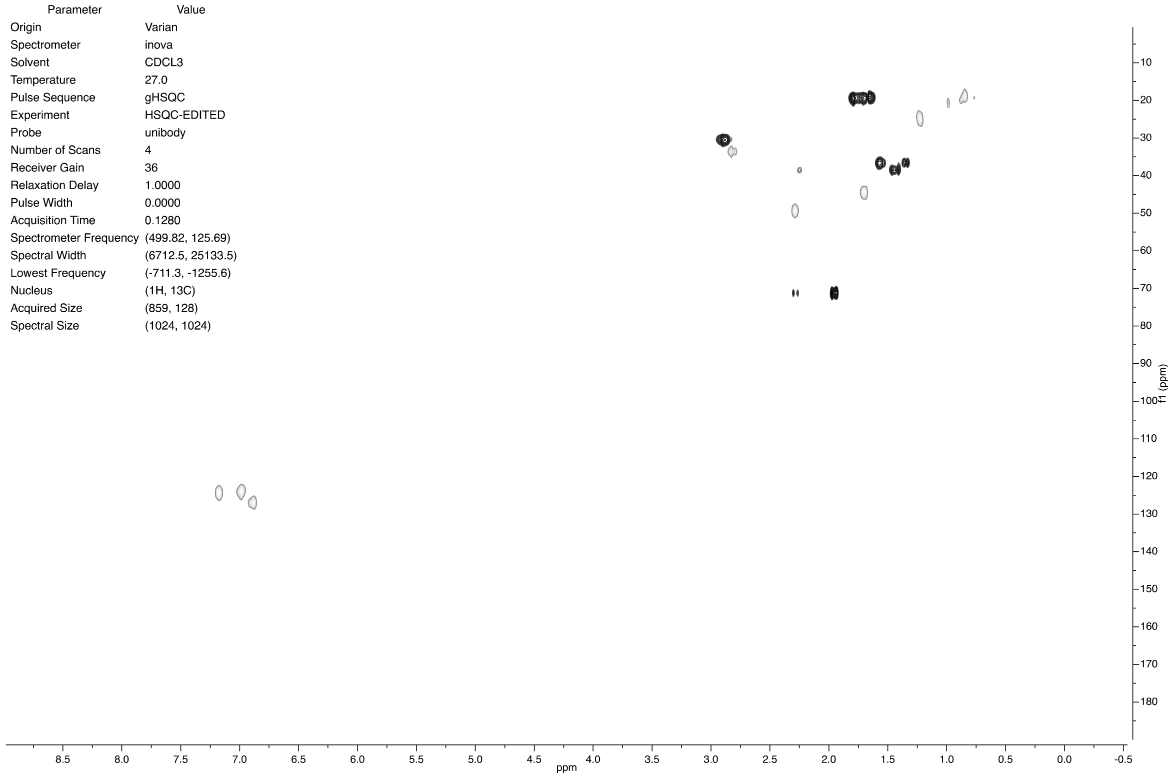

Figure S24. HSQC Spectra of Compound **5**.

7.2. ^{1}H, ^{13}C and HSQC Spectra of Compound **6**

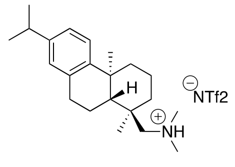

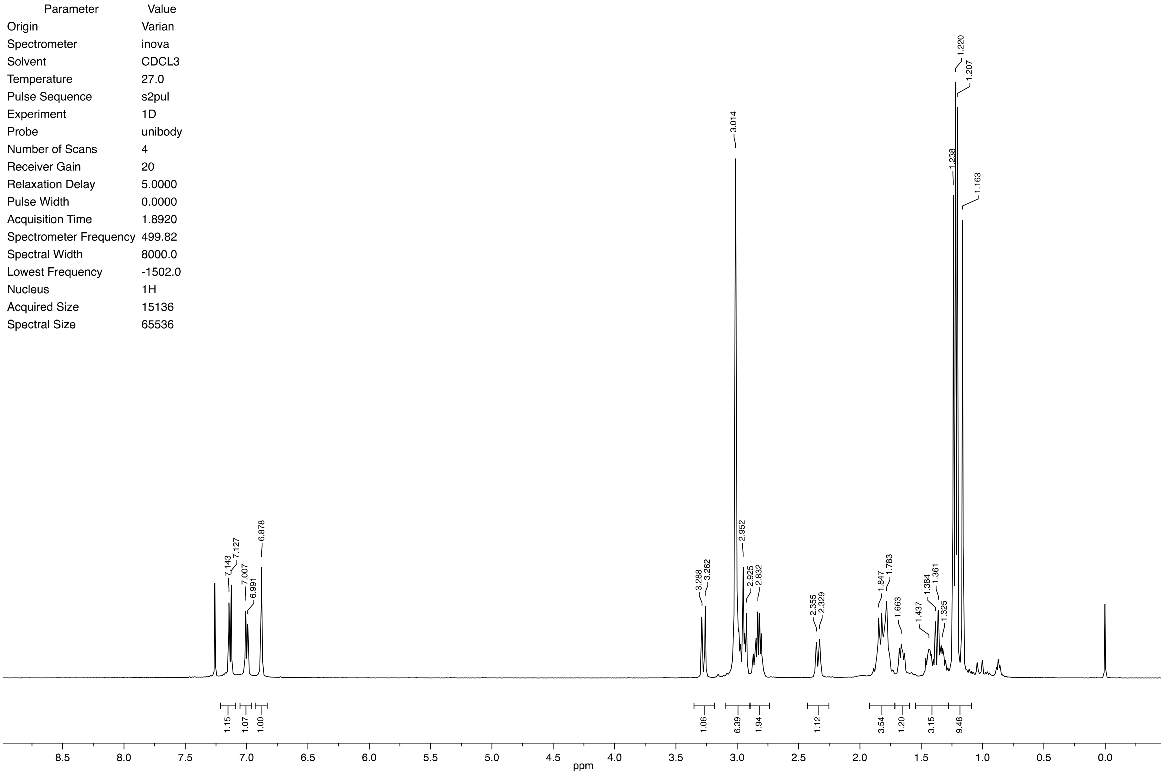

Figure S25. ^{1}H Spectra of Compound **6**.

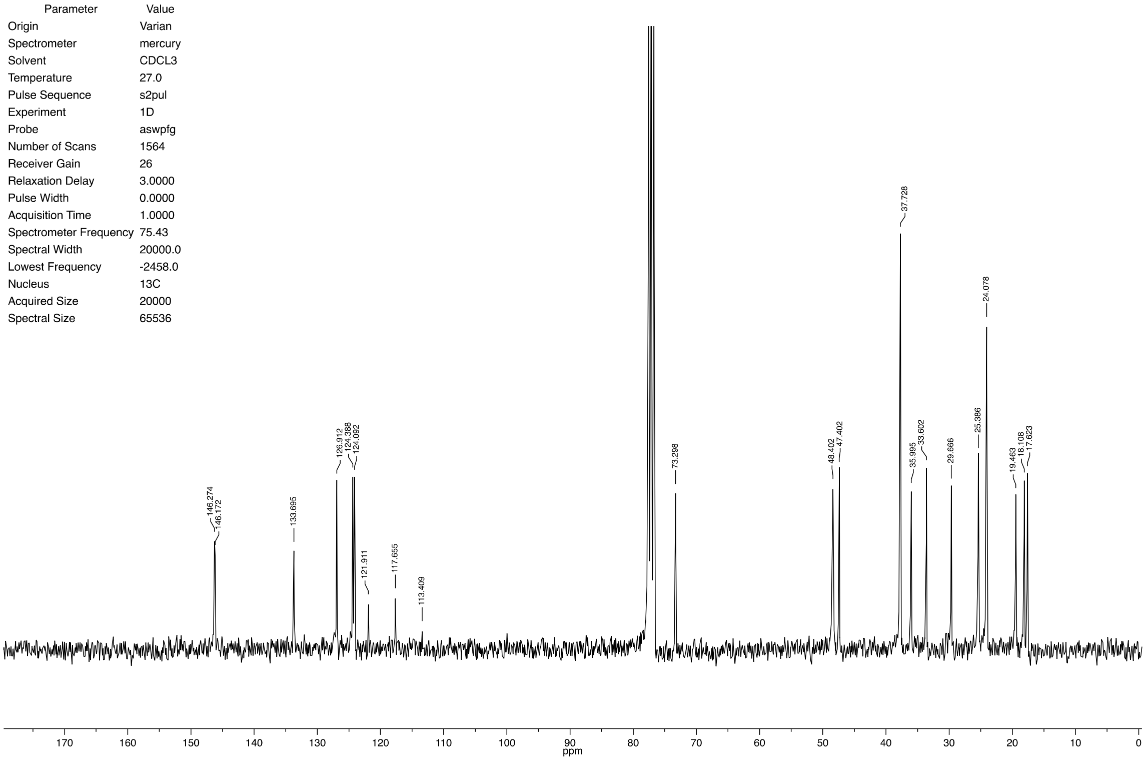

Figure S26. ^{13}C Spectra of Compound **6**.

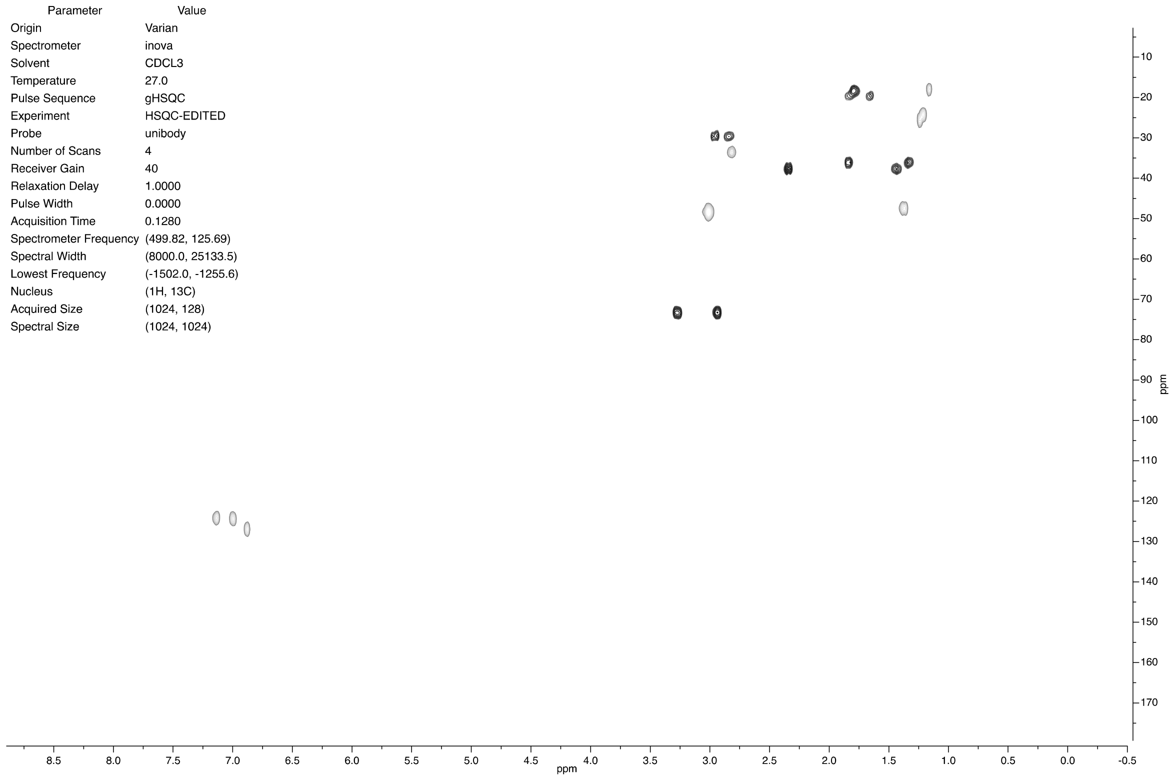

Figure S27. HSQC Spectra of Compound **6**.

7.3. ¹H, ¹³C and HSQC Spectra of Compound 7a

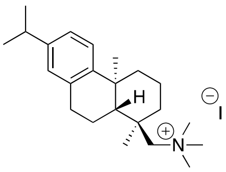

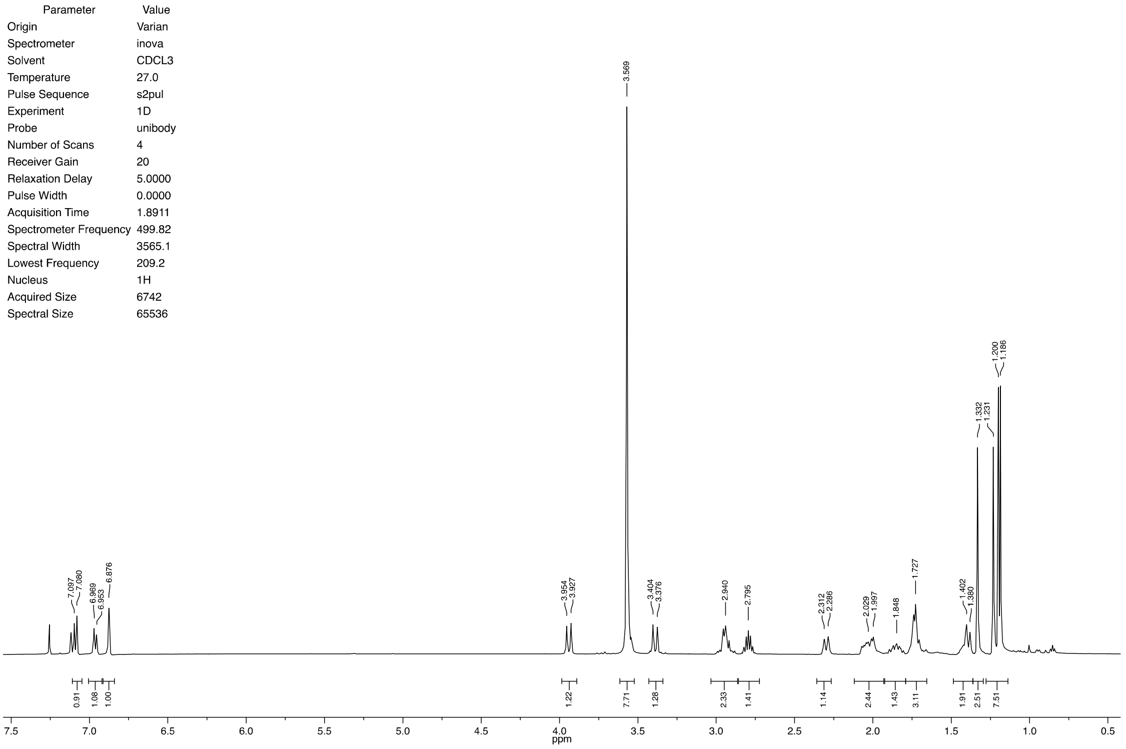

Figure S28. ¹H Spectra of Compound **7a**.

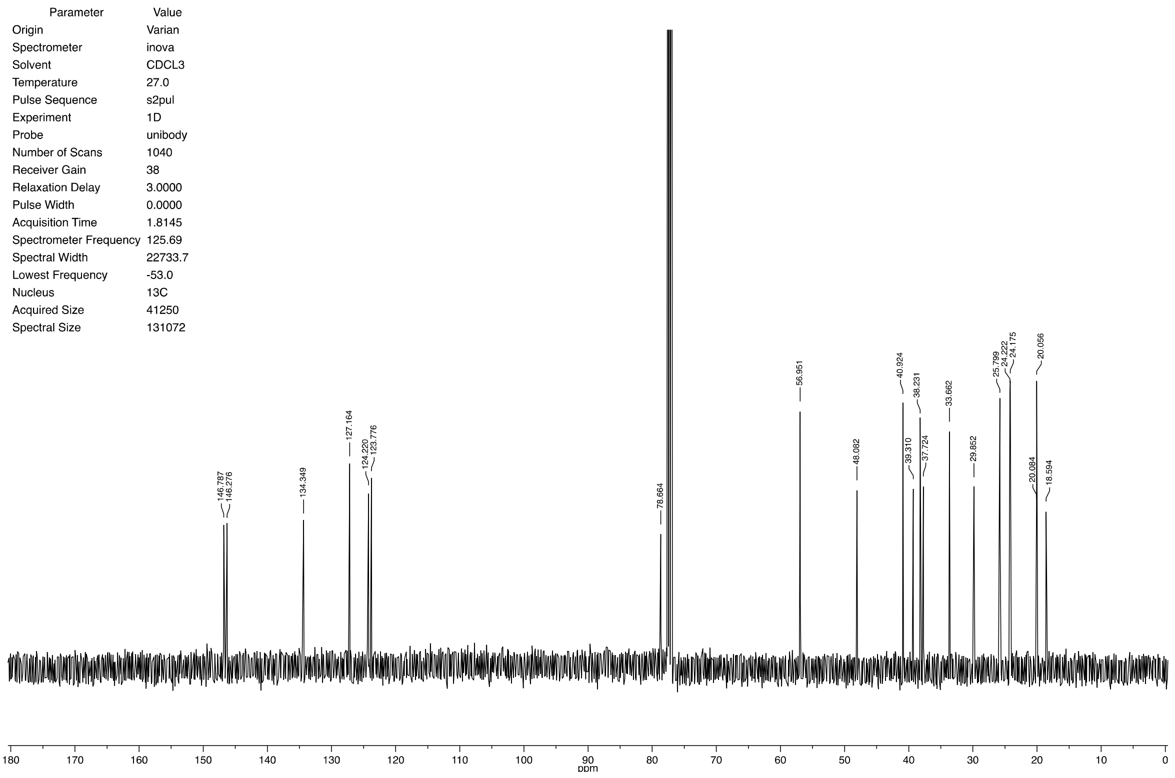

Figure S29. ^{13}C Spectra of Compound **7a**.

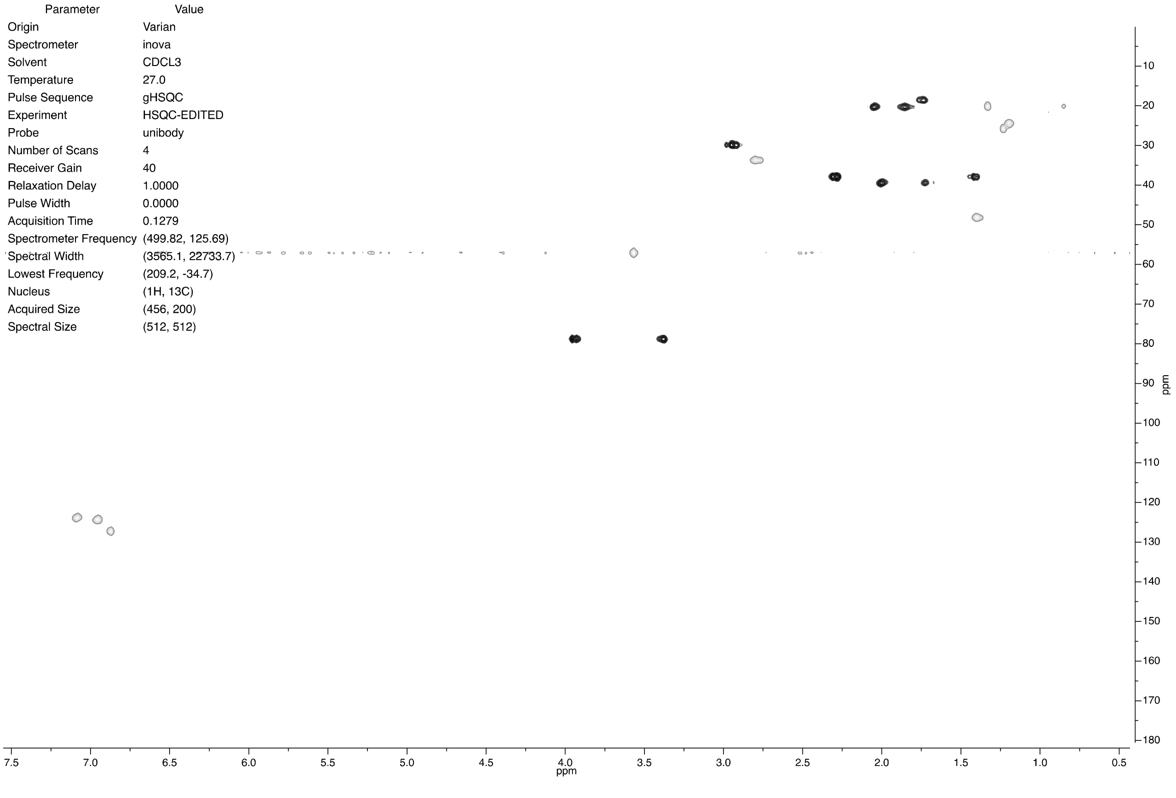

Figure S30. HSQC Spectra of Compound **7a**.

7.4. ¹H, ¹³C and HSQC Spectra of Compound **7b**

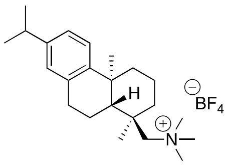

Parameter	Value
Origin	Varian
Spectrometer	inova
Solvent	CDCL3+3% CD3OD
Temperature	27.0
Pulse Sequence	s2pul
Experiment	1D
Probe	unibody
Number of Scans	6
Receiver Gain	20
Relaxation Delay	5.0000
Pulse Width	0.0000
Acquisition Time	1.8920
Spectrometer Frequency	499.82
Spectral Width	8000.0
Lowest Frequency	-1486.8
Nucleus	1H
Acquired Size	15136
Spectral Size	65536

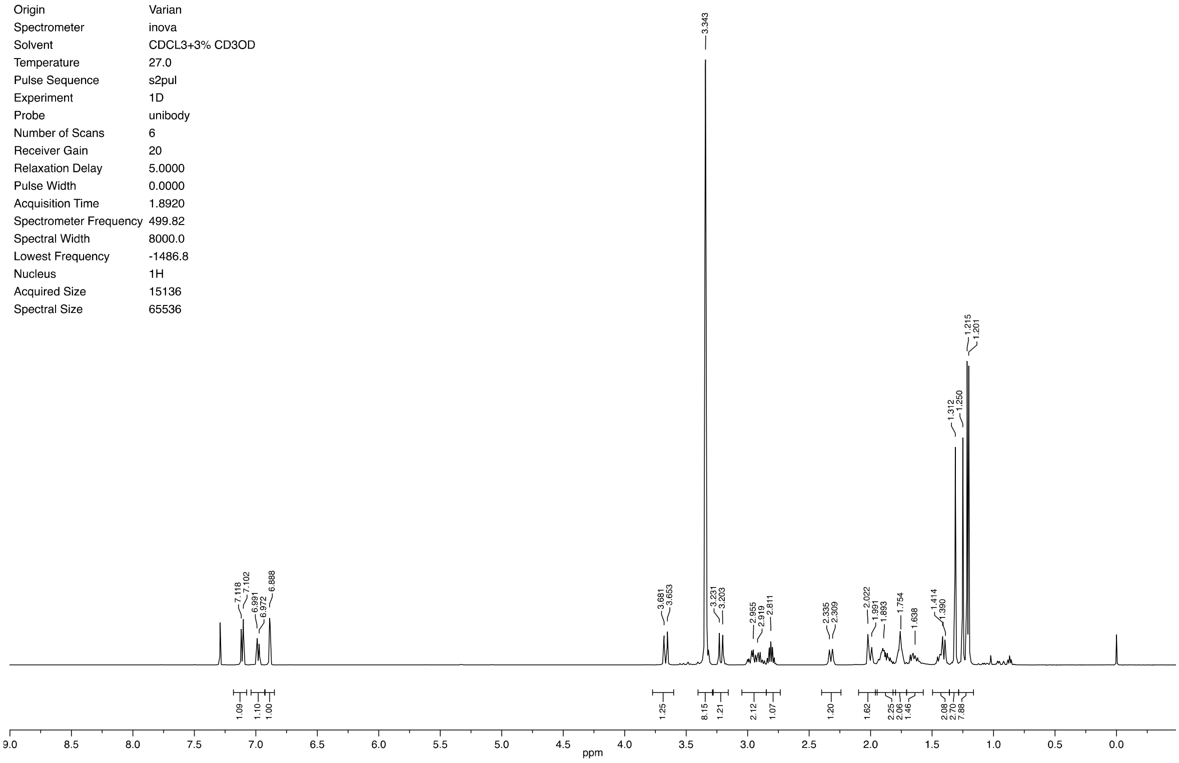

Figure S31. ¹H Spectra of Compound **7b**.

Parameter	Value
Origin	Varian
Spectrometer	inova
Solvent	CDCL3+3% CD3OD
Temperature	27.0
Pulse Sequence	s2pul
Experiment	1D
Probe	unibody
Number of Scans	944
Receiver Gain	38
Relaxation Delay	3.0000
Pulse Width	0.0000
Acquisition Time	1.8150
Spectrometer Frequency	125.69
Spectral Width	31446.5
Lowest Frequency	-2877.6
Nucleus	13C
Acquired Size	57076
Spectral Size	131072

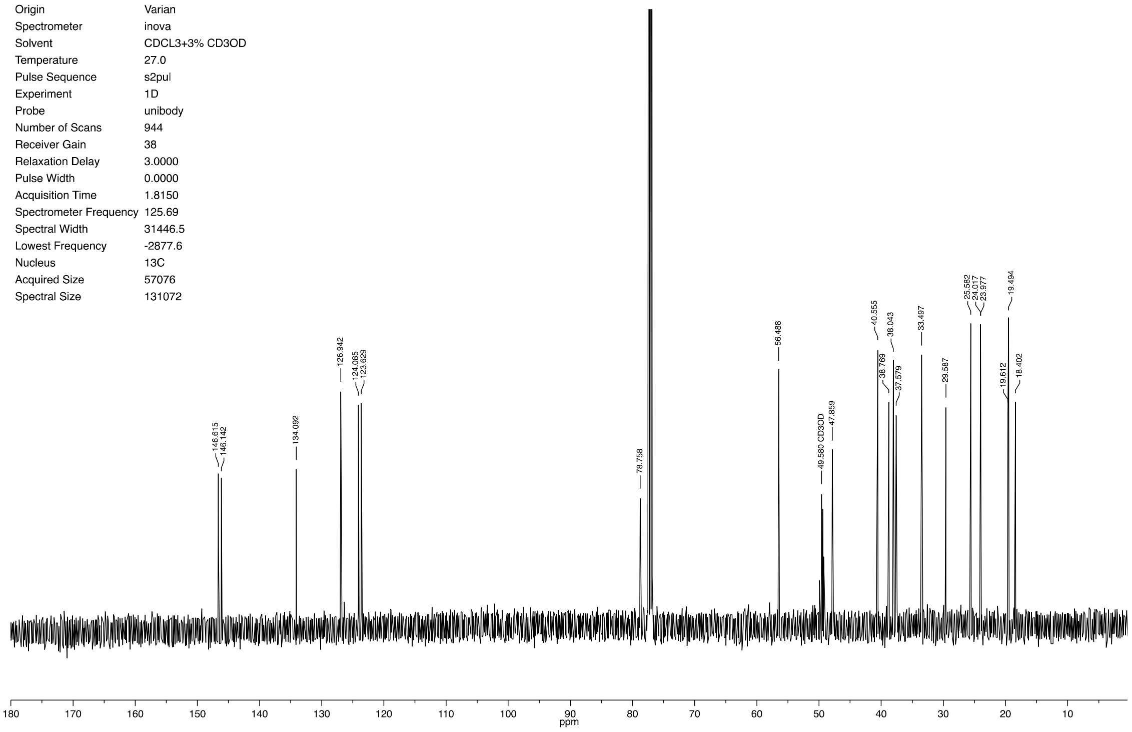

Figure S32. ¹³C Spectra of Compound **7a**.

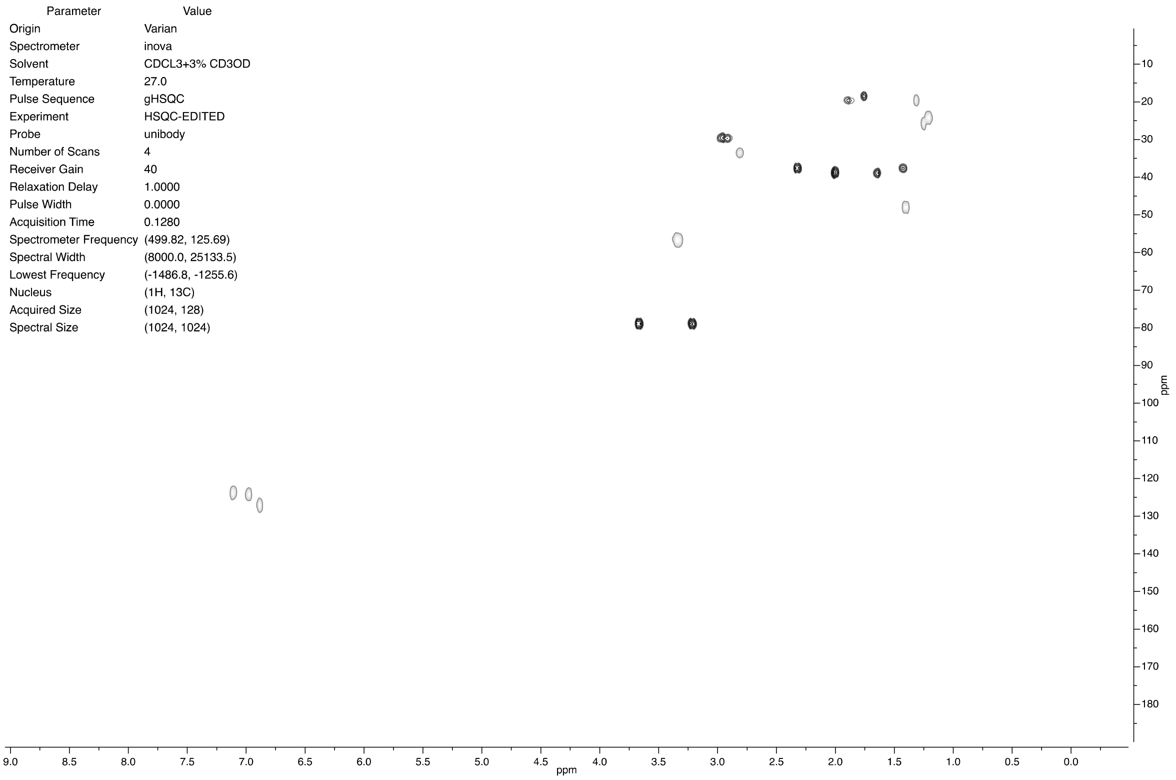

Figure S33. HSQC Spectra of Compound **7a**.

7.5. ^{1}H, ^{13}C and HSQC Spectra of Compound **7c**

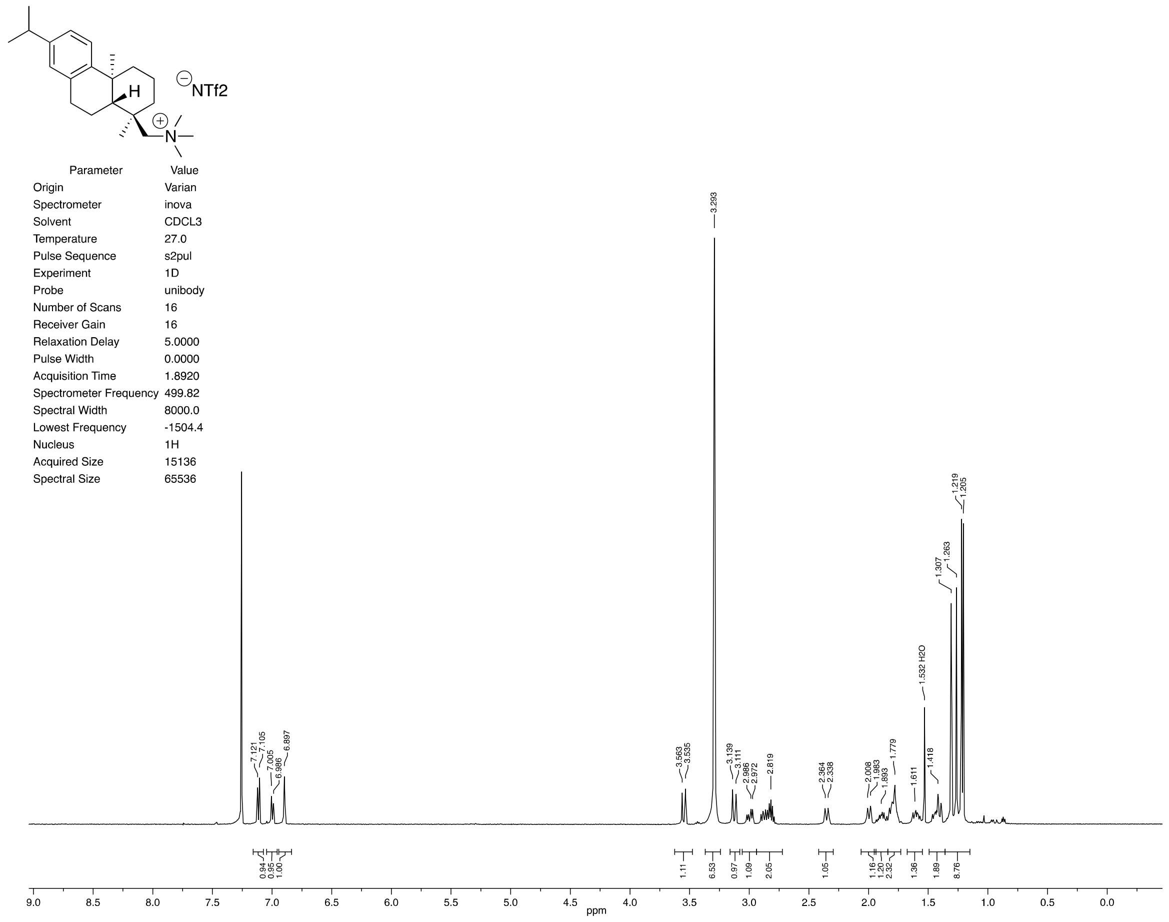

Figure S34. [1]H Spectra of Compound **7c**.

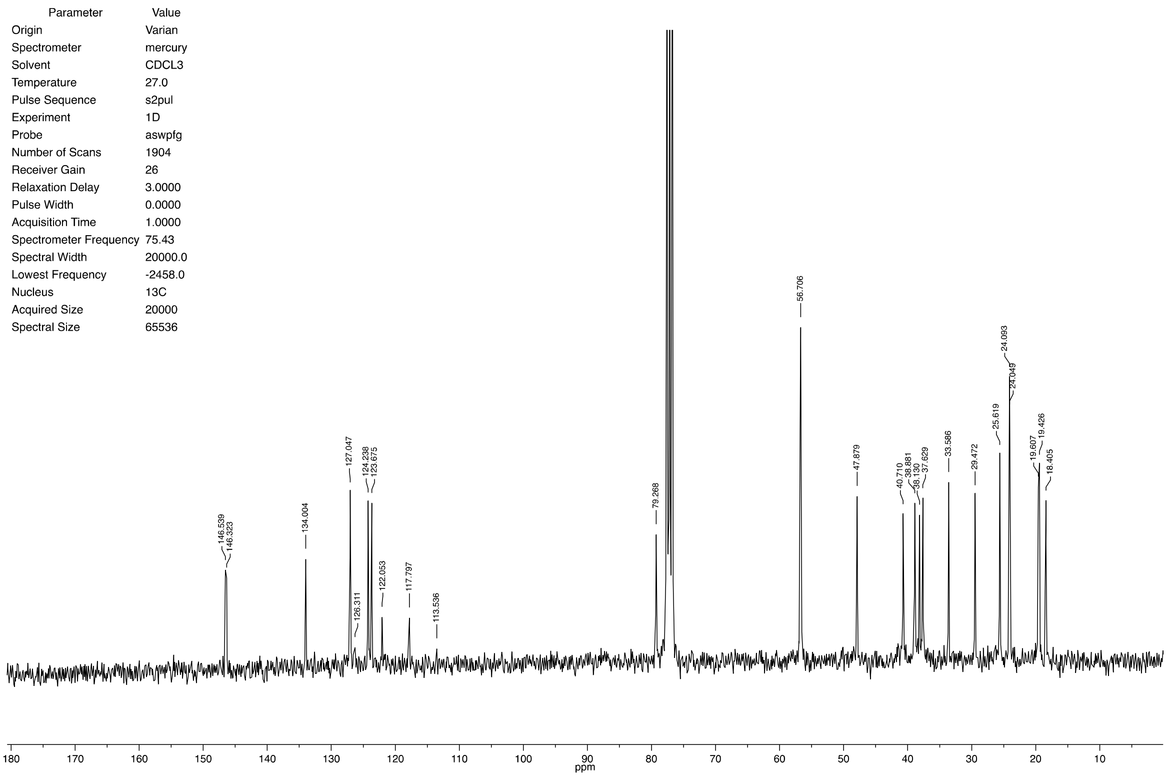

Figure S35. ^{13}C Spectra of Compound **7c**.

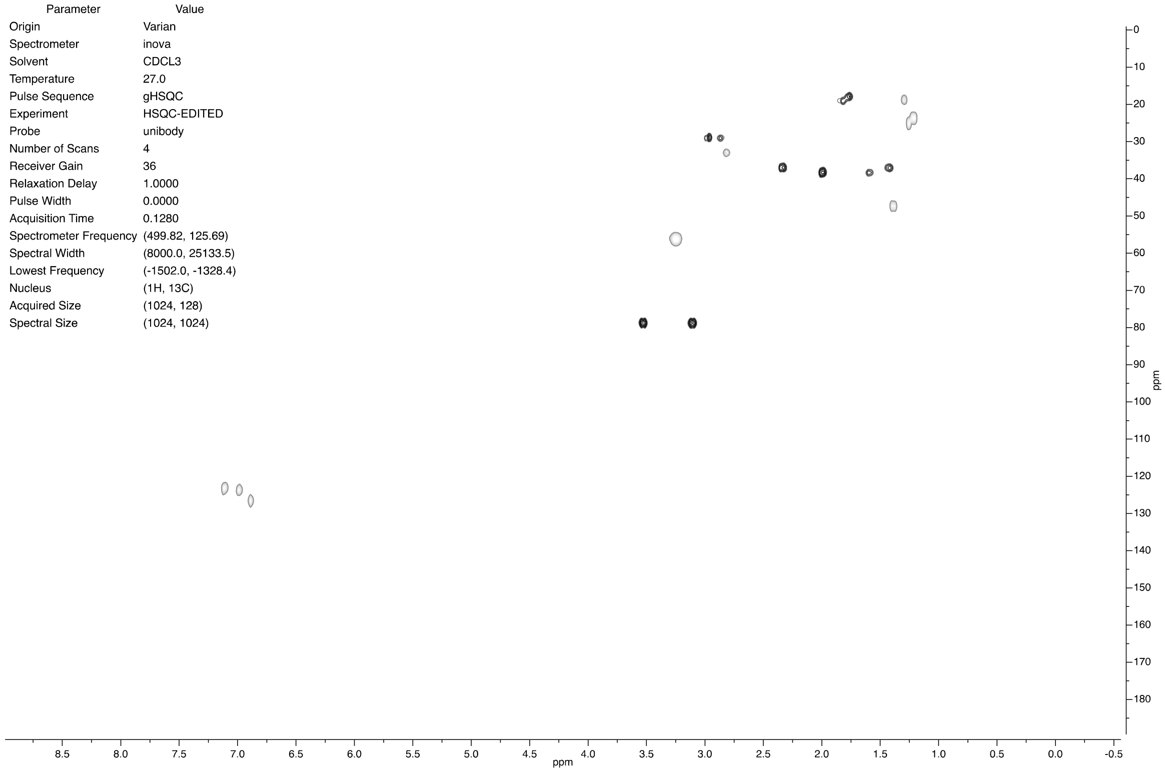

Figure S36. HSQC Spectra of Compound **7c**.

7.6. ^{1}H, ^{13}C and HSQC Spectra of Compound **8a**

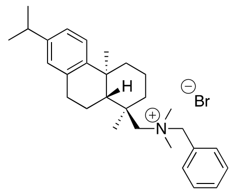

Parameter	Value
Origin	Varian
Spectrometer	inova
Solvent	CDCL3
Temperature	27.0
Pulse Sequence	s2pul
Experiment	1D
Probe	unibody
Number of Scans	4
Receiver Gain	20
Relaxation Delay	5.0000
Pulse Width	0.0000
Acquisition Time	1.8964
Spectrometer Frequency	499.82
Spectral Width	4278.1
Lowest Frequency	-222.9
Nucleus	1H
Acquired Size	8113
Spectral Size	65536

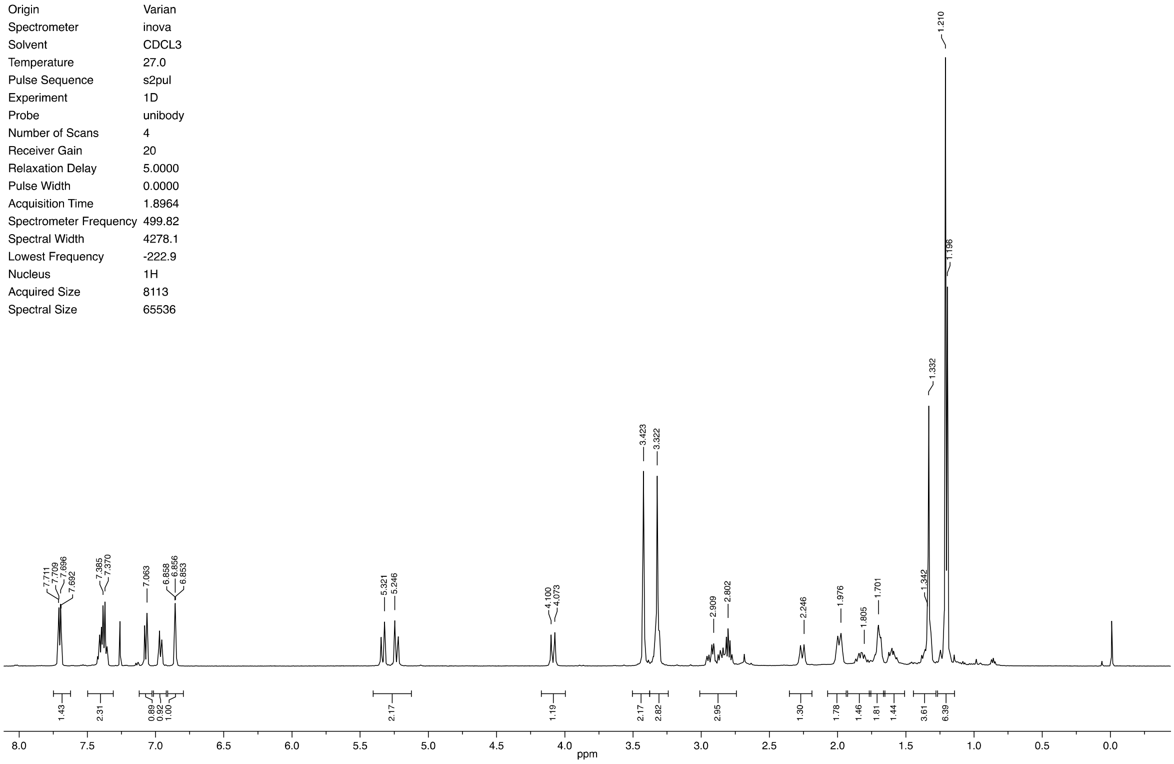

Figure S37. ^{1}H Spectra of Compound **8a**.

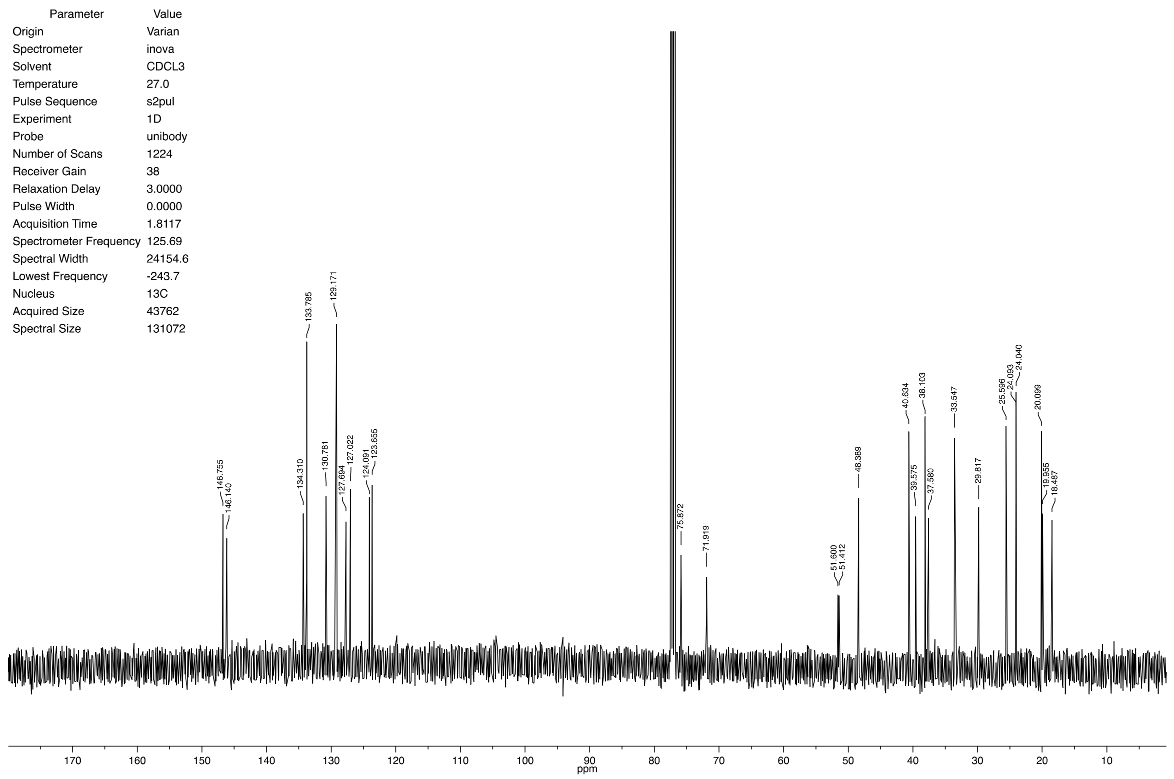

Figure S38. ^{13}C Spectra of Compound **8a**.

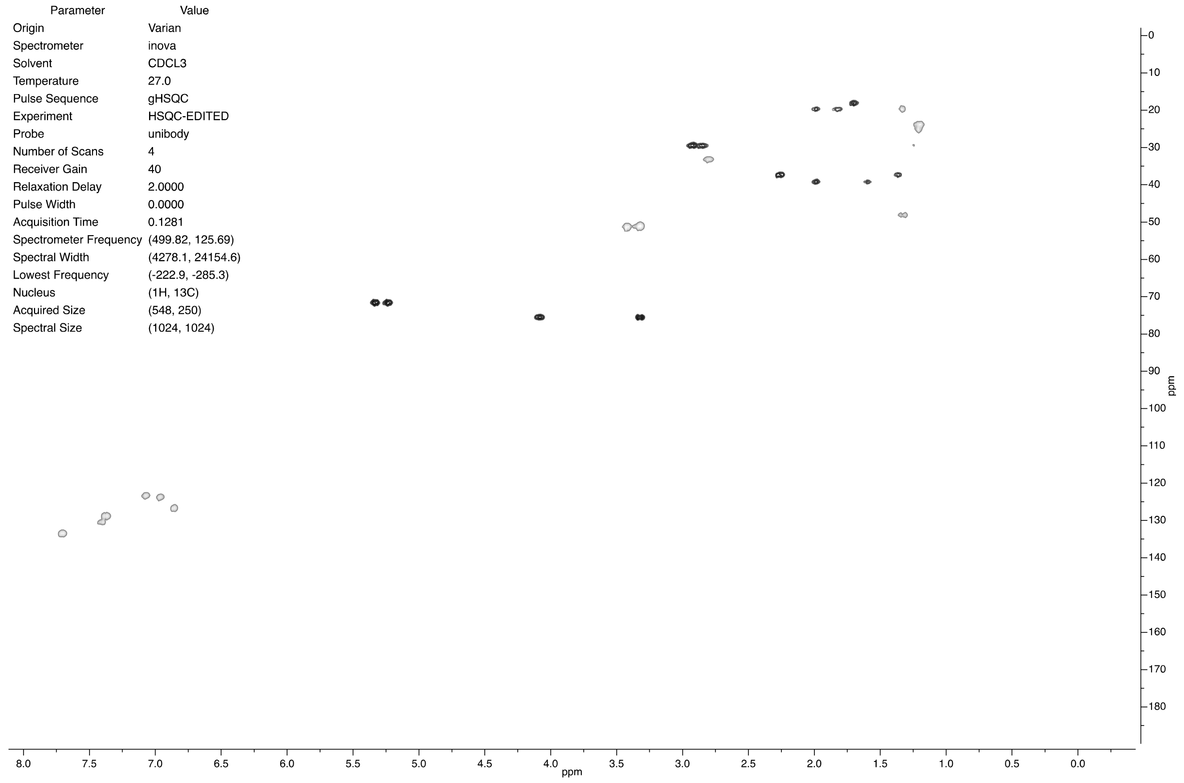

Figure S39. HSQC Spectra of Compound **8a**.

7.7. ^1H, ^{13}C and HSQC Spectra of Compound **8b**

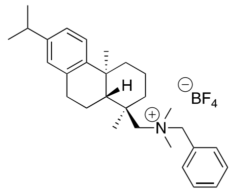

Parameter	Value
Origin	Varian
Spectrometer	inova
Solvent	CDCL3
Temperature	27.0
Pulse Sequence	s2pul
Experiment	1D
Probe	unibody
Number of Scans	8
Receiver Gain	16
Relaxation Delay	5.0000
Pulse Width	0.0000
Acquisition Time	1.8970
Spectrometer Frequency	499.82
Spectral Width	6712.5
Lowest Frequency	-711.3
Nucleus	1H
Acquired Size	12734
Spectral Size	65536

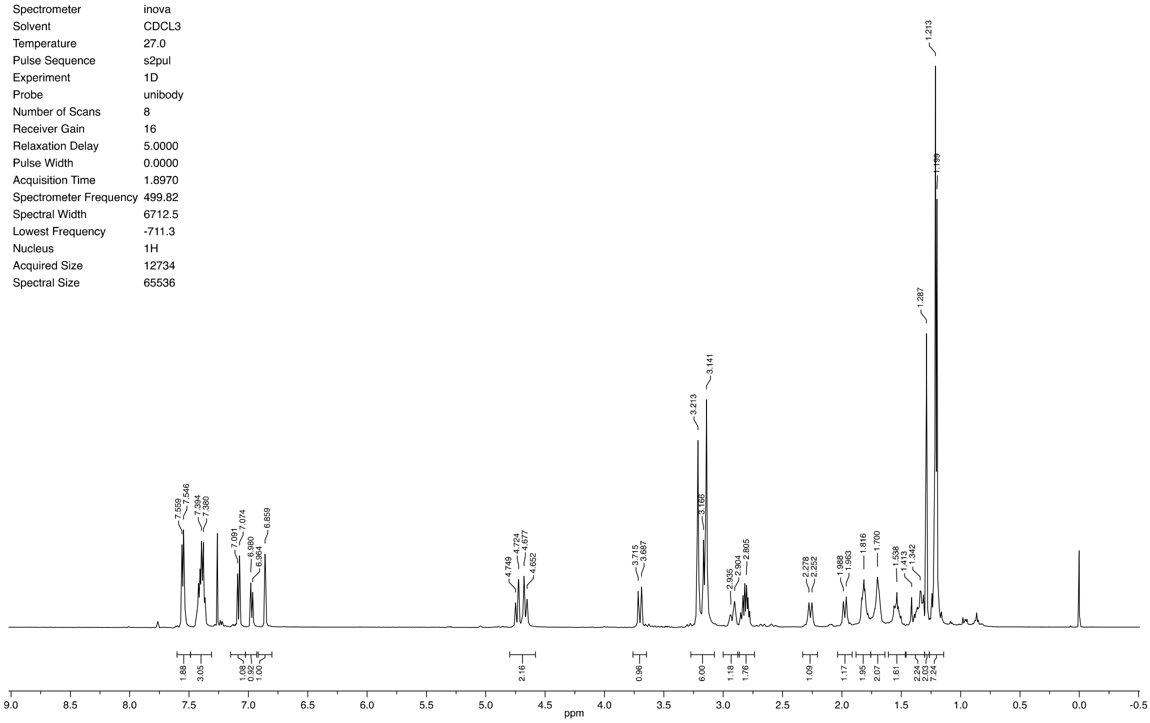

Figure S40. ^{1}H Spectra of Compound **8b**.

Parameter	Value
Origin	Varian
Spectrometer	mercury
Solvent	CDCL3
Temperature	27.0
Pulse Sequence	s2pul
Experiment	1D
Probe	aswpfg
Number of Scans	1632
Receiver Gain	26
Relaxation Delay	3.0000
Pulse Width	0.0000
Acquisition Time	1.0000
Spectrometer Frequency	75.43
Spectral Width	20000.0
Lowest Frequency	-2458.0
Nucleus	13C
Acquired Size	20000
Spectral Size	65536

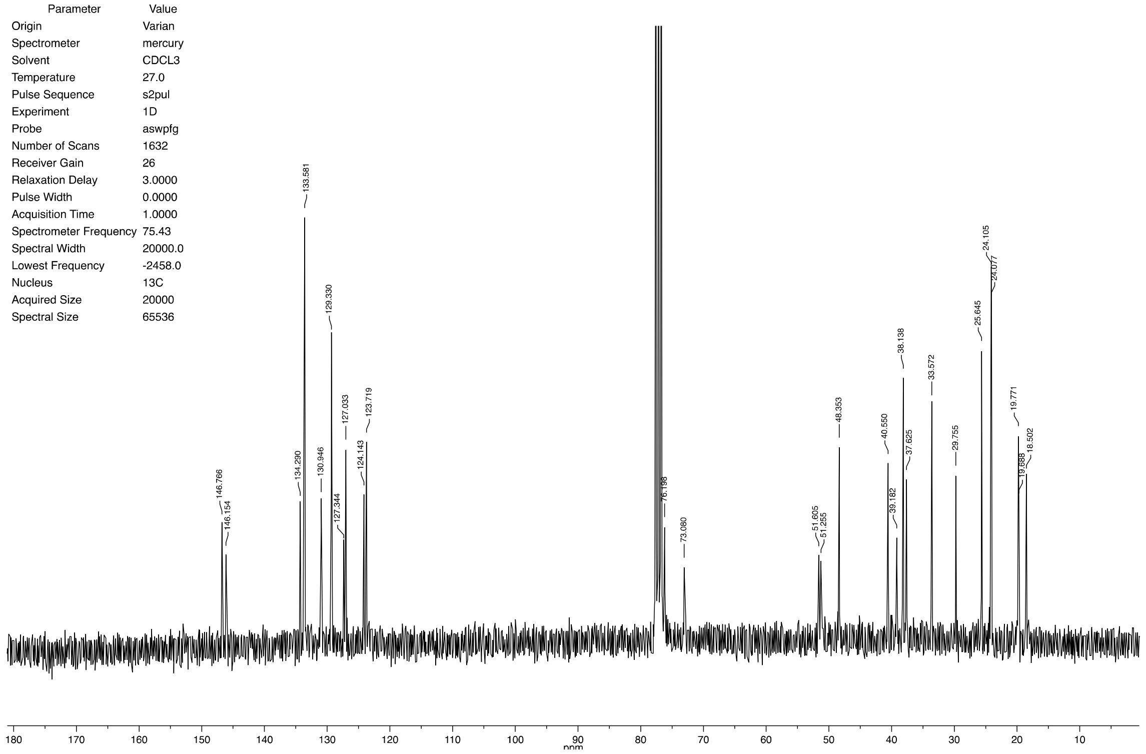

Figure S41. ^{13}C Spectra of Compound **8b**.

Parameter	Value
Origin	Varian
Spectrometer	inova
Solvent	CDCL3
Temperature	27.0
Pulse Sequence	gHSQC
Experiment	HSQC-EDITED
Probe	unibody
Number of Scans	4
Receiver Gain	36
Relaxation Delay	1.0000
Pulse Width	0.0000
Acquisition Time	0.1280
Spectrometer Frequency	(499.82, 125.69)
Spectral Width	(6712.5, 25133.5)
Lowest Frequency	(-711.3, -1255.6)
Nucleus	(1H, 13C)
Acquired Size	(859, 128)
Spectral Size	(1024, 1024)

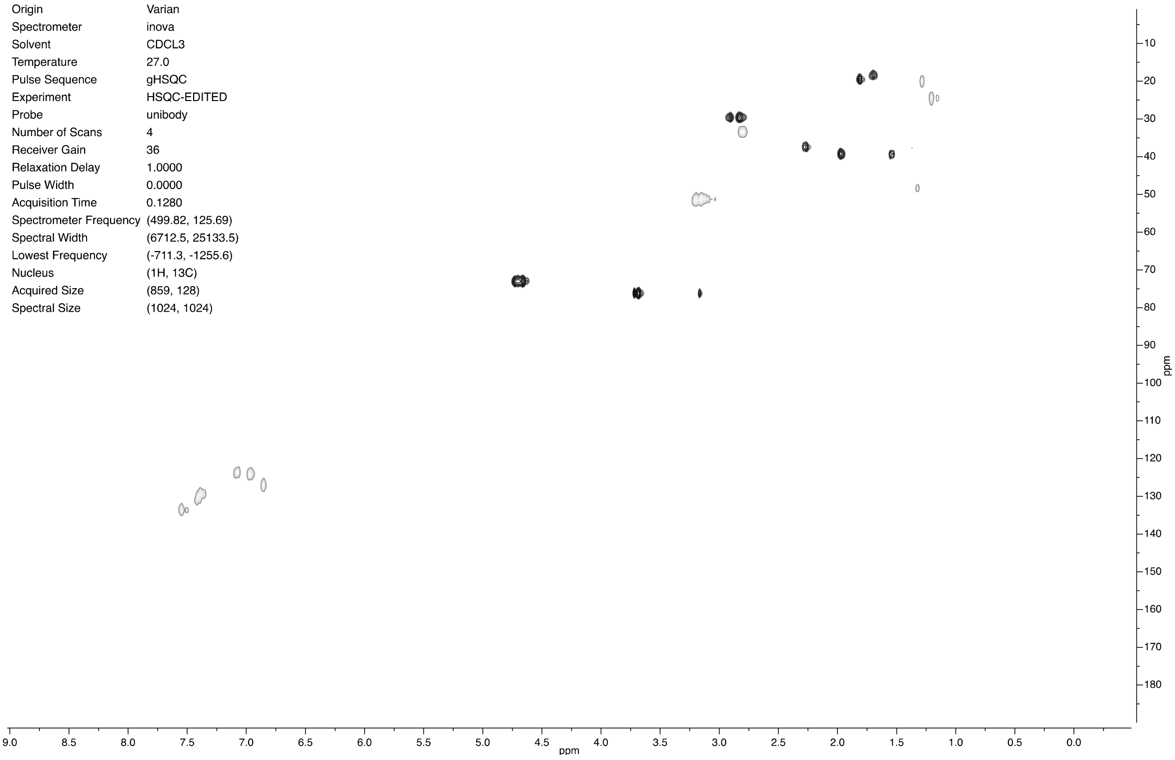

Figure S42. HSQC Spectra of Compound **8b**.

7.8. *^{1}H, ^{13}C and HSQC Spectra of Compound* **8c**

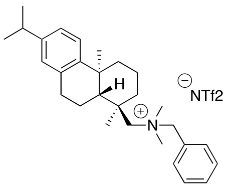

Parameter	Value
Origin	Varian
Spectrometer	inova
Solvent	CDCL3
Temperature	27.0
Pulse Sequence	s2pul
Experiment	1D
Probe	unibody
Number of Scans	8
Receiver Gain	16
Relaxation Delay	5.0000
Pulse Width	0.0000
Acquisition Time	1.8970
Spectrometer Frequency	499.82
Spectral Width	6712.5
Lowest Frequency	-711.3
Nucleus	1H
Acquired Size	12734
Spectral Size	65536

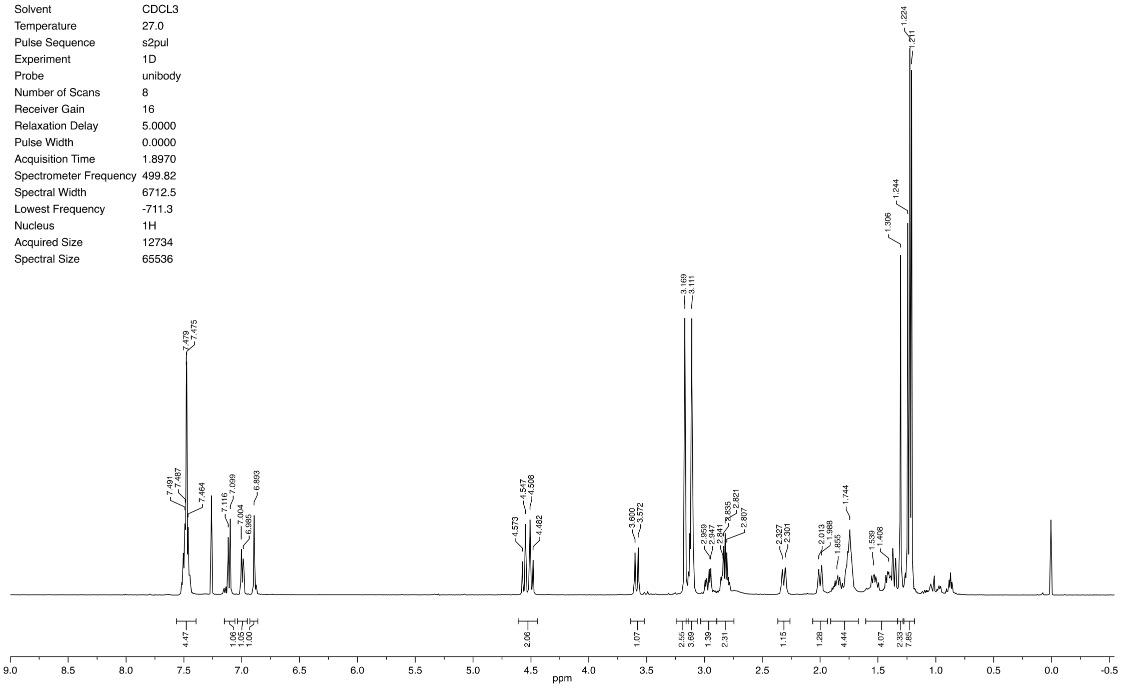

Figure S43. [1]H Spectra of Compound **8c**.

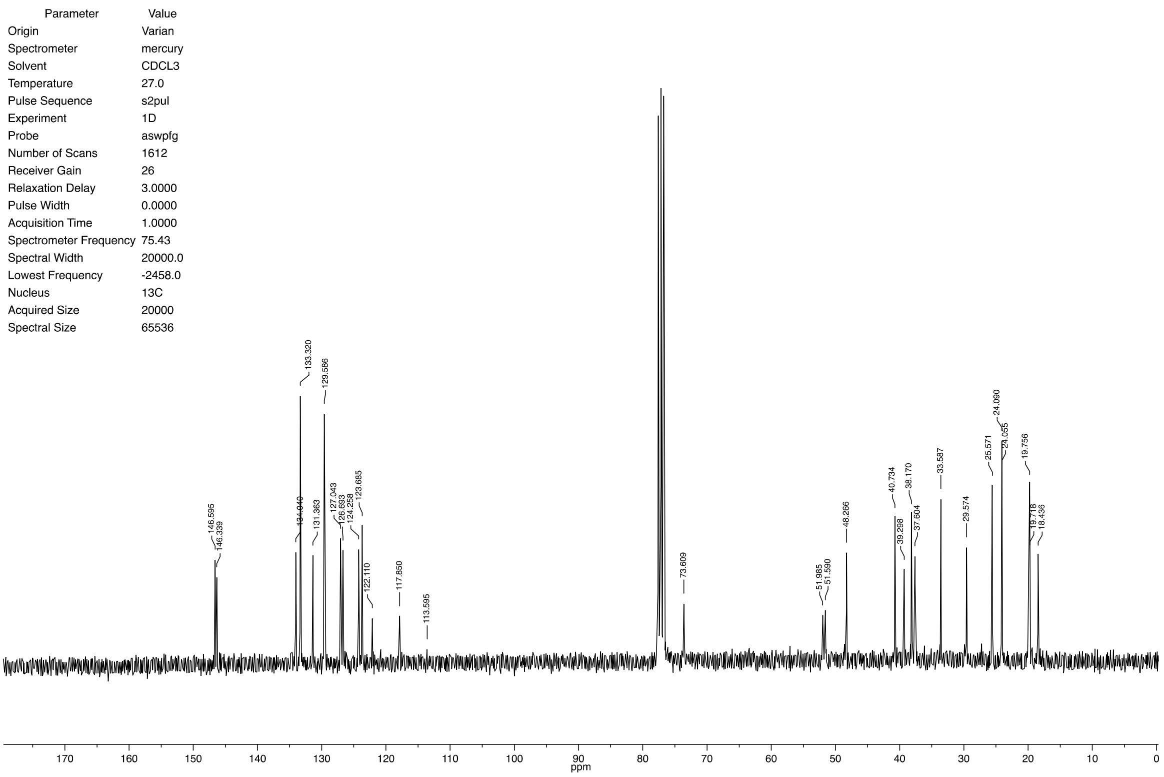

Figure S44. ^{13}C Spectra of Compound **8c**.

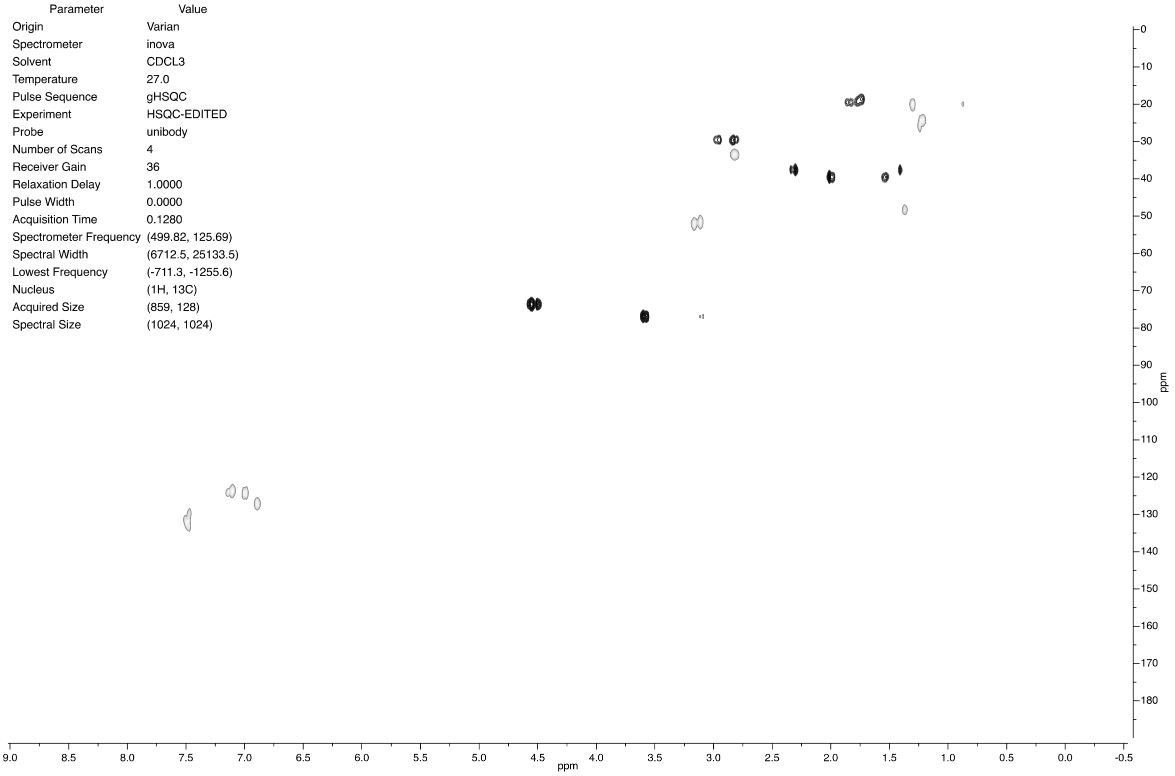

Figure S45. HSQC Spectra of Compound **8c**.

7.9. ¹H, ¹³C and HSQC Spectra of Compound **9a**

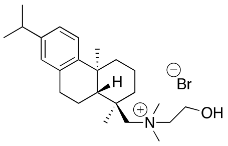

Parameter	Value
Origin	Varian
Spectrometer	inova
Solvent	CD3OD
Temperature	27.0
Pulse Sequence	s2pul
Experiment	1D
Probe	unibody
Number of Scans	4
Receiver Gain	16
Relaxation Delay	5.0000
Pulse Width	0.0000
Acquisition Time	1.8909
Spectrometer Frequency	499.82
Spectral Width	3351.8
Lowest Frequency	600.3
Nucleus	1H
Acquired Size	6338
Spectral Size	65536

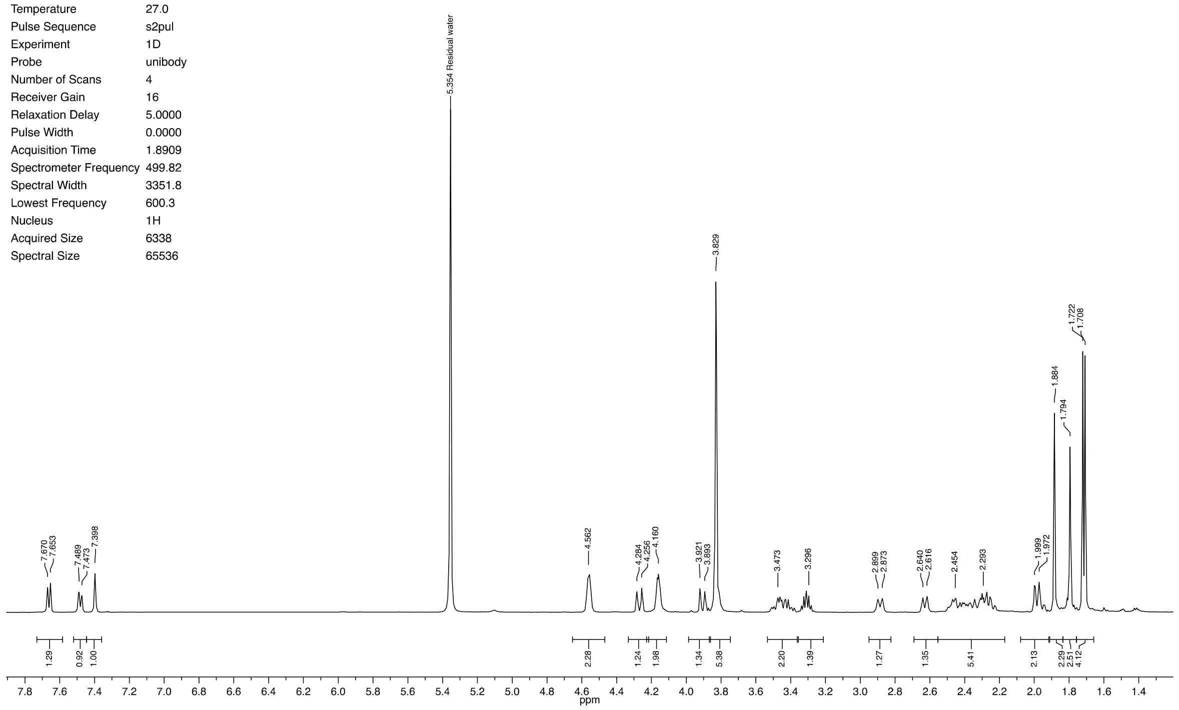

Figure S46. ¹H Spectra of Compound **9a**.

Parameter	Value
Origin	Varian
Spectrometer	inova
Solvent	CD3OD
Temperature	27.0
Pulse Sequence	s2pul
Experiment	1D
Probe	unibody
Number of Scans	1000
Receiver Gain	38
Relaxation Delay	5.0000
Pulse Width	0.0000
Acquisition Time	1.8150
Spectrometer Frequency	125.69
Spectral Width	22844.1
Lowest Frequency	-84.2
Nucleus	13C
Acquired Size	41462
Spectral Size	131072

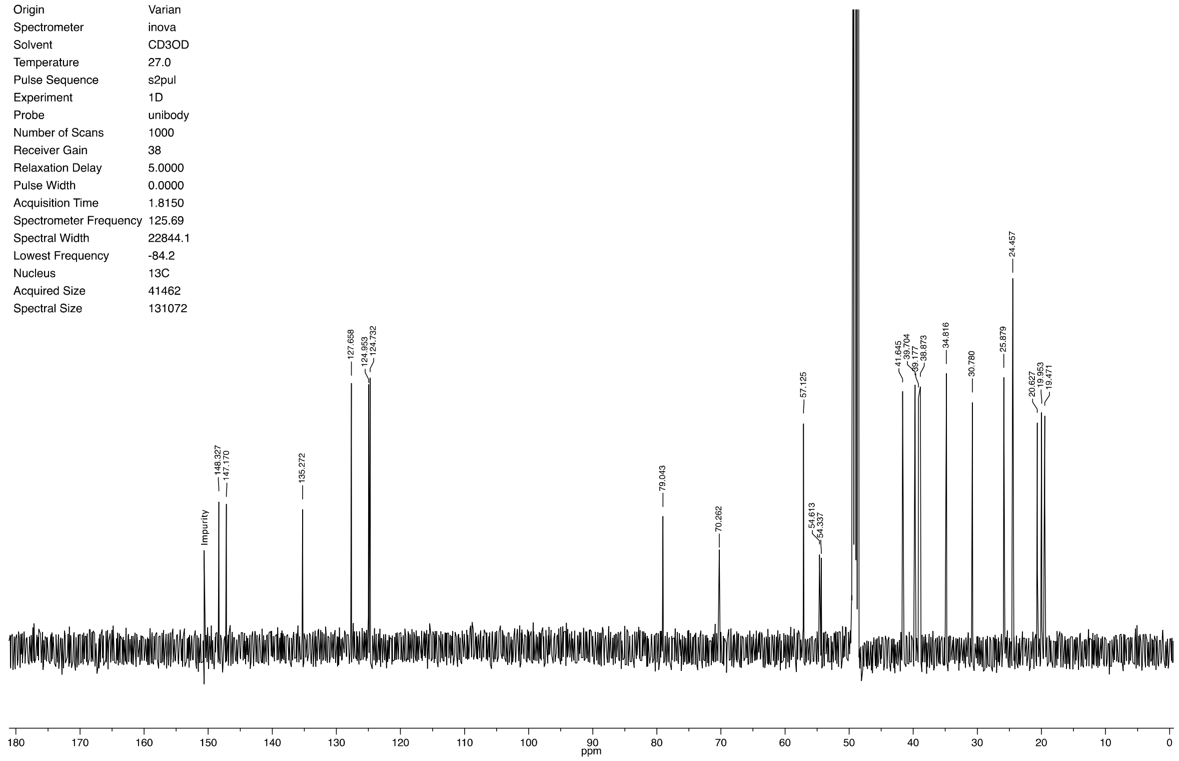

Figure S47. ¹³C Spectra of Compound **9a**.

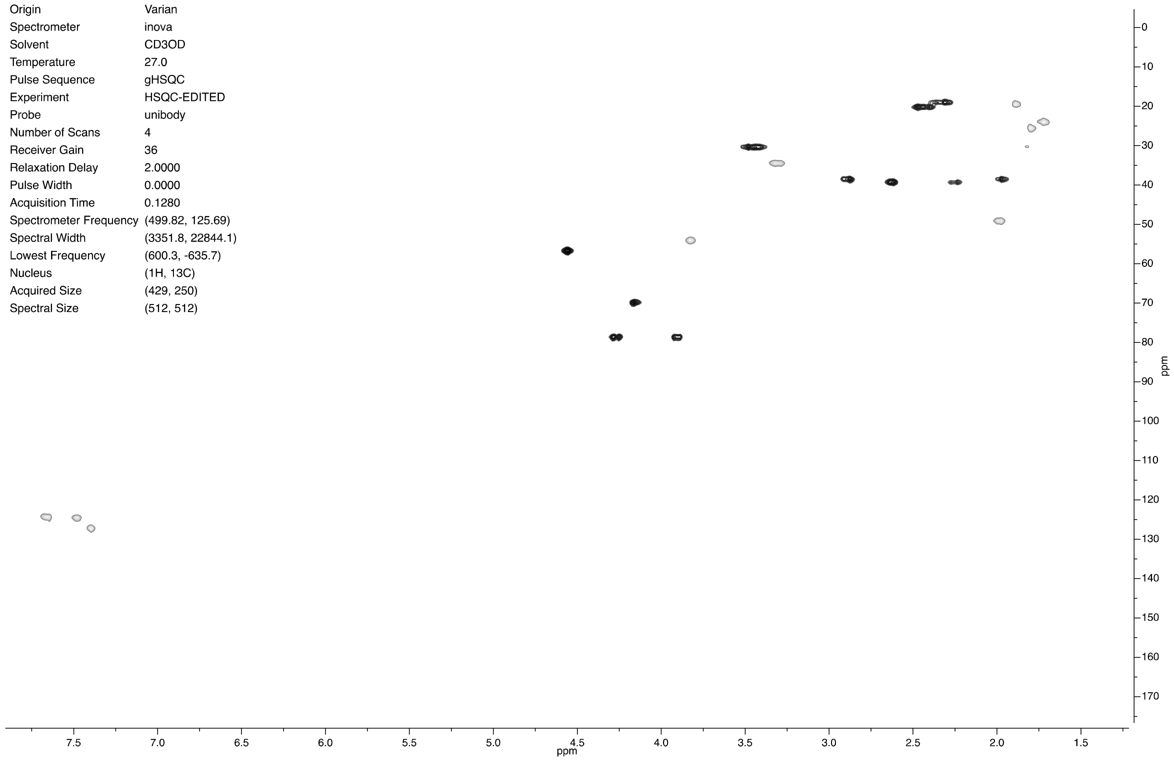

Parameter	Value
Origin	Varian
Spectrometer	inova
Solvent	CD3OD
Temperature	27.0
Pulse Sequence	gHSQC
Experiment	HSQC-EDITED
Probe	unibody
Number of Scans	4
Receiver Gain	36
Relaxation Delay	2.0000
Pulse Width	0.0000
Acquisition Time	0.1280
Spectrometer Frequency	(499.82, 125.69)
Spectral Width	(3351.8, 22844.1)
Lowest Frequency	(600.3, -635.7)
Nucleus	(1H, 13C)
Acquired Size	(429, 250)
Spectral Size	(512, 512)

Figure S48. HSQC Spectra of Compound **9a**.

7.10. ^{1}H, ^{13}C and HSQC Spectra of Compound **9b**

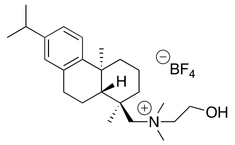

Parameter	Value
Origin	Varian
Spectrometer	inova
Solvent	CDCL3
Temperature	27.0
Pulse Sequence	s2pul
Experiment	1D
Probe	unibody
Number of Scans	16
Receiver Gain	16
Relaxation Delay	5.0000
Pulse Width	0.0000
Acquisition Time	1.8920
Spectrometer Frequency	499.82
Spectral Width	8000.0
Lowest Frequency	-1502.4
Nucleus	1H
Acquired Size	15136
Spectral Size	65536

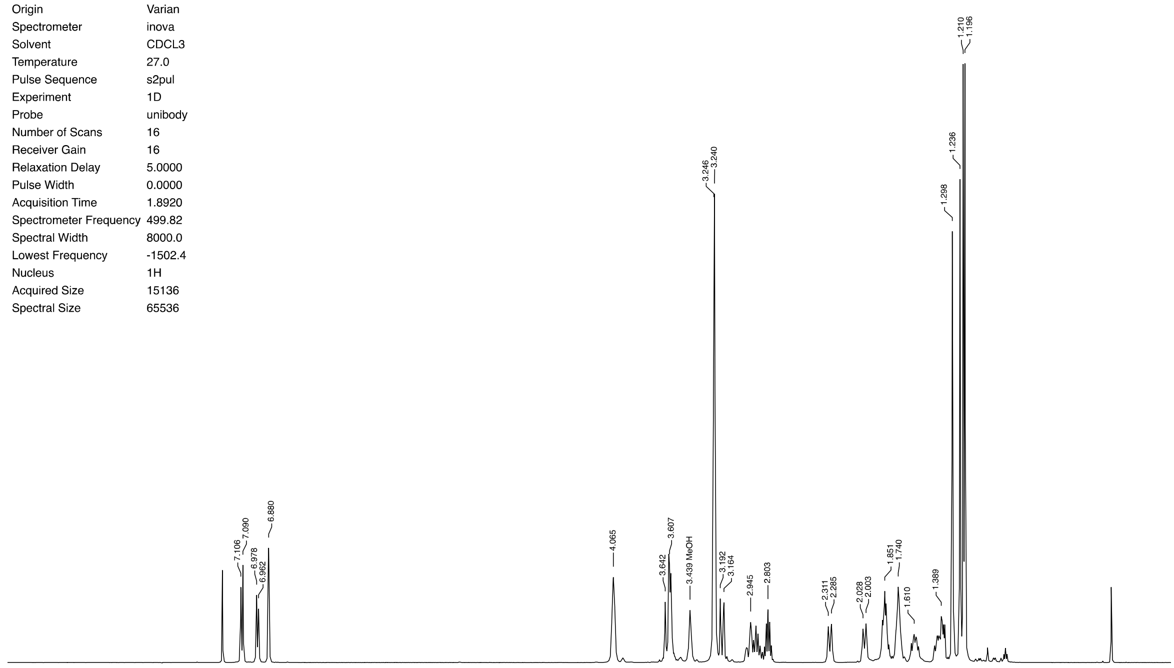

Figure S49. [1]H Spectra of Compound **9b**.

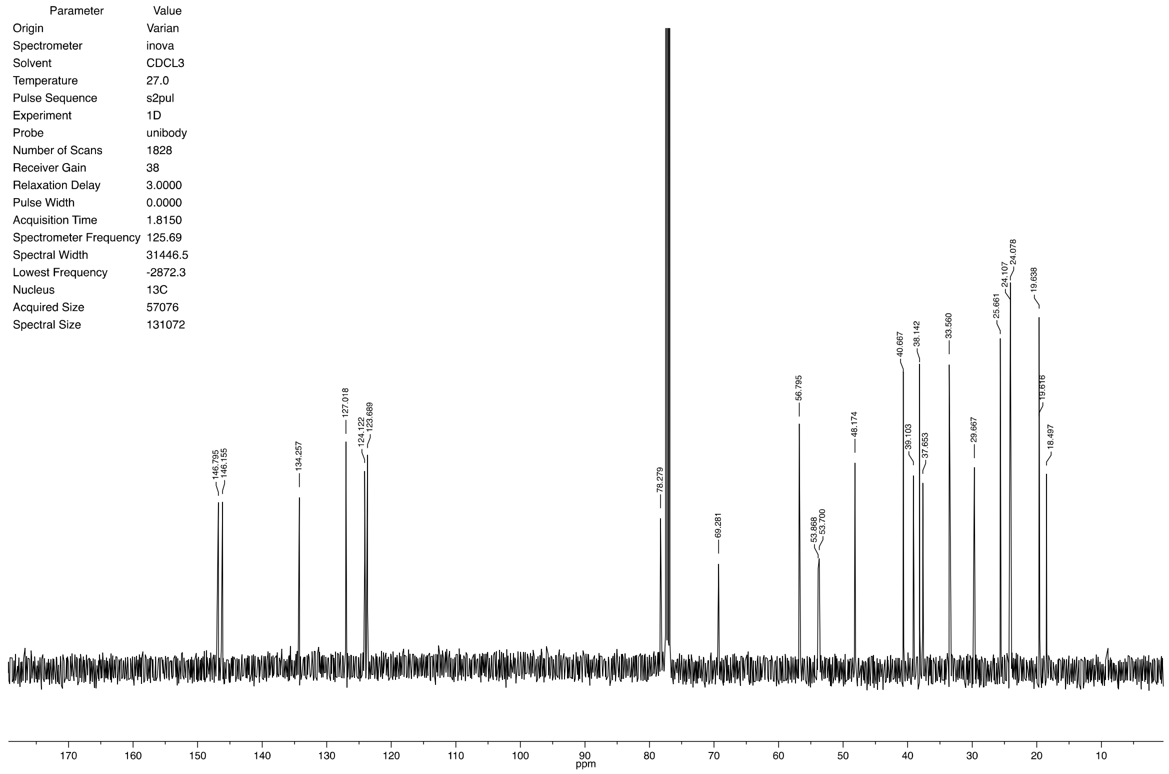

Figure S50. ^{13}C Spectra of Compound **9b**.

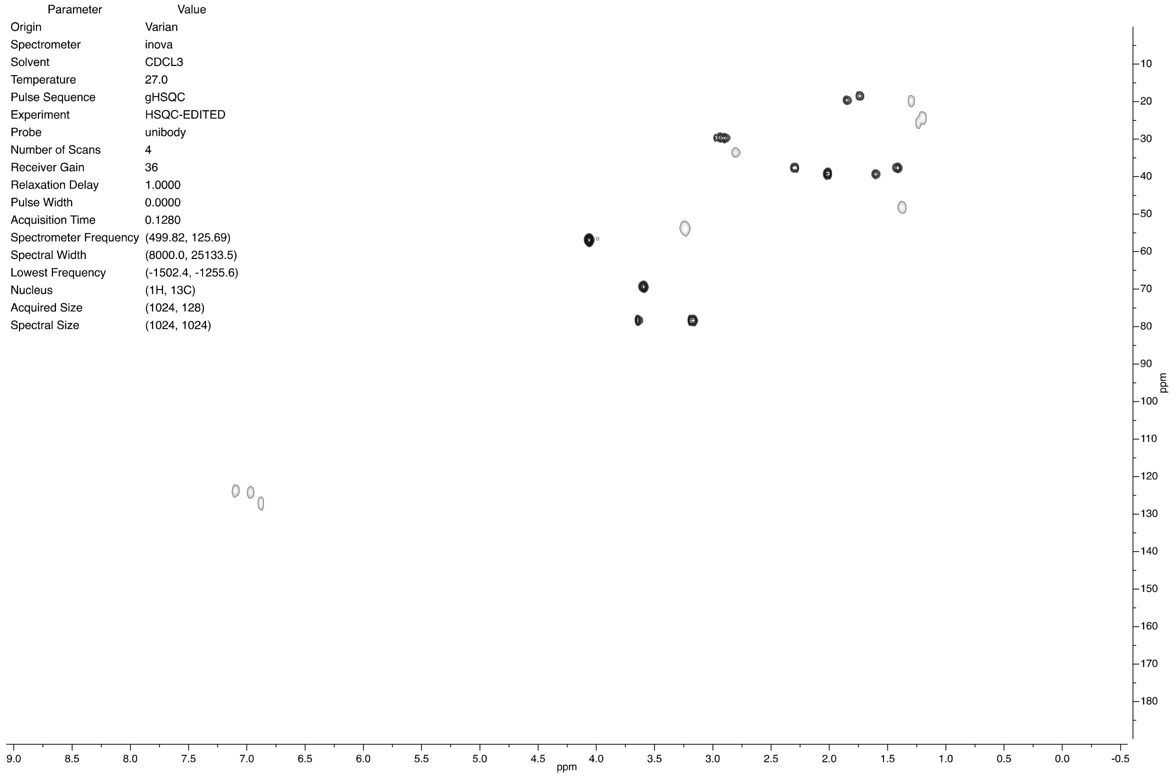

Figure S51. HSQC Spectra of Compound **9b**.

7.11. ^{1}H, ^{13}C and HSQC Spectra of Compound **9c**

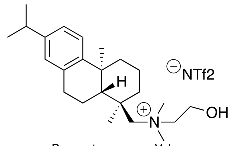

Parameter	Value
Origin	Varian
Spectrometer	inova
Solvent	CDCL3
Temperature	27.0
Pulse Sequence	s2pul
Experiment	1D
Probe	unibody
Number of Scans	32
Receiver Gain	20
Relaxation Delay	5.0000
Pulse Width	0.0000
Acquisition Time	1.8920
Spectrometer Frequency	499.82
Spectral Width	8000.0
Lowest Frequency	-1502.9
Nucleus	1H
Acquired Size	15136
Spectral Size	65536

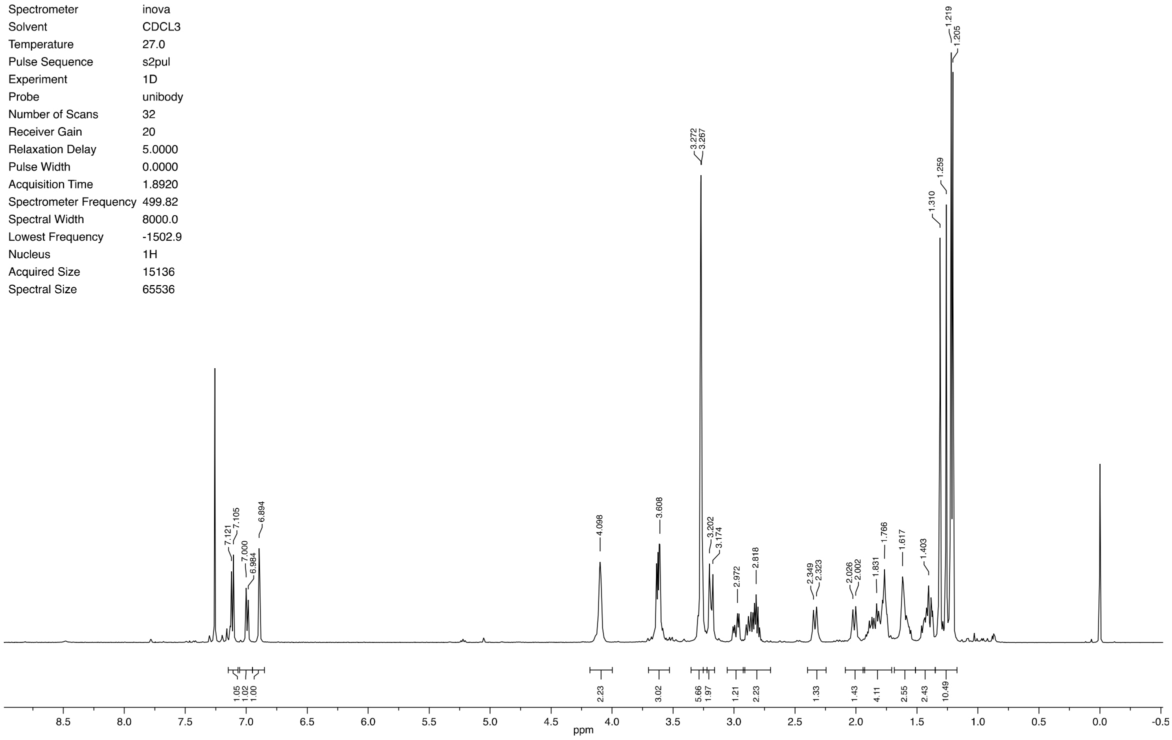

Figure S52. [1]H Spectra of Compound **9c**.

Parameter	Value
Origin	Varian
Spectrometer	inova
Solvent	CDCL3
Temperature	27.0
Pulse Sequence	s2pul
Experiment	1D
Probe	unibody
Number of Scans	1140
Receiver Gain	38
Relaxation Delay	3.0000
Pulse Width	0.0000
Acquisition Time	1.8150
Spectrometer Frequency	125.69
Spectral Width	31446.5
Lowest Frequency	-2871.4
Nucleus	13C
Acquired Size	57076
Spectral Size	131072

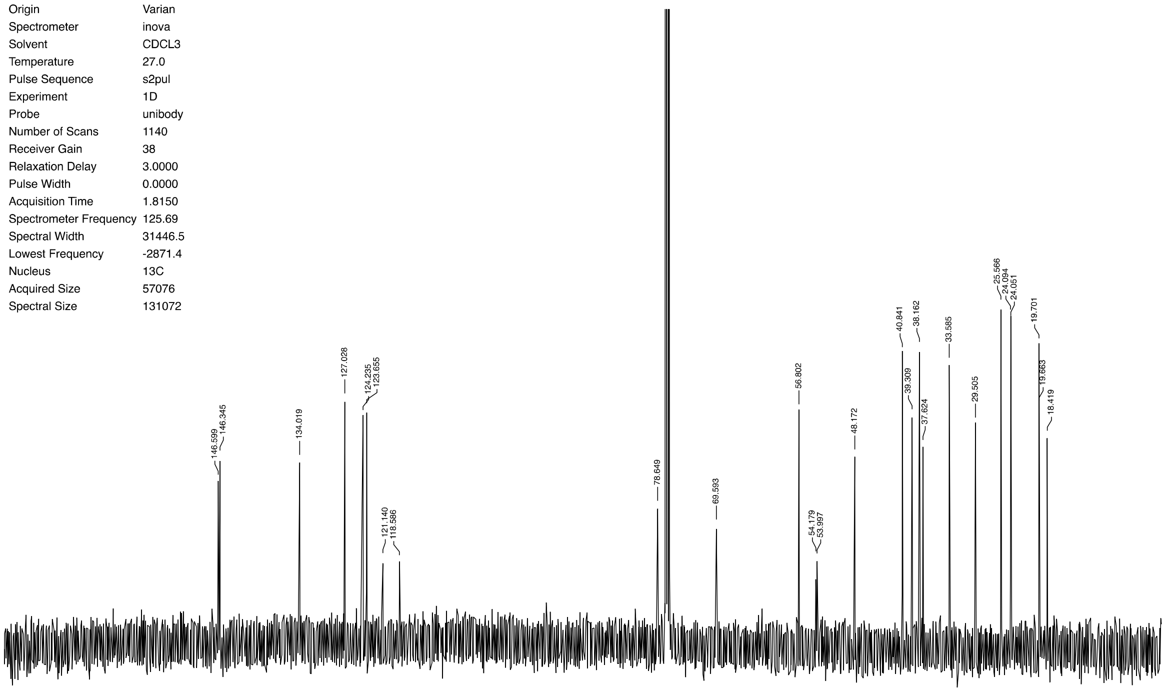

Figure S53. [13]C Spectra of Compound **9c**.

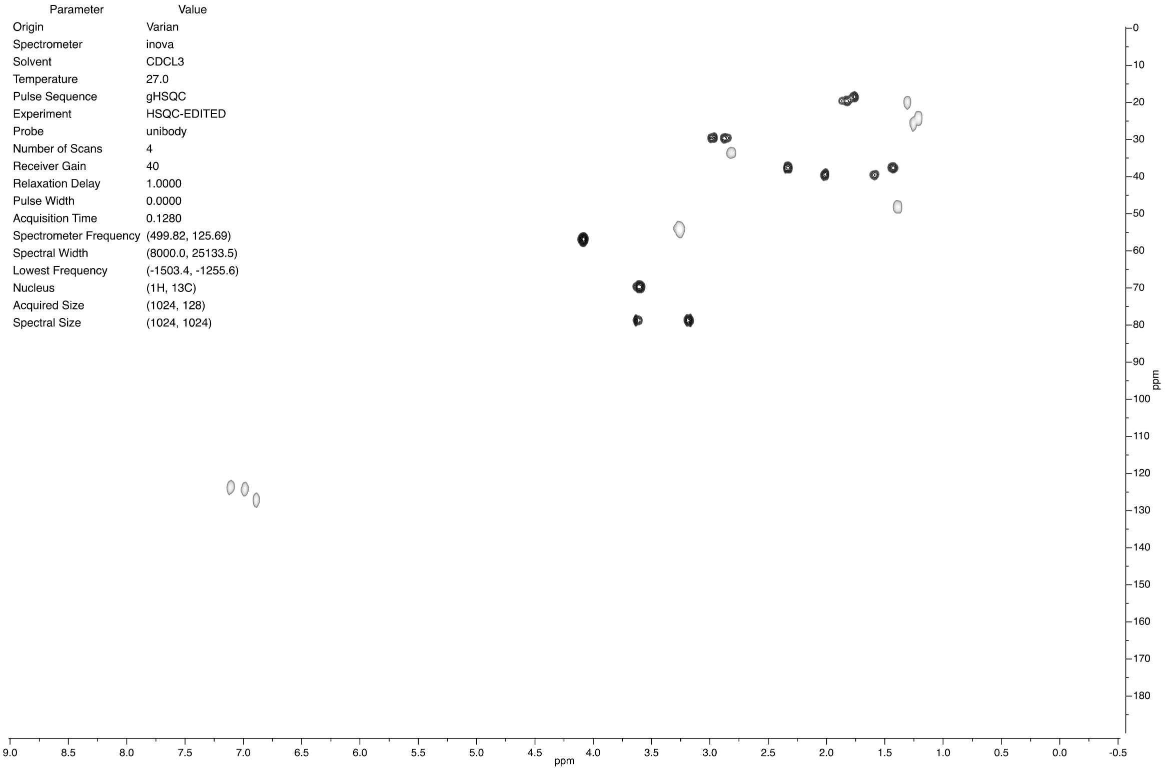

Figure S54. HSQC Spectra of Compound **9c**.

© 2015 by the authors; licensee MDPI, Basel, Switzerland. This article is an open access article distributed under the terms and conditions of the Creative Commons by Attribution (CC-BY) license (http://creativecommons.org/licenses/by/4.0/).
